# Novel Derivatives of 3-Amino-4-hydroxy-benzenesulfonamide: Synthesis, Binding to Carbonic Anhydrases, and Activity in Cancer Cell 2D and 3D Cultures

**DOI:** 10.3390/ijms26136466

**Published:** 2025-07-04

**Authors:** Valdas Vainauskas, Rugilė Norvaišaitė, Birutė Grybaitė, Rita Vaickelionienė, Alexey Smirnov, Tautvydas Kojis, Lina Baranauskiene, Elena Manakova, Saulius Gražulis, Asta Zubrienė, Daumantas Matulis, Vytautas Mickevičius, Vilma Petrikaitė

**Affiliations:** 1 Department of Organic Chemistry, Kaunas University of Technology, Radvilėnų Rd. 19, LT-50254 Kaunas, Lithuania; valdas.vainauskas@ktu.edu (V.V.); birute.grybaite@ktu.lt (B.G.); rita.vaickelioniene@ktu.lt (R.V.); 2 Department of Biothermodynamics and Drug Design, Institute of Biotechnology, Life Sciences Center, Vilnius University, Saulėtekio 7, LT-10257 Vilnius, Lithuania; rugile117@gmail.com (R.N.); alexey.smirnov@bti.vu.lt (A.S.); tautvydas.kojis@gmc.vu.lt (T.K.); lina.baranauskiene@bti.vu.lt (L.B.); asta.zubriene@bti.vu.lt (A.Z.); daumantas.matulis@bti.vu.lt (D.M.); 3 Department of Protein–DNA Interactions, Institute of Biotechnology, Life Sciences Center, Vilnius University, Saulėtekio al. 7, LT-10257 Vilnius, Lithuania; 4 Sector of Crystallography and Chemical Informatics, Institute of Biotechnology, Life Sciences Center, Vilnius University, Saulėtekio al. 7, LT-10257 Vilnius, Lithuania; saulius.grazulis@bti.vu.lt; 5 Institute of Biotechnology, Life Sciences Center, Vilnius University, Saulėtekio 7, LT-10257 Vilnius, Lithuania; 6 Laboratory of Drug Targets Histopathology, Institute of Cardiology, Lithuanian University of Health Sciences, Sukilėlių 13, LT-50162 Kaunas, Lithuania

**Keywords:** 3-amino-4-hydroxy-benzenesulfonamide, Schiff base, *β*-alanine, 2-pyrrolidinone, aminoketone, imidazole, cell viability, selectivity, tumor spheroids, carbonic anhydrase inhibitor, X-ray crystallography

## Abstract

A series of novel derivatives of 3-amino-4-hydroxybenzenesulfonamide was synthesized. As the analyzed compounds possess a sulfonamide group, the affinity of these compounds for human carbonic anhydrases (CAs) was measured by fluorescent thermal shift assay, and compound selectivity for different isoenzymes was identified. The crystal structures of the complexes of compound **25** with CAI and CAII were determined. Additionally, the activity of compounds on the viability of three cancer cell lines—human glioblastoma U-87, triple-negative breast cancer MDA-MB-231, and prostate adenocarcinoma PPC-1—was established using the MTT assay and compared to CAIX-selective and non-selective comparative compounds U-104 and acetazolamide. The half-maximal concentration (EC_50_) was determined for the identified most active compounds, and their selectivity over fibroblasts was established. Compound **9** (inhibitor of multi-CAs) and compound **21** (not binding to CAs), considered the most promising candidates, were tested in cancer cell 3D cultures (cancer spheroids) by assessing their effect on spheroid growth and viability. Both compounds reduced the viability of spheroids from all cancer cell lines. U-87 and PPC-1 spheroids became looser in the presence of compound **9**, while the growth of MDA-MB-231 spheroids was slower compared to the control. Compound **21** reduced the growth of U-87 and MDA-MB-231 3D cultures, with no significant effect on PPC-1 spheroids.

## 1. Introduction

The presence of both hydrophilic (hydroxyl, amino) and lipophilic (aromatic ring) regions in the molecule can improve the pharmacokinetic properties of its derivatives. This can result in better absorption, distribution, metabolism, and excretion (ADME) profiles. It can serve as a lead compound for the development of new therapeutic agents. The compound provides a useful scaffold for structure–activity relationship (SAR) studies to understand the relationship between chemical structure and biological activity, guiding the rational design of more effective drugs.

We have previously investigated para-substituted benzenesulfonamides containing modified 5-oxopyrrolidine fragments as carbonic anhydrase (CA) inhibitors [1]. Several compounds showed more potent and selective binding to CAIX. We subsequently examined the influence of the chlorine present at the *meta* position on the benzenesulfonamide ring on the interaction with CA isoenzymes. Chlorinated pyrrolidinone-based benzenesulfonamide derivatives bound more strongly than non-chlorinated compounds [2]. Later, we synthesized chlorinated benzenesulfonamides with dimethyl groups at the *ortho* positions with a 5-oxopyrrolidine linker at the *para* position, and such compounds possessed a stronger affinity for CAVB [3].

In this study, we report the synthesis of hydroxybenzenesulfonamides containing a modified Schiff base, *β*-alanine, aminoketone, imidazole, and 5-oxopyrrolidine fragments at the *meta* position, and their binding affinities for twelve catalytically active CA isoenzymes were evaluated by thermal shift assay. Variation of the substituent size at the 3-position of the 5-oxopyrrolidine fragment allowed us to modulate compound binding profiles to CA isoenzymes. For testing compounds’ biological activity in vitro, we chose three different human cancer cell lines based on their aggressiveness and distinct expression of CAs. The triple-negative breast cancer cell line is frequently used as a representative model for screening therapeutic candidates targeting this cancer subtype. It is known for its aggressive nature and high metastatic potential, and is associated with elevated rates of recurrence and mortality [4]. The glioblastoma U-87 cell line is one of the most widely used in vitro models for studying an aggressive and highly invasive form of primary brain tumor [5]. While prostate carcinoma is generally regarded as less fatal, it is the second most commonly diagnosed cancer among men [6]. The limited available treatment for those types of cancer shows a need for more effective and safer alternatives. Moreover, the MDA-MB-231 cell line is often used as an in vitro model for testing the activity of carbonic anhydrase inhibitors, as it is characterized by CAIX expression [7]. Variable expression of different isoforms of CAs has been established in glioblastoma [8]. However, it was shown that the inhibition of CAII in the U-87 cell line could lead to lower resistance to anticancer drugs [9]. On the contrary, the expression of CAIX was not detected in hypoxic prostate tumors [10]; thus, we added the PPC-1 cell line for comparison of compound activity in three different in vitro models with distinct expression of CAs.

In addition, it is known that cell surface transmembrane CAIX and CAXII are usually expressed at higher levels in a hypoxic tumor environment compared to normal tissues [11,12,13]. This makes those two CAs attractive targets for anticancer drug development [14]. It is known that 3D cultures better mimic the tumor microenvironment than the cells in monolayers, including the hypoxic core and metabolic gradient [15]. Usually, hypoxia develops in spheroids exceeding 300–400 μm in diameter [16]. Thus, we tested selected compounds in tumor spheroids of such a size after their formation by a magnetic 3D bioprinting method. For this study, we chose the most active compound binding to many CAs and another one not binding to CAs but relatively highly selective towards cancer cells over fibroblasts.

This study aimed to explore the differences in the biological activity of 3-amino-4-hydroxy-benzenesulfonamides with different binding to CAs in a cancer cell monolayer (2D) and cancer spheroids (3D cultures), taking into consideration the diverse expression of CAs in selected cancer cell lines and different conditions (normoxia and hypoxia). This could help to better understand how much the selectivity towards specific CAs is important in achieving higher activity in more tumor-relevant in vitro models.

## 2. Results and Discussion

### 2.1. Chemistry

Sulfanilamides are known as efficient chemotherapeutic agents for preventing and treating human bacterial infections by disrupting bacterial, but not human, metabolism [17]. 3-Amino-4-hydroxybenzenesulfonamide is a reagent used to synthesize antagonists for somatostatin receptor subtype 5 (SST5R). It also has utility in dye applications [18,19].

Schiff bases are a large class of compounds characterized by the presence of a C=N double bond, which is essential to their biological properties. They can be found in nature or synthesized in the laboratory [20,21,22] and are reported to exhibit a broad range of biological activities, namely antibacterial [21,23], antifungal [24,25], antimalarial [26], antiproliferative, anti-inflammatory, antiviral, and antipyretic properties [27,28].

Imidazole is a constituent of various chemical structures in living organisms, including alkaloids, biotin, histamine, histidine, and nucleic acid [29]. Imidazole compounds were found to possess a diversity of pharmacological properties, like anticancer, antioxidant, anti-inflammatory, gastroprotective, histamine-H3 antagonist, and antiparasitic [29,30]. Imidazole is the core of approved drugs with acceptable activities in practice. Many different compounds with an imidazole core have been used in medicine in clinical trials for various diseases, where they have shown strong therapeutic properties. The growing trend of imidazole-based medicinal chemistry has added promising and potential therapeutic values of imidazole compounds in the treatment of incurable diseases [31]. Compared to the other heterocycles, the imidazole derivatives provide electron-rich characteristics responsible for binding with a variety of enzymes, proteins, and receptors. All this attracts the attention of medicinal chemistry researchers to explore the imidazole core in the study of new, improved therapeutic properties.

**Scheme 1:**
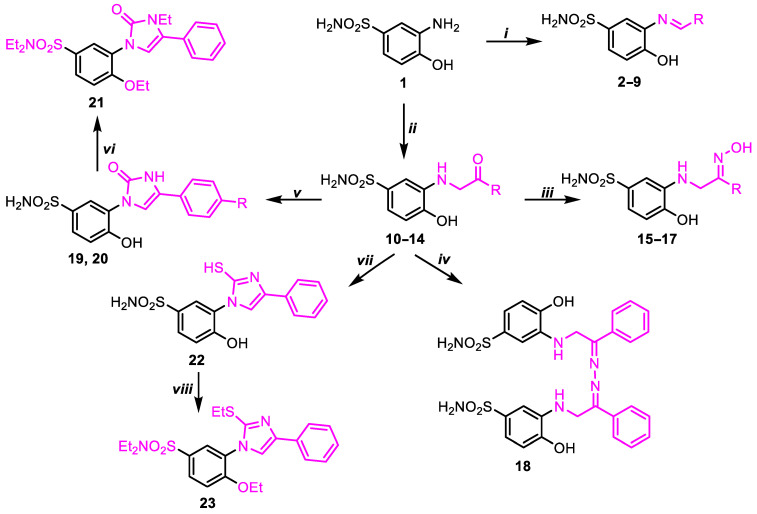
Synthesis of Schiff bases **2**–**9** and *a*-amino ketones **10**–**14**, and chemical transformations of the latter compounds into derivatives **15**–**23**. **2**, **10**, **15**, **19** R = Ph; **3** R = 4-FPh; **4**, **12**, **17** R = 4-ClPh; **5** R = 4-MeOPh; **6, 14** R = 1-naphthyl; **7** R = 2-naphthyl; **8** R = thien-2-yl; **9** R = 5-NO_2_-thien-2-yl; **11** R = 2,4-diFPh; **13**, **16**, **20** R = 4-HOPh. *Reagents and conditions: i* corresponding aldehyde, 2-PrOH, Δ, 1 h; *ii* corresponding bromoacetophenone, H_2_O:2-PrOH, Δ, 4 h; *iii* MeOH, NH_2_OH_·_HCl, AcONa·3H_2_O, Δ, 4 h; *iv* MeOH, N_2_H_4_·H_2_O, Δ, 2 h; *v* carbamide, gl. AcOH, Δ, 24 h, H_2_O; *vi* and *viii* DMSO, KOH, K_2_CO_3_, EtI, Δ, 24 h; *vii* resynthesized according to Ref. [32].

In the present study, Schiff bases were synthesized by the standard procedure (Scheme 1) starting from 3-amino-4-hydroxybenzenesulfonamide, which was condensed with the appropriate aromatic aldehyde in propan-2-ol at reflux for 1 h. The products were isolated in good to excellent yields of 57–95%. The structures of derivatives **2**–**5** were easily identified by the proton singlet of the N=CH group in the range of 8.62–8.74 ppm. The peak of the proton of this group for more bulky compounds **6** and **7** arose at 9.29 and 8.89 ppm, respectively, and the thiophene-bearing derivatives **8** and **9** showed this proton as the singlets at 8.85 and 9.04 ppm, respectively (Supplementary files, Figures S1–S16).

As part of our ongoing program, the synthesis of compounds with a 2-oxoethylamine core was aimed for. Starting from readily available primary amine **1**, its reaction with the corresponding bromoacetophenone afforded target compounds **10**–**14**. The reactions were performed in a mixture of water and propan-2-ol in a ratio of 1:1 at reflux for 4 h. The structures of the products were easily identified from their ^1^H NMR and ^13^C NMR spectra (Supplementary files, Figures S17–S24).

Next, obtained compounds **10**–**14** were applied for the synthesis of (2-(hydroxyimino)-2-arylethyl)amino derivatives **15**–**17**, the dimer **18**, and imidazoles **19**–**23** (Scheme 1).

To obtain ketoximes **15**–**17,** the corresponding compounds **10**, **12**, and **13** were treated with a two-fold excess of hydroxylamine hydrochloride in methanol at reflux in the presence of sodium acetate in the mixtures. The products were isolated in mixtures of *Z* and *E* isomers in a ratio of 80:20 for all ketoximes (Supplementary files, Figures S17–S30).

Azines having a molecular structure of ArAr^1^C=N–N=CAr^1^Ar are of interest because of their wide range of therapeutic effects [33]. The general mechanism for obtaining the azine structure is the addition of hydrazines to the carbonyl compound to form a hydrazone, which can be followed by reaction with a second molecule of the carbonyl compound, which results in an azine derivative [34]. To prepare such a dimer, a mixture of compound **10** and hydrazine monohydrate in a 2:1 molar ratio was refluxed in methanol for 2 h. The product 3,3′-((hydrazine-1,2-diylidenebis(2-phenylethan-1-yl-2-ylidene))bis(azanediyl))bis(4-hydroxybenzenesulfonamide) (**18**) was obtained in an excellent yield of 92%. The NMR, IR, and mass spectral data fully confirmed the expected dimer structure (Supplementary files, Figures S31 and S32).

5-Oxo(thioxo)imidazolone, being a privileged molecule because of its biological potency and as an important pharmacophore in modern drug discovery, attracted attention to explore such rings for their new activities.

To obtain the imidazol-2-one derivatives **19**, **20**, the corresponding precursors **10** and **13** were cyclized with a four-fold excess of carbamide in glacial acetic acid. The reaction was carried out at reflux for 24 h, then the mixture was cooled and diluted with water to form a crystalline solid. In the ^1^H NMR spectra for **19** and **20**, the singlets at 10.85 and 11.11 (for **19**) as well as at 10.84 and 11.00 (for **20**) ppm were assigned to the protons in the OH and NH groups, respectively; the proton of the p-*H*OPh of compound **20** resonated at 9.57 ppm. The proton of the imidazole CH was observed at 7.84 (**19**) and 7.83 (**20**) ppm. Moreover, the resonances in the ^13^C NMR spectra at 109.71 and 107.28 ppm were attributed to the imidazole CH, and the spectral lines at 154.82 as well at 154.65 ppm demonstrated the presence of a carbonyl group in the imidazole ring and approved the formation of 2-oxoimidazole moiety in molecules **19** and **20** (Supplementary files, Figures S33 and S34).

When compound **10** was reacted with potassium thiocyanate in a mixture of glacial acetic acid and hydrochloric acid (5:1) at reflux for 4 h, the thio analogue **22** was isolated from the reaction mixture (resynthesized according to Ref. [32]). Interestingly, compounds **19** and **20** were obtained in keto tautomeric form, while thio derivative **22** appeared to be in thiol form, showing the singlet at 12.92 ppm for SH in the ^1^H NMR spectrum. All other peaks were in excellent agreement with those given in Ref. [32].

To increase the diversity of synthesized compounds, keto- (**19**) and thiol- (**22**) tautomers were alkylated with a large amount of ethyl iodide. The reaction was performed in DMSO at reflux for 24 h. The product was isolated by adding water to the cooled reaction mixture. The ^1^H NMR spectrum of compound **21** reconfirmed the presence of the keto form of compound **19** with an NHC=O fragment in the structure. In the ^1^H NMR spectrum for **21**, the absence of the singlet for the proton of the NH group at 11.00 ppm and the rise of the signals of 1.19–0.96 (m, 9H, CH_3_) and 1.34 (t, *J* = 6.9 Hz, 3H, CH_3_), as well 3.16 (q, *J* = 7.0 Hz, 4H, 2CH_2_), 3.76 (q, *J* = 6.9 Hz, 2H, CH_2_), and 4.19 (q, *J* = 6.9 Hz, 2H, CH_2_), confirmed complete ethylation of the SO_2_NH_2_, OH, and NH groups in the structure (Supplementary files, Figures S35 and S36).

In the ^1^H NMR spectrum for **23**, the singlet for the SH proton at 12.92 ppm disappeared, and three triplets at 1.05, 1.21, and 1.26 ppm integrated for 6H, 3H, and 3H and three quadruplets at 3.00, 3.19, and 4.20 ppm integrated for 2H, 4H, and 2H correspond to the protons of the ethyl groups providing the N(CH_2_CH_3_)_2_, OCH_2_CH_3_, and SCH_2_CH_3_ fragments in the molecule (Supplementary files, Figures S37 and S38).

**Scheme 2:**
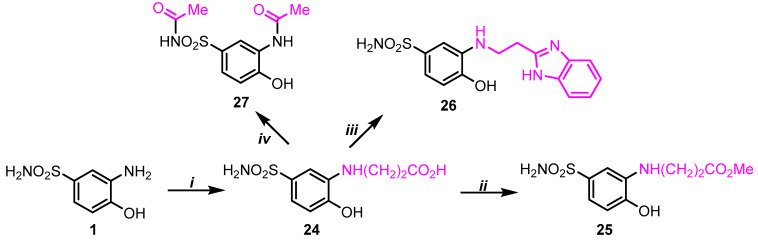
Synthesis of *β*-amino acid **24** and its derivatives **25**–**27**. *Reagents and conditions: i* acrylic acid, H_2_O, Δ, 10 h; *ii* MeOH, H_2_SO_4_, Δ, 5 h; *iii* benzene-1,2-diamine, 4M HCl, Δ, 24 h, 25% ammonia to pH 8; *iv* Ac_2_O, H_2_SO_4_, Δ, 5 h, 5% Na_2_CO_3_.

**Scheme 3:**
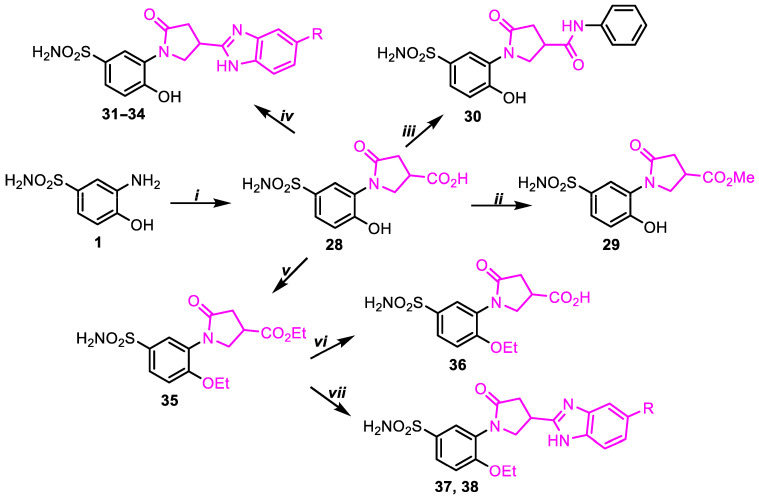
Synthesis and chemical transformations of 5-oxopyrrolidine-3-carboxylic acid **28**. **31**, **37** R = H; **32** R = Me; **33** R = F; **34**, **38** R = Cl. *Reagents and conditions: i* itaconic acid, H_2_O, reflux, 4 h; *ii* CH_3_OH, H_2_SO_4_, reflux, 8 h; *iii* aniline, HBTU, DMF, r.t. 24 h, H_2_O; *iv* the corresponding benzene-1,2-diamine, 4M HCl, Δ, 24 h, boiling H_2_O, 25% ammonia to pH 8; *v* DMF, KOH, K_2_CO_3_, EtI, r.t., 14 h, H_2_O; *vi* 10% HCl, Δ, 2 h; *vii* the corresponding benzene-1,2-diamine, 4M HCl, Δ, 24 h, boiling H_2_O, 25% ammonia to pH 8 (**37**); Δ, 24 h, 25% ammonia to pH 8 (**3**,**8**).

To assess the impact of *β*-alanine and 3-carboxy-5-oxopyrrolidine fragments on the bioactivity of compounds, 3-((2-hydroxy-5-sulfamoylphenyl)amino)propanoic acid (**24**) as well as 1-(2-hydroxy-5-sulfamoylphenyl)-5-oxopyrrolidine-3-carboxylic acid (**28**) and their derivatives (Scheme 2 and Scheme 3) were prepared and then examined against selected cancer cells.

As expected, treatment of amine **1** with acrylic acid in water at reflux for 10 h afforded compound **24** (Scheme 2). In the present case, aromatic amine 1 adds to the α,β-unsaturated bond via a conjugate (Michael-type) addition, affording the corresponding N-substituted β-amino acid 24, which was then reacted with methanol in the presence of a sulfuric acid catalyst to obtain methyl ester **25**. The structures of the synthesized acid **24** and its methyl ester **25** were characterized by spectroscopic methods (Supplementary files, Figures S39–S42) and microanalysis data, which fully confirmed the presence of the designed products.

To introduce the benzimidazole core in the molecule, acid **24** was heated at reflux with benzene-1,2-diamine in 4M hydrochloric acid. After completion of the reaction, the dropwise addition of an aqueous 25% ammonia to pH 8 gave the desired structure **26**. From the ^1^H NMR spectrum, a broad singlet at 12.30 ppm proved the presence of the NH group of the benzimidazole structure in the molecule. The appearance of additional signals in the aromatic area indicated the presence of a new aromatic ring. In the ^13^C NMR spectrum, instead of the C=O line at 173.41 ppm, a characteristic carbon resonance at 153.18 ppm for the N=C-NH group of benzimidazole was found.

Afterwards, it was decided to form a cyclic product; thus, acid 24 was treated with acetic anhydride as a dehydrating agent, but cyclization did not occur. In this reaction, we initially expected the formation of 2-oxo-2,3,4,5-tetrahydrobenzo[b][1,4]oxazepine-7-sulfonamide; however, the actual outcome deviated from this pathway, and the *N*-acetylated product *N*-((3-acetamido-4-hydroxyphenyl)sulfonyl)acetamide (**27**) was isolated from the reaction mixture and fully characterized by the methods of IR and NMR (Supplementary files, Figures S43–S46) spectroscopy and microanalysis data.

In the last stage of the study, a library of 1-(2-hydroxy-5-sulfamoylphenyl)-5-oxopyrrolidine-3-carboxylic acid derivatives were prepared by incorporating alkyl, amino, ethereal, and heterocyclic fragments into the molecular structure (Scheme 3).

Refluxing 4-hydroxy-3-aminobenzenesulfonamide (**1**) with itaconic acid afforded carboxylic acid **28**, which was subsequently transformed into a series of derivatives. Pyrrolidine-3-carboxylic acid **28** was then reacted with methanol in the presence of a sulfuric acid catalyst to obtain methyl ester **29** (Supplementary files, Figures S47–S52), and with aniline in DMF in the presence of triethylamine at room temperature for 24 h to afford amide **30**.

Benzimidazoles **31**–**34** were synthesized from a reaction of acid **28** and the appropriate benzene-1,2-diamine according to the commonly used method as for the preparation of **26** and fully characterized by the methods of the IR and NMR (Supplementary files, Figures S53–S60) spectroscopy and microanalysis data.

After that, it was decided to carry out the alkylation reaction of acid **28** with ethyl iodide. The reaction in DMF at ambient conditions for 14 h and in the presence of a strong base potassium hydroxide and potassium carbonate as an agent to maintain an anhydrous medium gave the *O*-alkylated product **35**. The additional spectral lines of the aliphatic protons are visible in the ^1^H NMR spectrum of the synthesized compound. Two triplets at 1.22 and 1.33 ppm, which are integrated for three protons each and a multiplet from 4.00 to 4.31 ppm, which was integrated for four protons, showed the presence of 2C_2_H_5_ groups included in the molecule. The remaining intense singlet at 7.30 ppm indicated that ethylation of the NH_2_ group did not occur in this case. In the ^13^C NMR spectrum, the resonance lines at 14.04 and 14.40 ppm for 2CH_3_ as well as 60.80 and 64.41 ppm for 2CH_2_ fully confirmed the presence of two ethyl groups (Supplementary files, Figures S61 and S62).

Compound **35** was further refluxed in 10% hydrochloric acid for 2 h, thereby cleaving the ester bond and affording the carboxylic acid **36**. In the ^1^H NMR spectrum of this compound, the remaining triplet at 1.34 ppm and quadruplet at 4.14 ppm are proton signals of the CH_2_CH_3_ group, and the deshielded proton singlet at 12.73 ppm, which was assigned to the carboxyl group, indicated a compound with a free carboxyl group. This was supported by the ^13^C NMR as well. Spectral lines at 14.42 and 64.42 ppm are signals of the carbons of the ethyl group incorporated into the structure of the molecule through the oxygen atom, and the peak at 174.27 ppm shows the restored COOH (Supplementary files, Figures S63 and S64).

Finally, the *O*-ethylated product **35** was applied to synthesize benzimidazoles **37** and **38**, using benzene- and 4-chlorobenzene-1,2-diamines. The spectral and microanalysis data were in excellent agreement with the structures obtained (Supplementary files, Figures S65–S69).

### 2.2. Compound Binding to Human Carbonic Anhydrases

We measured the affinities of the synthesized compounds for recombinant human carbonic anhydrases (CAs) by fluorescent thermal shift assay (FTSA). The FTSA method (also referred to as differential scanning fluorimetry, DSF) quantifies protein–small molecule binding affinity according to the ligand-induced increase in protein stability.

**Figure 1:**
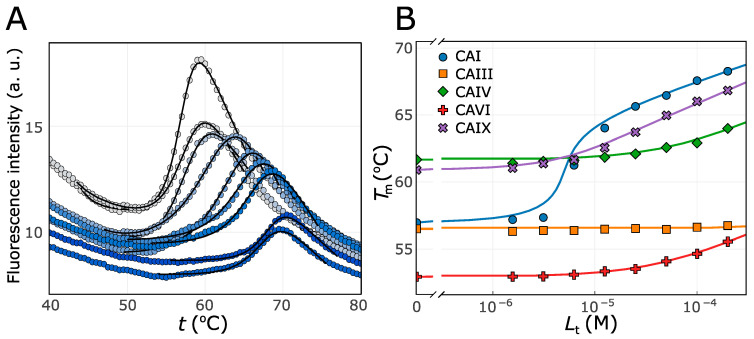
Example of binding affinity data obtained by FTSA: (**A**) raw fluorescence data of the CAI interaction with 0–200 µM of compound **12**, (**B**) the interaction of compound **12** with five different human CA isoenzymes, and determination of the *K*_d_ according to the *T*_m_ dependence on the added ligand concentration. Symbols indicate experimental values, and lines represent models used for *T*_m_ (**A**) and *K*_d_ (**B**) determination [36]. The color lines in (**B**) fitted with 1:1 stoichiometry, represent different CA isozymes: blue—CAI, orange—CAIII, green—CAIV, red—CAVI, and violet—CAIX.

Figure 1A shows an example of the primary data obtained in this work by FTSA: the interaction of the protein CAI with compound **12**. The intensity of the blue color of the symbols indicates an increase in the concentration of the added compound, i.e., the grey color corresponds to the unfolding curve of the ligand-free protein, and the dark blue color indicates the highest concentration of the compound. The symbols denote the experimental measurement points in the graph, and the lines represent the model used to determine the melting point (*T*_m_). With increasing concentration of the added compound, the *T*_m_ increases from about 56 to 66 °C. Figure 1B shows the *T*_m_ dependence of the added ligand concentration of five recombinant human carbonic anhydrase isoenzymes (CAI, CAIII, CAIV, CAVI, and CAIX). In this example, the analyzed compound does not bind to CAIII in the range of concentrations tested (up to 200 µM), weakly binds to CAIV and CAVI (with *K*_d_ 17 µM and 20 µM, respectively), binds to CAIX with a *K*_d_ of 0.98 µM, and most strongly binds to CAI with a *K*_d_ of 6.2 nM. Although there is no direct correlation between the change in *T*_m_ and the strength of the interaction [35], the general trend is that ligands that bind more strongly are more likely to stabilize the protein.

The dissociation constants (*K*_d_) were determined using the Thermott online tool [36]. Table 1 shows the *K*_d_ values (μM) determined for the interaction of all the compounds studied with human carbonic anhydrase isoenzymes at 37 °C. Control compounds acetazolamide (AZM) and U-104 were used in the study.

The analyzed 3,4-diphenyl-substituted benzenesulfonamides were categorized into five groups based on structural similarity. The structure-CA affinity analysis of these compounds is discussed below.

Schiff base compounds

**Figure 2:**
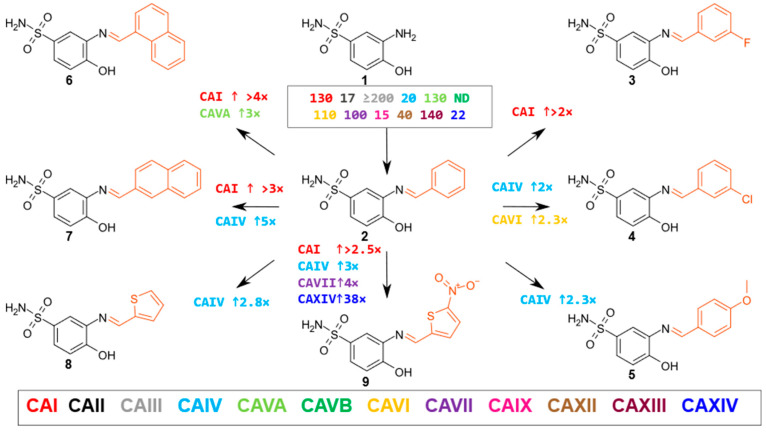
The Schiff base group consists of eight compounds based on 4-hydroxy-benzenesulfonamide, with different Schiff bases at the *meta*-position. The alternating part of each compound is highlighted in red. The numbers near the arrows indicate the number of times the affinity for a given isoenzyme is increased by comparing the compounds in the direction of the arrow (↑ indicates an increase, ↓ a decrease in affinity; only cases where the affinity differs by ≥2-fold are marked). The numbers in the box indicate *K*_d_ (µM) of compound **1**. Prepared using Marvin Sketch software version 25.1.58 [38].

The Shiff base group comprised eight compounds based on 4-hydroxybenzenesulfonamide. The parent compound **2** in this group, with a phenyl substituent, had a moderate affinity for CAII, CAIV, CAIX, and CAXIV isoenzymes, with *K*_d_ values of 16–28 μM and weaker binding to others. Compounds with different substituents showed minor changes: compound **4** with a 4-chlorophenyl substituent decreased the *K*_d_ value to 11–22 μM. However, compounds **3** and **5**, with 3-fluorophenyl and 4-methoxyphenyl substitutions, respectively, did not have a significant effect on the affinity (Figure 2).

Introducing the naphthyl group into the compound structures (compounds **6** and **7**) resulted in a better binding to CAI (*K*_d_ = 47–59 μM) than the other compounds in this group, but the overall affinity was not strong. The most significant changes were observed in the interaction of compound **6** with CAVA (*K*_d_ = 37 μM), while compound **7**, with a 2-naphthyl substituent, had the strongest interaction with the CAIV isoenzyme (*K*_d_ value 5.5 μM), with which the affinity was increased by a factor of five compared to the initial compound **2**. The substitution of the phenyl ring by thiophene (compound **2** vs. **8**) did not significantly affect the affinity towards all CAs (affinity increased up to 3 times only for CAIV). However, compound **9**, with nitrothiophene moiety, bound more strongly than the parent compound **2** to the isoenzymes CAI, CAIV, and CAVII (*K*_d_ values of 9 to 21 μM), and up to 38 times increased affinity was observed for CAXIV (*K*_d_ value 0.65 μM).

Aminoketones and their derivatives

**Figure 3:**
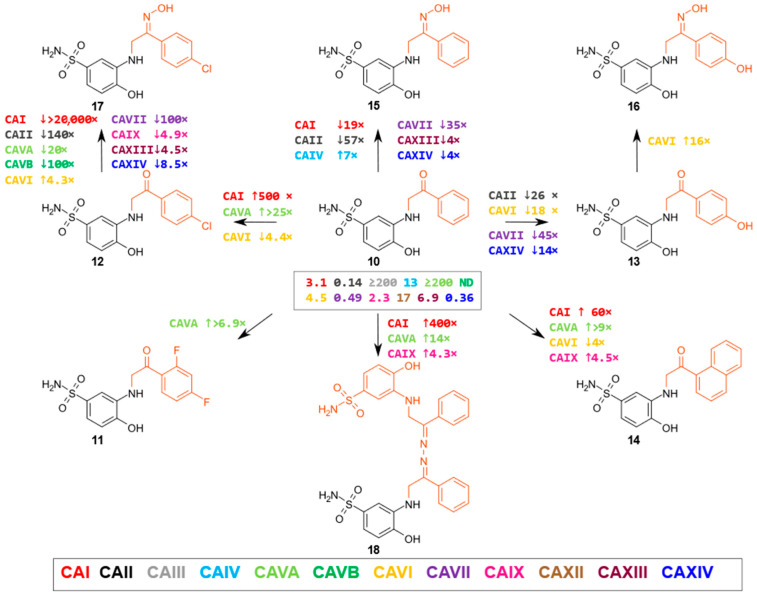
A group of aminoketones and their derivatives. This group consists of nine compounds based on 3-amino-4-hydroxybenzenesulfonamide, with different amino ketone derivatives formed at the *meta*-position. The numbers near the arrows indicate the number of times the affinity changes (↑ indicates an increase and ↓ indicates a decrease) for a given isoenzyme when comparing the compounds in the direction of the arrow (cases where the affinity differs by ≥4-fold are marked). The numbers in the box indicate the *K*_d_ (µM) of compound **10**.

A group of aminoketones and their derivatives consists of nine compounds based on 3-amino-4-hydroxybenzenesulfonamide, with different aminoketone fragments. The substituent changes in this group significantly affected the observed affinity for different CA isoenzymes (Figure 3). Aminoketone **10**, containing a phenyl substituent, had the strongest affinity for CAI, CAII, CAVII, CAIX, and CAXIV isoenzymes (*K*_d_ values in the range of 0.14–3.1 μM). The 4-chlorophenyl substituent in compound **12** notably enhanced the binding to CAI up to 500 times (*K*_d_ 6.2 nM) and to CAVA up to 25 times (*K*_d_ 8 µM) in comparison to compound **10** (*K*_d_ for CAI and CAVA is 3.1 and ≥200 µM, respectively), and 2,4-difluorophenyl- and 4-hydroxyphenyl- substituents (**11** and **13**, respectively) weakened the binding of the majority of these CAs in comparison to compound **10** (*K*_d_ values of 0.15 to 6 μM and 3.6 to 22 μM, respectively). Replacement of the phenyl substituent (compound **10**) by a 1-naphthyl substituent (compound **14**) increased the affinity for the CAI by 60 times, and for CAVA and CAIX isoenzymes by 9 and 4.5 times.

Ketoximes (**15**, **17**, **16**) derived from amino ketones (**10**, **12**, **13**) showed the strongest binding to CAVI (*K*_d_ = 2.4–5.1 μM), CAIX (*K*_d_ = 1.5–4.8 μM), and CAXIV (*K*_d_ = 1.6–2.9 μM) isoenzymes. Compounds **15** and **16** were among the most effective for CAIV (*K*_d_ = 1.9 and 3 μM, respectively) of the whole series of tested compounds. One compound in this group stood out in terms of its structure as a dimer (compound **18**). It was a strong binder to CAI, CAII, CAVII, CAIX, and CAXIV isoenzymes, with *K*_d_ values in the range of 0.0077 to 0.53 μM.

Oxoimidazoles

**Figure 4:**
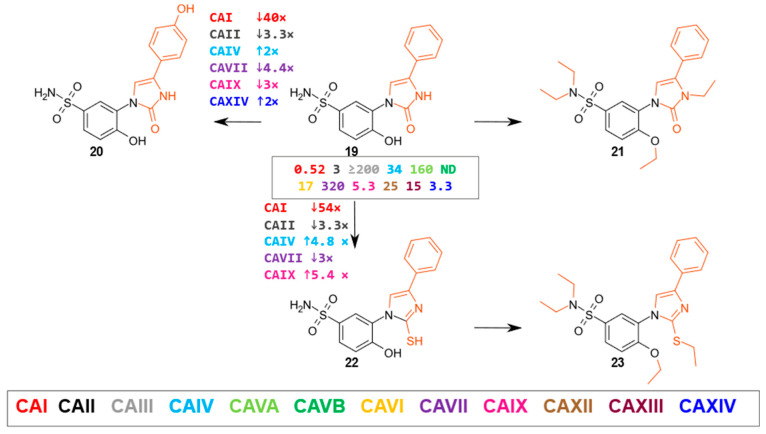
4-Hydroxybenzenesulfonamides containing an imidazole fragment with a phenyl substituent at the *meta*-position. The numbers near the arrows indicate the fold change in affinity (↑ indicates an increase and ↓ a decrease) for a given isoenzyme when comparing the compounds in the direction of the arrow (cases where the affinity differs by ≥2-fold are marked). The numbers in the box indicate the *K*_d_ (µM) of compound **19**.

This group consisted of five compounds—three 4-hydroxybenzenesulfonamides (compounds **19**–**21**) and two 4-ethoxybenzenesulfonamides (**22**, **23**) containing a phenylsubstituted oxoimidazole fragment (Figure 4, compounds **19** to **23**, alternating group in red).

Compound **19**, with a phenyl substituent, bound most strongly to CAI (*K*_d_ = 0.52 μM), CAII (*K*_d_ = 3.0 μM), CAIX (*K*_d_ = 5.3 μM), and CAXIV (*K*_d_ = 3.3 μM). The introduction of a hydroxy group in the para-position of the phenyl ring (compound **20**) most strongly reduced binding affinity for CAI (about 40–fold). Compound **21,** bearing an alkylated tertiary sulfonamide group, completely lost its ability to bind CA isoenzymes.

2-Thioimidazole **22** bound strongly to CAIX (*K*_d_ = 0.98 μM) and moderately to CAII (*K*_d_ = 9.8 μM), CAIV (*K*_d_ = 7.1 μM), CAXIII (*K*_d_ = 14 μM), and CAXIV (*K*_d_ = 5.9 μM) isoenzymes. *N*-alkylated sulfonamide, compound **23,** lost its affinity to CA isoenzymes.

Comparing two analogous compounds 2-oxoimidazole **19** and 2-thioimidazole **22**, we observed that the compound containing the oxo group bound about fifty times more strongly to CAI (*K*_d_ = 0.52 μM) and about three times more strongly to CAII (*K*_d_ = 3.0 μM) and CAVII (*K*_d_ = 32 μM). In contrast, the compound containing the thiol group had about five times stronger binding to the isoenzymes CAIV (*K*_d_ = 7.1 μM) and CAIX (*K*_d_ = 0.98 μM).

*β*-Amino acids and their derivatives

**Figure 5:**
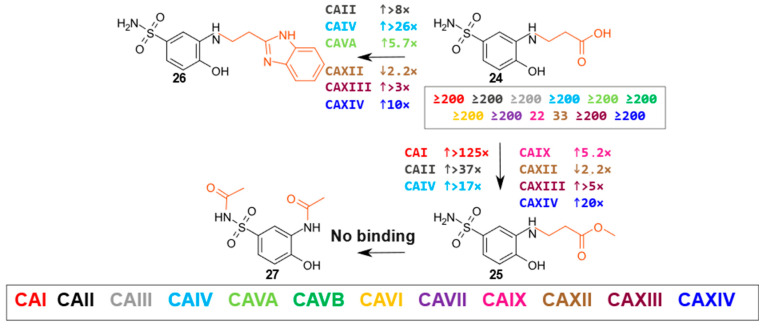
Compounds of the β-amino acid group and the diacetylated derivative of compound 1. The numbers near the arrows show the fold change in affinity (↑ indicates an increase and ↓ a decrease) for a given isoenzyme when comparing the compounds in the direction of the arrow (cases where the affinity differs by ≥2-fold are marked). The numbers in the box indicate the *K*_d_ (µM) of compound **24**.

There were four compounds in this group (compounds **24**–**27** in Figure 5, alternating group marked in red). Carboxyl-containing compound **24** bound weakly only to CAIX and XII, (*K*_d_ values of 22 μM and 33 μM, respectively), whereas the methyl ester (compound **25**) showed an increased affinity of more than 100-fold for CAI, nearly 40-fold for CAII, about 20-fold for CAIV and CAXIV, and about 5-fold for CAIX and CAXIII. The benzimidazole fragment (compound **26**) increased the affinity for CAI, CAII, CAVA, CAIX, and CAXIV isoenzymes compared to the carboxyl group containing compound **24**. Still, with most CAs, **26** had a weaker binding than the methyl ester **25** discussed above. Diacetylated compound **27** did not interact with CA isoenzymes.

3-substituted-5-oxopyrrolidine derivatives

**Figure 6:**
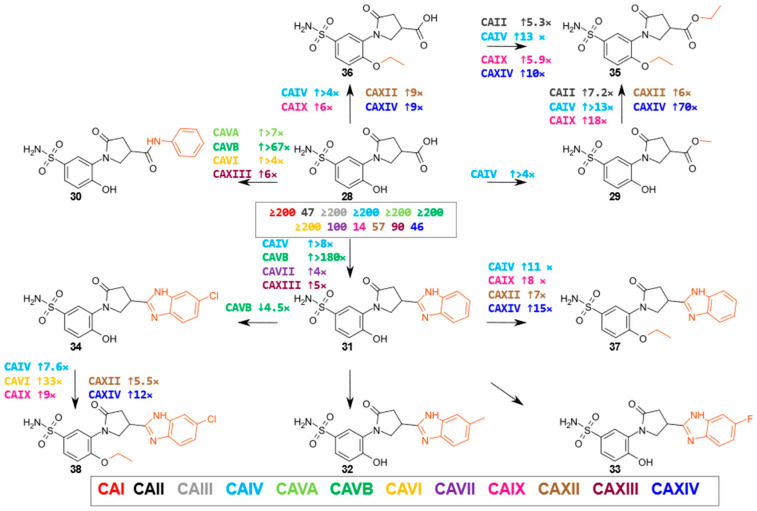
5-Oxopyrrolidine-3-carboxylic acid derivatives. The numbers near the arrows indicate the fold change in affinity (↑ indicates an increase and ↓ a decrease) for a given isoenzyme when comparing the compounds in the direction of the arrow (cases where the affinity differs by ≥4-fold are marked). In cases where there are no numbers next to the arrows, the affinities of the two compounds being compared do not differ by more than 4-fold for all isoenzymes. The numbers in the box indicate the *K*_d_ (µM) of compound **28**.

This group consisted of eleven compounds, 4-hydroxybenzenesulfonamide derivatives containing 3-substituted-5-oxopyrrolidine fragment (compounds **28**–**38** in Figure 6, with the alternating group marked in red). The 5-oxopyrrolidine-3-carboxylic acid fragment of the primary compound **28** had a weak affinity for CA isoenzymes. However, a higher selectivity for the CAIX isoenzyme was observed (*K*_d_ = 14 μM), and modification of the compound to the methyl ester **29** enhanced the binding (*K*_d_ = 7.1 μM), with an approximately four-fold increase in the affinity for CAIV. Ethylation of the hydroxyl group of compound **28** (compound **36**) enhanced the interaction with most CA isoenzymes, while modification of the carboxyl group to an ester (compound **35**) further increased the affinity, e.g., *K*_d_ = 0.39 μM for CAIX and *K*_d_ = 0.48 μM for CAXIV.

The specificity for CA isoenzymes changed quite significantly with the modification of parent compound **28** into amide **30**. Although the overall affinity for CA isoenzymes remained weak, binding to the CAVB isoenzyme increased most (from *K*_d_ ≥ 200 µM to *K*_d_ 3 μM), while interactions with the CAVA, CAVI, and CAXIII isoenzymes also became stronger.

Compounds **31**–**34**, with benzimidazole fragments, showed a slightly stronger affinity for CA isoenzymes than parent compound **28**, with the highest affinity and selectivity of **31** for the CAVB isoenzyme (*K*_d_ = 1.1 μM). Compounds **32**–**34** with methyl, fluorine, and chlorine substituents at the fifth position of the benzimidazole ring interacted with most isoenzymes with similar strengths.

The modification of the hydroxyl group of compounds **28**, **31**, and **34** to the ethoxy group yielded compounds **36**, **37**, and **38**, respectively, and we observed an increase in the affinity for many isoenzymes, most notably CAIV, CAVI, CAIX, CAXII, and CAXIV. For example, compound **31** with a hydroxy group bound more weakly to CAIV, CAIX, and CAXIV (*K*_d_ = 24 μM, 9.7 μM, 14 μM, respectively) than compound **37** with an ethoxy group (*K*_d_ = 2.2 μM, 1.2 μM, 0.94 μM, respectively). Analogous changes in affinity were seen with compounds **28** and **36** and compounds **34** and **38**. We also observed that the carboxy group-containing compound **36** (*K*_d_ = 2.3 μM, 5.0 μM, respectively) bound more weakly than its ethyl ester **35** (*K*_d_ = 0.39 μM, 0.48 μM, respectively). In addition, compound **35** had the strongest affinity for CAIX and CAXIV isoenzymes of all the compounds in this group. Similarly, compound **28**, with a carboxy group, has a weaker binding affinity than its methyl ester, **29**.

In summary, this group of compounds did not bind to CAI or CAIII and bound weakly to CAII, CAVA, CAVII, CAXII, and CAXIII. Structure–activity analysis shows that the compounds containing the ether group bound better than the others to CAIV, CAIX, and CAXIV. On the other hand, 4-hydroxybenzenesulfonamide compounds containing a benzimidazole fragment exhibited a strong interaction with the CAVB isoenzyme (*K*_d_ = 1.1–4.9 μM).

Structure–activity analysis of the compounds studied shows that the addition of additional groups (e.g., ethyl, acetyl) to the primary sulfonamide fragment abolished the ability of the compounds (**21**, **23**, **27**) to bind to CA isoenzymes.

V. Alterio et al. [39] conducted a study on 4-mercaptobenzene-1,3-diol, which contains a hydroxyl group at the para-substrate of the benzene ring towards the thiol (analogous to the -OH group towards the sulfonamide in the compounds we have studied), and found by crystallography that on binding to the CAII isoenzyme, the hydroxyl group was unfavorably oriented towards the hydrophobic region consisting of the amino acid residues of Ile91, Phe131, Val135, Leu141, and Leu198. Meanwhile, deletion of the hydroxyl group improved the affinity of the molecule for the CA active site. Thus, targeting the hydrophilic hydroxyl group to the hydrophobic pocket of the CA active center created unfavorable conditions for the formation of interactions between these fragments. In our study, compounds with an -OEt group in place of -OH (**35**, **36**, **37**, **38**) bound more strongly than their counterparts with a hydroxyl group, which supports this hypothesis.

### 2.3. Crystal Structures

**Figure 7:**
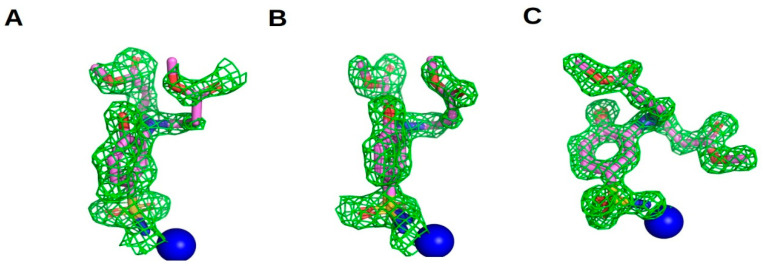
Electron density of compound **25** in CAI (**A**, **B** subunits) and CAII (**C**). The difference map is contoured at 2.0σ (CAI) and 3.0σ (CAII). The compound is colored pink, and Zn atoms are shown as blue spheres.

Based on the previously presented structure–activity relationship, even minor structural modifications in the compound series led to substantial differences in affinity toward CAs, with compound **25** showing the highest affinity gains (compared to the structurally closest compounds, **24** and **27**, Figure 5) for CAI and CAII, which guided its selection for crystallographic studies. We determined the crystal structures of the complexes of compound **25** with CAI and with CAII (PDB ID 9HWN and 9HWM, X-ray crystal structure refinement statistics and crystallization conditions presented in Supplementary Table S1). There were two protein subunits in ASU of CAI crystal and one in CAII. In all protein chains, **25** was modeled in two alternative conformations. The electron density maps of compound **25** are shown in Figure 7.

**Figure 8:**
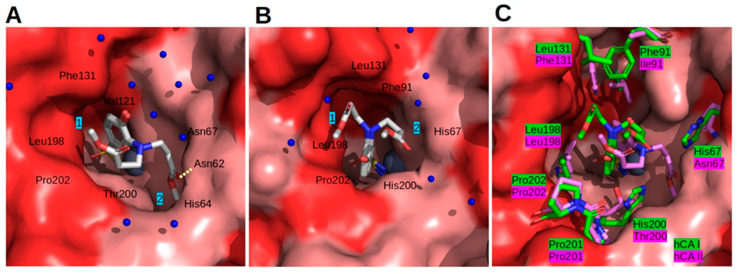
Crystallographic analysis of the interaction of compound **25** with CAI and CAII. (**A**) Compound **25** in the active site of CAII; (**B**) **25** in the active site of CAI; (**C**) comparison of **25** bound to CAI (green) and CAII (pink). Blue numeric labels 1 and 2 in (**A**,**B**) point to two alternate conformations of the compound’s flexible tail. The molecular surface belongs to the CAII model; CAs are superimposed (His94, His96, and His119 residues). The protein surface of the CA active site is colored red for hydrophobic residues (V, I, L, F, M, A, G, and P) and light pink for the residues with charged and polar side chains (R, D, N, E, Q, H, K, S, T, Y, W, and C). Side chains that differ between isoforms are shown as sticks and labelled. Zn^2+^ is shown as a grey sphere. The water molecules are presented as small blue spheres.

In CAII, compound **25** had two alternative positions, which differed only by the orientation of flexible tails (Figure 8A). The benzene ring of **25** was located at the hydrophobic part of the CAII active site (near Phe131). The flexible tail in the first alternate conformation was folded 180 degrees and fixed between the molecular surface formed by the benzene ring of compound **25** and the Pro202 chain. In the second alternate conformation, the head of the flexible tail was located deep in the pocket of the hydrophilic part of the CAII active site (under the side chains of His64 and Asn62). Additionally, an oxygen atom of the flexible tail formed a hydrogen bond with the side chain of Asn62.

The positions of the benzene rings in both subunits of CAI were very similar. In both subunits, compound **25** was modeled in two alternate conformations due to different orientations of the flexible tail. The benzenesulfonamide was tightly fixed between the side chains of Leu198 and His200. In both cases, the flexible tail was exposed to solvent due to the narrow shape of the CAI active site. In the first alternate conformation, the head of the flexible tails stuck to the hydrophobic surface. In the second alternate conformation, the head of the flexible tails was found in the hydrophilic part of the active site (Figure 8B).

The comparison of compound **25** bound to CAII and CAI is shown in Figure 8C. The main differences found in the surface of active sites comparing CAI and CAII are the following: Phe91 vs. Ile91, Leu131 vs. Phe131, His200 vs. Thr200, and His67 vs. Asn67. The side chains of His200 and His67 in CAI reduced the shape of the active site of CAI compared to CAII (Thr200 and Asn67). The shape differences of Phe131 (CAII) and Leu131 (CAI) were compensated for by differences between Ile91 (CAII) and Phe91 (CAI). Overall, the CAI active site was narrower than that of CAII.

### 2.4. Biology

**Figure 9:**
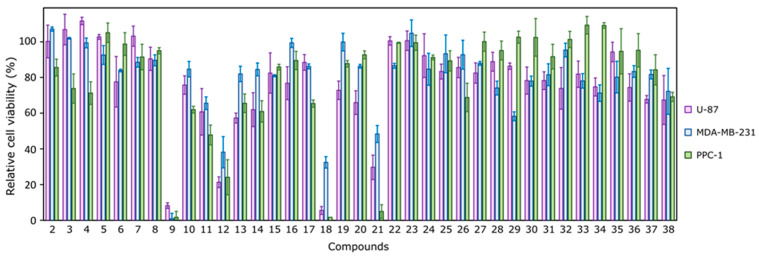
The effect of 100 μM of tested compounds on human glioblastoma (U-87), triple-negative breast cancer (MDA-MB-231), and prostate adenocarcinoma (PPC-1) cell viability after 72 h of incubation, determined by the MTT assay, *n* = 3.

We performed biological analysis of compounds **2**–**38** by evaluating their effect on cancer cell viability using the MTT assay. Generally, most compounds were not active against human glioblastoma U-87, triple-negative breast cancer MDA-MB-231, or prostate adenocarcinoma PPC-1 cell lines and reduced cell viability only up to about 50% at 100 μM concentration (Figure 9). This could be explained by the aggressiveness and resistance of the selected cell lines [4,5,6]. Only four compounds (**9**, **12**, **18**, and **21**) reduced the viability of at least one of the tested cell lines up to <20% (Figure 9). These compounds showed a very distinct CA binding profile (Table 1). Compounds **12** and **18** were characterized by relatively low *K*_d_s towards most CAs, while compound **9** showed a modest affinity, and compound **21** did not bind any of the tested CAs (*K*_d_ ≥ 200 μM). Interestingly, compound **14**, which bound most CAs with relatively low *K*_d_, did not show high cytotoxicity against either of the tested cancer cell lines.

Thus, for further biological experiments, we selected the four most active candidates to establish their half-maximal effective concentrations (EC_50_ values) and explore their effects in tumor spheroids—a 3D cell model with hypoxia—to investigate the possible relationship with their inhibitory effect on different CAs and anticancer activity.

**Figure 10:**
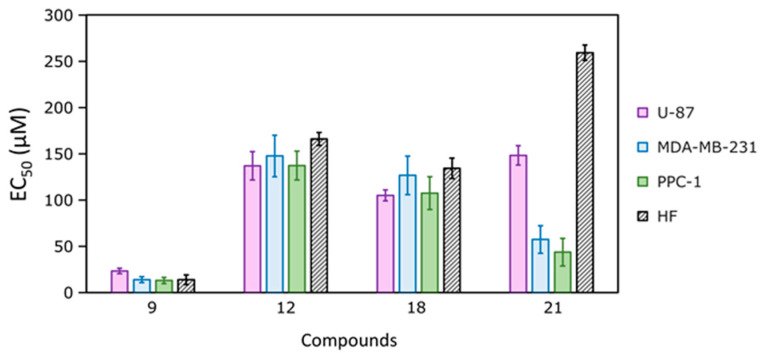
The half-maximal effective concentrations (EC_50_) of the most active compounds **9**, **12**, **18**, and **21**, determined by the MTT assay after 72 h of incubation with human glioblastoma (U-87), triple-negative breast cancer (MDA-MB-231), and prostate adenocarcinoma (PPC-1) cell lines and human foreskin fibroblasts (HFs). EC_50_ values were calculated using the Hill equation, *n* = 3.

After the dose–effect correlation studies in cell lines, the EC_50_ values were calculated, which indicate the concentration at which tested compounds reduced the cell viability by 50% (Figure 10 and Table 2). The most cytotoxic was compound **9**, especially against triple-negative breast cancer and prostate adenocarcinoma cell lines. However, it was not selective against cancer cells compared to fibroblasts, as the EC_50_ values were similar, and even almost twice as low in fibroblasts compared to the glioblastoma cell line.

The activity of compounds **12** and **18** was comparable towards cancer cells and fibroblasts, and there was no statistically significant difference between their EC_50_ values against separate cancer cell lines. Compound **21** was less active against the glioblastoma cell line but more active against the breast cancer and prostate cell lines and also showed 1.5–6 times higher selectivity for cancer cells over fibroblasts, which could make it a good candidate for more detailed experiments. As was discussed earlier, compound **21** did not bind to CAs and possibly acts through a different mechanism of action, which still needs to be discovered. It should be noted that, though the cytotoxicity was not very high, generally all four compounds were more active against the triple-negative breast cancer cell line MDA-MB-231 compared to CA-IX inhibitor U-104, and more active against all cell lines than the non-selective CA inhibitor acetazolamide (Table 2).

Taking into account the high activity of compound **9** and selectivity of compound **21** towards cancer cells over fibroblasts, those two candidates were tested in cancer cell three-dimensional (3D) cultures. The spheroids were formed from cancer cells and fibroblasts at a ratio of 1:1 to better mimic the tumor microenvironment [40]. In addition, fibroblasts served as a support for cancer cells and helped to keep them in a 3D configuration for 10 days (the duration of the experiments). From our previous experiments, we noticed that MDA-MB-231 cells alone did not form spheroids, or after forming spheroids, they tended to disintegrate in a few days.

**Figure 11:**
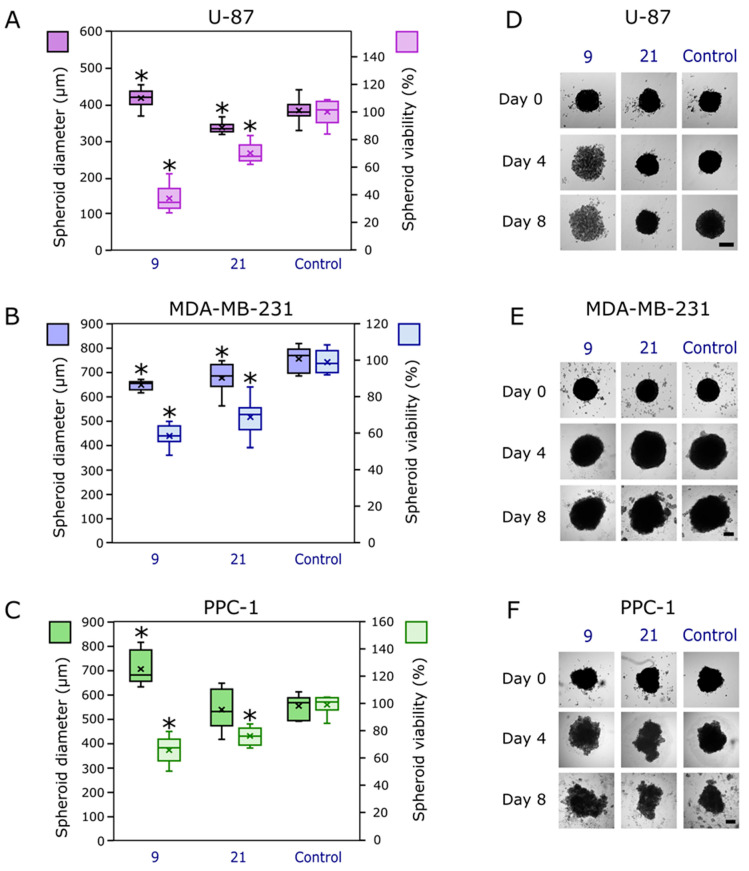
The effect of the most active compounds, **9** and **21**, at 10 mM concentration in cancer cell spheroids. (**A**) Human glioblastoma U-87 spheroid size (brighter purple color) and viability of cells in spheroids (lighter purple color) at the end of the experiment. (**B**) Human triple-negative breast cancer MDA-MB-231 spheroid size (brighter blue color) and viability of cells in spheroids (lighter blue color) at the end of the experiment. (**C**) Human prostate adenocarcinoma PPC-1 spheroid size (brighter green color) and viability of cells in spheroids (lighter green color) at the end of the experiment. (**D**) Photos of U-87 cell spheroids at the beginning (Day 0), in the middle (Day 4) and at the end (Day 8) of the experiment (after incubation with 10 µM of compounds). (**E**) Photos of MDA-MB-231 cell spheroids at the beginning (Day 0), in the middle (Day 4), and at the end (Day 8) of the experiment (after incubation with 10 µM of compounds). (**F**) Photos of PPC-1 cell spheroids at the beginning (Day 0), in the middle (Day 4), and at the end (Day 8) of the experiment (after incubation with 10 µM of compounds). Asterisks (*) indicate *p* < 0.05 compared to the control (untreated spheroids); crosses (×) indicate means; inner dashes indicate medians; whiskers indicate maximum and minimum values. Scale bar indicates 200 µm.

Compound **21** reduced the growth of glioblastoma and breast cancer spheroids (Figure 11A,B,D,E), with no statistically significant effect on prostate cancer spheroids (Figure 11C,F). However, compound **9** showed a different effect in 3D cultures, as in its presence, the glioblastoma and prostate cancer spheroids at the end of the experiment became more diffuse, and some cells were detached from the cultures (Figure 11D,F). On the contrary, breast cancer MDA-MB-231 spheroids became smaller compared to the control (Figure 11E). It could be hypothesized that this effect is related to the inhibition of CAIX, which is highly expressed in MDA-MB-231 cells under hypoxia, while this phenomenon is not relevant for glioblastoma and prostate cells, as discussed earlier. However, in our previous experiments, we noticed that the spheroid size does not always correlate with cell viability, as some compounds may affect the cell–cell interaction and the compactness of the structure [41], so at the end of the experiment, we measured the viability of cell spheroids. It was determined that compound **9** exhibited the strongest effect on cell viability reduction of all three types of spheroids (Figure 11A–C). Overall, its effect on spheroids’ integrity could be explained by its mechanism of action related to the inhibition of carbonic anhydrases, especially CA IX, which is known to be related to cell–cell adhesion [42]. The activity of compound **21** most probably was achieved through a different mechanism of action, as it did not bind to CAs (Table 1). The obtained promising results in 3D cultures suggest that it is worth exploring the pharmacology of compound **21**, considering its selectivity towards the tested cancer cell lines.

Overall, the obtained results suggest that the strongest binding to CAs is not the only or most important factor to achieve a higher effect in cancer cell 2D and 3D cultures. We hypothesize that other factors could also contribute to compound biological activity, such as oxygen concentration, cell permeability, off-target effects, etc. It is known that sulfonamides can bind not only to CAs but also to other targets, which may contribute to their polypharmacological [43] or off-target effects [44]. Additionally, it cannot be ruled out that sulfonamides could bind to other zinc-containing enzymes, of which there are over 200 identified [45]. Moreover, the selected most active sulfonamides contain different chemical fragments apart from the sulfonamide group, and they could also contribute to additional mechanisms. For example, compound **9**, which was the most active in the cancer cell models, contains a nitro-thiophene group, which is known to play a role in intercalating DNA [41]. This way, several possible mechanisms of action could make this compound more potent in biological assays. Also, though compound **21** is a secondary sulfonamide and did not bind to CAs, its biological activity in cancer cells could be due to another mechanism, as some authors revealed that secondary sulfonamides can bind to another zinc-containing enzyme—histone deacetylase [46].

Overall, our results are not very surprising, as some earlier research conducted on sulfonamides binding to CAII and biological activity revealed the unpredictable behavior of some drugs, possibly as a consequence of their activity toward multiple targets [47]. Considering the complicated mechanism of action of sulfonamides, we suggest that further thorough analysis is needed to come to clearer conclusions on the correlations between distinct binding to CAs and biological activity in clearly defined in vitro tumor models.

## 3. Materials and Methods

### 3.1. Chemistry

Reagents and solvents were obtained from Sigma-Aldrich (St. Louis, MO, USA) and used without further purification. The reaction course and purity of the synthesized compounds were monitored via TLC using aluminum plates precoated with silica gel with 60 F254 nm (Merck KGaA, Darmstadt, Germany). Melting points were determined with a Barnstead Electrothermal MEL-TEMP^®^ 1101D (Electrothermal, Staffordshire, UK) and were uncorrected. NMR spectra were recorded on a Bruker BioSpin GmbH (^1^H 400 MHz, ^13^C 101 MHz) spectrometer (Bruker BioSpin GmbH, Rheinstetten, Germany). Chemical shifts were reported in (δ) ppm relative to tetramethylsilane (TMS) with the residual solvent as an internal reference (DMSO-*d*_6_, δ = 2.50 ppm for ^1^H and d = 39.5 ppm for ^13^C). Data were reported as follows: chemical shift, multiplicity, coupling constant (Hz), integration, and assignment. IR spectra (ν, cm^−1^) were recorded on a PERKIN ELMER Spectrum Bx FT-IR (Perkin–Elmer Inc., Waltham, MA, USA) using KBr pellets. Mass spectra were obtained on a Waters SQ Detector 2 mass spectrometer (Waters, Milford, MA, USA) with ESI ionization. Elemental analyses (C, H, N) were conducted using the Elemental Analyzer CE-440 (Exeter Analytical, Inc., Chelmsford, MA, USA); their results were found to be in good agreement (±0.3%) with the calculated values.

**General procedure of the preparation of Schiff bases 2**–**9**

A solution of 3-amino-4-hydroxybenzenesulfonamide (**1**) (1.88 g, 10 mmol) and the corresponding aldehyde (14 mmol) in propan-2-ol (30 mL) was heated at reflux for 1 h, then cooled, and the formed precipitated was filtered off, washed with propan-2-ol and diethyl ether, and recrystallized from a mixture of acetone: hexane 1:1.

**3-(Benzylideneamino)-4-hydroxybenzenesulfonamide (2)**

White solid, yield 2.63 g, 95%, mp 210–212 °C.

^1^H NMR (400 MHz, DMSO-*d*_6_) δ 9.95 (s, 1H, OH), 8.72 (s, 1H, N=CH), 8.05 (d, *J* = 6.7 Hz, 2H, H_Ar_), 7.60 (s, 1H, H_Ar_), 7.58–7.50 (m, 4H, H_Ar_), 7.17 (s, 2H, NH_2_), 7.03 (d, *J* = 8.4 Hz, 1H, H_Ar_) ppm.

^13^C NMR (101 MHz, DMSO-*d*_6_) δ 161.33, 153.70, 138.17, 135.97, 135.13, 131.74, 129.08, 128.79, 124.85, 117.48, 115.94 (C_Ar_, N=CH) ppm.

IR (KBr): n 3376 (O-H); 3345 (N-H); 1626 (C=N); 1303 (C-N); 1165 (S=O) cm^−1^.

Anal. Calcd for C_13_H_12_N_2_O_3_S, %: C 56.51; H 4.38; N 10.14; Found, %: C 56.48; H 4.35; N 10.17.

**3-((4-Fluorobenzylidene)amino)-4-hydroxybenzenesulfonamide (3)**

White solid, yield 1.67 g, 57%, mp 252–254 °C.

^1^H NMR (400 MHz, DMSO-*d*_6_) δ 9.93 (s, 1H, OH), 8.72 (s, 1H, N=CH), 8.13 (t*, J* = 8.5 Hz, 2H, H_Ar_), 7.60 (s, 1H, H_Ar_), 7.55 (d, *J* = 8.4 Hz, 1H, H_Ar_), 7.37 (t, *J* = 8.5 Hz, 2H, H_Ar_), 7.16 (s, 2H, NH_2_), 7.03 (d, *J* = 8.4 Hz, 1H, H_Ar_) ppm.

^13^C NMR (101 MHz, DMSO-*d*_6_) δ 164.19 (d, *J* = 249.7 Hz, CF), 159.90, 153.79, 137.86, 135.12, 132.68, 131.49 (d, *J* = 9.0 Hz, CF), 124.93, 117.31 (C_Ar_, N=CH), 115.87 (d, *J* = 21.8 Hz, CF) ppm.

IR (KBr): n 3381 (O-H); 3331 (N-H); 1627 (C=N); 1305 (C-N); 1220 (C-F); 1169 (S=O) cm^−1^.

Anal. Calcd for C_13_H_11_FN_2_O_3_S, %: C 53.06; H 3.77; N 9.52. Found, %: C 53.10; H 3.73; N 9.51.

**3-((4-Chlorobenzylidene)amino)-4-hydroxybenzenesulfonamide (4)**

White solid, yield 2.84 g, 91%, mp 239–241 °C.

^1^H NMR (400 MHz, DMSO-*d*_6_) δ 9.98 (s, 1H, OH), 8.74 (s, 1H, N=CH), 8.08 (d, *J* = 8.2 Hz, H_Ar_), 7.62 (s, 2H, H_Ar_), 7.60 (s, 1H, H_Ar_), 7.56 (d, *J* = 8.5 Hz, 1H, H_Ar_), 7.17 (s, 2H, NH_2_), 7.03 (d, *J* = 8.5 Hz, 1H, H_Ar_) ppm.

^13^C NMR (101 MHz, DMSO-*d*_6_) δ 159.93, 153.86, 137.66, 136.31, 135.14, 134.86, 130.72, 128.90, 125.14, 117.38, 116.05 (C_Ar_, N=CH) ppm.

IR (KBr): n 3400 (O-H); 3329 (N-H); 1625 (C=N); 1322 (C-N); 1163 (S=O); 689 (C-Cl) cm^−1^.

Anal. Calcd for C_13_H_11_ClN_2_O_3_S, %: C 50.25; H 3.57; N 9.01. Found, %: C 50.28; H 3.59; N 9.03.

**4-Hydroxy-3-((4-methoxybenzylidene)amino)benzenesulfonamide (5)**

White solid, yield 2.53 g, 83%, mp 178–180 °C.

^1^H NMR (400 MHz, DMSO-*d*_6_) δ 9.82 (s, 1H, OH), 8.62 (s, 1H, N=CH), 8.00 (d, *J* = 8.2 Hz, 2H, H_Ar_), 7.57 (s, 1H, H_Ar_), 7.52 (d, *J* = 8.3 Hz, 2H, H_Ar_), 7.15 (s, 2H, NH_2_), 7.08 (d, *J* = 8.2 Hz, 1H, H_Ar_), 7.01 (d, *J* = 8.3 Hz, 1H, H_Ar_), 3.85 (s, 3H, CH_3_) ppm.

^13^C NMR (101 MHz, DMSO-*d*_6_) δ 162.16, 160.38, 153.72, 138.43, 135.10, 130.98, 128.89, 124.42, 117.13, 115.73, 114.23 (C_Ar_, N=CH), 55.46 (OCH_3_) ppm.

IR (KBr): n 3359 (O-H); 3277 (N-H); 1623 (C=N); 1323 (C-N); 1254 (C-O); 1167 (S=O) cm^−1^.

Anal. Calcd for C_14_H_14_N_2_O_4_S, %: C 54.89; H 4.61; N 9.14. Found, %: C 54.92; H 4.64; N 9.16.

**4-Hydroxy-3-((naphthalen-1-ylmethylene)amino)benzenesulfonamide (6)**

White solid, yield 2.73 g, 77%, mp 238–240 °C.

^1^H NMR (400 MHz, DMSO-*d*_6_) δ 10.12 (s, 1H, OH), 9.29 (s, 1H, N=CH), 9.16 (d, *J* = 8.5 Hz, 1H, H_Ar_), 8.30 (d, *J* = 7.1 Hz, 1H, H_Ar_), 8.15 (d, *J* = 8.1 Hz, 1H, H_Ar_), 8.06 (d, *J* = 8.0 Hz, 1H, H_Ar_), 7.75–7.59 (m, 4H, H_Ar_), 7.57 (d, *J* = 8.4 Hz, 1H, H_Ar_), 7.19 (s, 2H, NH_2_), 7.07 (d, *J* = 8.4 Hz, 1H, H_Ar_) ppm.

^13^C NMR (101 MHz, DMSO-*d*_6_) δ 161.26, 153.45, 139.26, 135.18, 133.50, 132.20, 130.42, 128.75, 127.64, 126.39, 125.50, 124.63, 124.39, 117.94, 115.88 (C_Ar_, N=CH) ppm.

IR (KBr): n 3397 (O-H); 3322 (N-H); 1628 (C=N); 1311 (C-N); 1163 (S=O) cm^−1^.

Anal. Calcd for C_17_H_14_N_2_O_4_S, %: C 62.56; H 4.32; N 8.58. Found, %: C 62.52; H 4.35; N 8.55.

**4-Hydroxy-3-((naphthalen-2-ylmethylene)amino)benzenesulfonamide (7)**

White solid, yield 2.8 g, 79%, mp 208–210 °C.

^1^H NMR (400 MHz, DMSO-*d*_6_) δ 10.02 (s, 1H, OH), 8.89 (s, 1H, N=CH), 8.48 (s, 1H, H_Ar_), 8.29 (d, *J* = 8.5 Hz, 1H, H_Ar_), 8.10–7.97 (m, 3H, H_Ar_), 7.71–7.54 (m, 4H, H_Ar_), 7.19 (s, 2H, NH_2_), 7.06 (d, *J* = 8.2 Hz, 1H, H_Ar_) ppm.

^13^C NMR (101 MHz, DMSO-*d*_6_) δ 161.58, 154.38, 138.55, 135.58, 135.09, 134.23, 133.12, 132.21, 129.29, 128.86, 128.35, 127.33, 125.40, 124.43, 118.03, 116.49 (C_Ar_, N=CH) ppm.

IR (KBr) n 3344 (O-H); 3252 (N-H); 1619 (C=N); 1323 (C-N); 1164 (S=O) cm^−1^.

Anal. Calcd for C_17_H_14_N_2_O_4_S, %: C 62.56; H 4.32; N 8.58. Found, %: C 62.59; H 4.35; N 8.55.

**4-Hydroxy-3-((thiophen-2-ylmethylene)amino)benzenesulfonamide (8)**

Yellowish solid, yield 2.31 g, 82%, mp 180–182 °C.

^1^H NMR (400 MHz, DMSO-*d*_6_) δ 10.10 (s, 1H, OH), 8.85 (s, 1H, N=CH), 7.85 (d, *J* = 4.8 Hz, 1H, H_Ar_), 7.75 (d, *J* = 3.0 Hz, 1H, H_Ar_), 7.52 (s, 1H, H_Ar_), 7.49 (s, 1H, H_Ar_), 7.23 (t, *J* = 4.1 Hz, 1H, H_Ar_), 7.15 (s, 2H, NH_2_), 7.01 (d, *J* = 8.3 Hz, 1H, H_Ar_) ppm.

^13^C NMR (101 MHz, DMSO-*d*_6_) δ 155.17, 153.36, 142.43, 138.01, 135.06, 133.94, 131.69, 128.27, 124.48, 118.82, 115.95 (C_Ar_, N=CH) ppm.

IR (KBr) n 3392 (O-H); 3337 (N-H); 1614 (C=N); 1303 (C-N) 1166 (S=O) cm^−1^.

Anal. Calcd for C_11_H_10_N_2_O_3_S_2_, %: C 46.80; H 3.57; N 9.92. Found, %: C 46.85; H 3.56; N 9.94.

**4-Hydroxy-3-(((5-nitrothiophen-2-yl)methylene)amino)benzenesulfonamide (9)**

Yellow solid, yield 2.97 g, 91%, mp 257–259 °C.

^1^H NMR (400 MHz, DMSO-*d*_6_) δ 10.43 (s, 1H, OH), 9.04 (s, 1H, N=CH), 8.19 (d, *J* = 4.1 Hz, 1H, H_Ar_), 7.80 (d, *J* = 4.2 Hz, 1H, H_Ar_), 7.67 (s, 1H, H_Ar_), 7.59 (d, *J* = 8.5 Hz, 1H, H_Ar_), 7.20 (s, 2H, NH_2_), 7.07 (d, *J* = 8.5 Hz, 1H, H_Ar_) ppm.

^13^C NMR (101 MHz, DMSO-*d*_6_) δ 154.38, 154.07, 152.89, 148.73, 135.98, 135.22, 132.20, 130.34, 126.09, 119.50, 116.63 (C_Ar_, N=CH) ppm.

IR (KBr) n 3361 (O-H); 3330 (N-H); 1611 (C=N); 1500 (NO_2_); 1343 (C-N); 1149 (S=O) cm^−1^.

Anal. Calcd for C_11_H_9_N_3_O_5_S_2_, %: C 40.36; H 2.77; N 12.84. Found, %: C 40.39; H 2.74; N 12.82.

**General procedure for the preparation of aminoketones 10–14**

A solution of 3-amino-4-hydroxybenzenesulfonamide **1** (1.88 g, 10 mmol) and the corresponding bromoacetophenone (11 mmol) in a mixture of water and propan-2-ol (1:1, 100 mL) was heated at reflux for 4 h, then cooled, and the formed precipitated was filtered off, washed with propan-2-ol and diethyl ether, and recrystallized from propan-2-ol (**10**, **13**, **14**) or from 1,4-dioxane (**11**, **12**).

**4-Hydroxy-3-((2-oxo-2-phenylethyl)amino)benzenesulfonamide (10).**

Resynthesis was carried out according to Ref. [32], and the spectral data are consistent with the original ones.

White solid, yield 2.51 g, 82%, mp. 218–220 °C.

**3-((2-(2,4-Difluorophenyl)-2-oxoethyl)amino)-4-hydroxybenzenesulfonamide (11)**

White solid, yield 1.13 g, 33%, mp 207–209 °C.

^1^H NMR (400 MHz, DMSO-*d*_6_) δ 8.05–7.97 (m, 1H, OH), 7.70–7.63 (m, 2H, H_Ar_), 7.15–7.09 (m, 2H, H_Ar_), 7.07 (s, 2H, NH_2_), 7.18–6.96 (m, 2H, H_Ar_, NH_2_), 4.92, 4.78 (2s, 1H, NH), 3.70, 3.54 (2d, *J* = 12.0 Hz, 2H, CH_2_) ppm.

^13^C NMR (101 MHz, DMSO-*d*_6_) δ 192.77, 192.70 (C=O), 165.53 (d, *J* = 242.1 Hz, CF), 162.56 (d, *J* = 234.6 Hz, CF), 159.89 (d, *J* = 240.2 Hz, CF), 144.10, 137.25, 133.89, 132.57 (d, *J* = 10.9 Hz, CF),, 122.52 (d, *J* = 12.5 Hz, CF), 116.01, 115.36 (C_Ar_), 112.67 (d, *J* = 23.8 Hz, CF), 111.04 (d, *J* = 24.4 Hz, CF), 105.11 (t, *J* = 26.4 Hz, CF), 54.20 (CH_2_) ppm.

IR (KBr) n 3365 (O-H); 3264 (N-H); 1666 (C=O); 1248; 1224; 1040 (N-C, C-F); 1150 (S=O) cm^−1^.

Anal. Calcd for C_14_H_12_F_2_N_2_O_4_S, %: C 49.12; H 3.53; N 8.18. Found, %: C 49.16; H 3.51; N 8.20.

**4-Hydroxy-3-((2-(4-chlorophenyl)-2-oxoethyl)amino)benzenesulfonamide (12)**

Resynthesis was carried out according to Ref. [32], and the spectral data are consistent with the original ones.

**4-Hydroxy-3-((2-(4-hydroxyphenyl)-2-oxoethyl)amino)benzenesulfonamide (13)**

White solid, yield 1.80 g, 56%, mp 217–219 °C.

^1^H NMR (400 MHz, DMSO-*d*_6_) δ 10.48 (s, 1H, OH), 10.31 (s, 1H, OH), 7.97 (d, *J* = 8.1 Hz, 2H, H_Ar_), 6.98 (d, *J* = 7.7 Hz, 4H, NH_2_, H_Ar_), 6.91 (d, *J* = 8.1 Hz, 2H, H_Ar_), 6.78 (d, *J* = 7.8 Hz, 1H, H_Ar_), 5.43 (s, 1H, NH), 4.62 (d, *J* = 4.2 Hz, 2H, CH_2_) ppm.

^13^C NMR (101 MHz, DMSO-*d*_6_) δ 193.63 (C=O), 162.59, 147.05, 136.75, 135.26, 130.47, 126.34, 115.45, 114.51, 112.14, 107.07 (C_Ar_), 49.06 (CH_2_) ppm.

IR (KBr) n 3462; 3421 (O-H); 3284 (N-H); 1662 (C=O); 1251; 1082 (N-C); 1146 (S=O) cm^−1^.

Anal. Calcd for C_14_H_14_N_2_O_5_S, %: C 52.17; H 4.38; N 8.69. Found, %: C 52.20; H 4.38; N 8.66.

**4-Hydroxy-3-((2-(naphthalen-1-yl)-2-oxoethyl)amino)benzenesulfonamide (14)**

White solid, yield 2.73 g, 77%, mp 220–222 °C.

^1^H NMR (400 MHz, DMSO-*d*_6_) δ 10.36 (s, 1H, OH), 8.83 (s, 1H, H_Ar_), 8.19 (d, *J* = 7.9 Hz, 1H, H_Ar_), 8.08 (s, 2H, H_Ar_), 8.03 (d, *J* = 7.9 Hz, 1H, H_Ar_), 7.73–7.64 (m, 2H, H_Ar_), 7.06 (s, 1H, H_Ar_), 7.01 (s, 1H, H_Ar_), 6.99 (s, 2H, NH_2_), 6.81 (d, *J* = 8.1 Hz, 1H, H_Ar_), 5.51 (br. s, 1H, NH), 4.89 (s, 2H, CH_2_) ppm.

^13^C NMR (101 MHz, DMSO-*d*_6_) δ 195.77 (C=O), 147.07, 136.69, 135.33, 135.28, 132.20, 132.16, 129.77, 129.64, 128.89, 128.49, 127.78, 127.13, 123.43, 114.63, 112.20, 107.09 (C_Ar_), 49.72 (CH_2_) ppm.

IR (KBr) n 3420 (O-H); 3347 (N-H); 1674 (C=O); 1251; 1079 (N-C); 1145 (S=O) cm^−1^.

Anal. Calcd. for C_18_H_16_ClN_2_O_4_S, %: C 60.66; H 4.53; N 7.86. Found, %: C 60.62; H 4.57; N 7.88.

**General procedure for the synthesis of hydroxylamine-containing compounds 15-17**

To a solution of the corresponding compounds **10**, **12**, and **13** (2 mmol) in methanol (20 mL), hydroxylamine hydrochloride (0.278 g, 4 mmol) and sodium acetate trihydrate (0.54 g, 4 mmol) were added and the mixture was refluxed for 4 h. After completion of the reaction, methanol was evaporated under reduced pressure, and the resulting solid was recrystallized from water.

**4-Hydroxy-3-((2-(hydroxyimino)-2-phenylethyl)amino)benzenesulfonamide (15)**

White solid, yield 0.27 g, 42%, mp 160–162 °C.

^1^H NMR (400 MHz, DMSO-*d*_6_) δ *Z/E* mixture, 80:20, 11.65, 11.07 (2s, 1H, OH), 10.18, 10.10 (2s, 1H, OH), 7.65, 7.55 (2d, *J* = 6.5 Hz, 2H, H_Ar_), 7.43–7.30 (m, 3H, H_Ar_), 7.08 (s, 1H, H_Ar_), 7.00–6.88 (m, 3H, H_Ar_, NH_2_), 6.73, 6.67 (2d, *J* = 8.0 Hz, 1H, H_Ar_), 5.30, 5.14 (2t, *J* = 5.5 Hz, 1H, NH), 4.44, 4.17 (2d, *J* = 5.4 Hz, 2H, CH_2_) ppm.

^13^C NMR (101 MHz, DMSO-*d*_6_) δ 154.97, 146.88, 136.77, 135.31, 134.91, 128.65, 128.21, 128.04, 126.60, 114.60, 112.17, 106.66 (C_Ar_, C=N), 37.12 (CH_2_) ppm.

IR (KBr) n 3476; 3406 (O-H); 3255 (N-H) 1646 (C=N); 1282; 1075 (N-C); 1157 (S=O) cm^−1^.

Anal. Calcd for C_14_H_15_N_3_O_4_S, %: C 52.33; H 4.71; N 13.08. Found, %: C 52.30; H 4.68; N 13.07.

**4-Hydroxy-3-((2-(hydroxyimino)-2-(4-hydroxyphenyl)ethyl)amino)benzenesulfonamide (16)**

White solid, yield 0.26 g, 38%, mp 128–130 °C.

^1^H NMR (400 MHz, DMSO-*d*_6_) δ *Z/E* mixture, 80:20, 11.34, 10.97 (2s, 1H, OH), 10.09 (s, 1H, OH), 9.64 (s, 1H, OH), 7.54, 7.48 (2d, *J* = 8.1 Hz, 2H, H_Ar_), 7.09, 7.06 (2s, 1H, H_Ar_), 6.94 (s, 2H, NH_2_), 6.92 (s, 1H, H_Ar_), 6.79, 6.73 (2d, *J* = 8.1 Hz, 2H, H_Ar_), 6.68 (d, *J* = 8.1 Hz, 1H, H_Ar_), 5.26, 5.01 (2t, *J* = 5.8 Hz, 1H, NH), 4.34, 4.14 (2d, *J* = 5.7 Hz, 2H, CH_2_) ppm.

^13^C NMR (101 MHz, DMSO-*d*_6_) δ 158.06, 154.41, 146.94, 136.88, 135.30, 130.12, 127.89, 125.67, 115.09, 114.74, 114.59, 112.16, 106.76 (C_Ar_, C=N), 37.12 (CH_2_) ppm.

IR (KBr) n 3193 (O-H, N-H); 1647 (C=N); 1280; 1071(N-C); 1159 (S=O) cm^−1^.

Anal. Calcd for C_14_H_15_N_3_O_5_S, %: C 49.85; H 4.48; N 12.46. Found, %: C 49.82; H 4.50; N 12.50.

**4-Hydroxy-3-((2-(hydroxyimino)-2-(4-chlorophenyl)ethyl)amino)benzenesulfonamide (17)**

White solid, yield 0.35 g, 49%, mp 137–139 °C.

^1^H NMR (400 MHz, DMSO-*d*_6_) δ *Z/E* mixture, 80:20, 11.75, 11.21 (2s, 1H, OH), 10.10 (br. s, 1H, OH), 7.66, 7.58 (2d, *J* = 8.3 Hz, 2H, H_Ar_), 7.47, 7.39 (2d, *J* = 8.3 Hz, 2H, H_Ar_), 7.06, 7.02 (2s, 1H, H_Ar_), 6.92 (s, 2H, NH_2_), 6.90 (s, 1H, H_Ar_), 6.71, 6.65 (2d, *J* = 8.2 Hz, 1H, H_Ar_), 5.32, 5.25 (2t, *J* = 5.8 Hz, 1H, NH), 4.48, 4.18 (2d, *J* = 5.6 Hz, 2H, CH_2_) ppm.

^13^C NMR (101 MHz, DMSO-*d*_6_) δ 154.34, 146.97, 136.63, 135.14, 133.74, 133.23, 130.24, 128.44, 128.13, 114.61, 112.13, 106.51 (C_Ar_, C=N), 36.95 (CH_2_) ppm.

IR (KBr) n 3406–3240 (O-H, N-H); 1606 (C=N); 1278; 1095 (N-C); 1150 (S=O); 835 (C-Cl) cm^−1^.

Anal. Calcd for C_14_H_14_ClN_3_O_4_S, %: C 47.26; H 3.97; N 11.81. Found, %: C 47.30; H 4.00; N 11.76.

**3,3′-((Hydrazine-1,2-diylidenebis(2-phenylethan-1-yl-2-ylidene))bis(azanediyl))bis(4-hydroxybenzenesulfonamide) (18)**

To a solution of compound **10** (0.613 g, 2 mmol) in methanol (15 mL), hydrazine monohydrate (0.055 g, 1.1 mmol) was added and the mixture was refluxed for 2 h, then cooled, and the formed precipitate was filtered off and washed with water. Product **10** was purified by dissolving it in aqueous 2.5% sodium hydroxide, filtering, and acidifying the filtrate with 30% acetic acid to pH 6–7. The purification process was repeated twice.

White solid, yield 0.56 g, 92%, mp 185–189 °C.

^1^H NMR (400 MHz, DMSO-*d*_6_) δ 8.09 (d, *J* = 6.9 Hz, 4H, H_Ar_), 7.72-7.55 (m, 6H, H_Ar_), 7.01–6.54 (m, 12H, NH_2_, H_Ar_, 2OH), 5.48 (2s, 2H, 2NH), 4.84 (s, 2H, CH_2_), 4.74 (d, *J* = 4.2 Hz, 2H, CH_2_) ppm;

^13^C NMR (101 MHz, DMSO-*d*_6_) δ 195.88 (C=N), 148.19, 138.35, 136.83, 134.92, 134.40, 133.76, 129.04, 128.89, 128.12, 127.86, 127.52, 127.08, 125.18, 114.77, 112.09, 106.89 (C_Ar_), 49.77 (2CH_2_) ppm;

IR (KBr) n 3429 (O-H); 3368 (N-H); 1676 (C=N); 1223; 1085 (N-C); 1162 (S=O) cm^−1^.

MS (ESI), *m/z* (%): [M + 2H]^+^ = 610.96 (100).

Anal. Calcd for C_28_H_28_N_6_O_6_S_2_, %: C 55.25; H 4.64; N 13.81. Found, %: C 55.29; H 4.63; N 13.85.

**General procedure for the synthesis of oxoimidazoles 19, 20**

A solution of the corresponding compounds **10** or **13** (5 mmol) and carbamide (1.2 g, 20 mmol) in glacial acetic acid (15 mL) was refluxed for 24 h, then cooled and diluted with water (50 mL). The formed precipitation was filtered off, washed with water, and purified by dissolving it in aqueous 2.5% sodium hydroxide, filtering, and acidifying the filtrate with 30% acetic acid to pH 6-7. The purification process was repeated twice.

**4-Hydroxy-3-(2-oxo-4-phenyl-2,3-dihydro-1*H*-imidazol-1-yl)benzenesulfonamide (19).** Resynthesis was carried out based on Ref. [32], and the spectral data are consistent with the original ones.

White solid, yield 1.25 g, 75%, mp 271–273 °C.

**4-Hydroxy-3-(4-(4-hydroxyphenyl)-2-oxo-2,3-dihydro-1*H*-imidazol-1-yl)benzenesulfonamide (20)**

White solid, yield 0.55 g, 32%, mp 289–290 °C.

^1^H NMR (400 MHz, DMSO-*d*_6_) δ 11.00, 10.84, 9.57 (3s, 3H, NH, 2OH), 7.83 (s, 1H, NCH), 7.65 (d, *J* = 8.6 Hz, 1H, H_Ar_), 7.42 (d, *J* = 8.2 Hz, 2H, H_Ar_), 7.25 (s, 2H, NH_2_), 7.12 (d, *J* = 8.6 Hz, 1H, H_Ar_), 7.03 (s, 1H, H_Ar_), 6.77 (d, *J* = 8.2 Hz, 2H, H_Ar_) ppm.

^13^C NMR (101 MHz, DMSO-*d*_6_) δ 156.72, 154.65, 152.89, 135.03, 126.15, 125.23, 124.91, 124.22, 122.52, 120.24, 117.30, 115.64, 107.28 (C_Ar_, C=O) ppm.

IR (KBr) n 3600-3190 (O-H, N-H); 1656 (C= 270; 1219; 1085 (N-C); 1165 (S=O) cm^−1^.

Anal. Calcd for C_15_H_13_N_3_O_5_S, %: C 51.87; H 3.77; N 12.10. Found, %: C 51.96; H 3.79; N 12.04.

**4-Ethoxy-*N,N*-diethyl-3-(3-ethyl-2-oxo-4-phenyl-2,3-dihydro-1*H*-imidazol-1-yl)benzenesulfonamide (21).** To a solution of compound **19** (1.04 g, 3 mmol) in DMSO (10 mL), ground potassium hydroxide (0.25 g, 4.5 mmol) and potassium carbonate (2.1 g, 15 mmol) were added slowly, and then ethyl iodide (3.12 g, 20 mmol) was added dropwise over 10 min. The reaction mixture was heated at reflux for 24 h, then ethyl iodide together with DMSO was evaporated under reduced pressure. The obtained amorphous mass was purified by column chromatography. Eluent: acetone/hexane, 1:1.

White solid, yield 0.9 g, 41%, mp 55–57 °C.

^1^H NMR (400 MHz, DMSO-*d*_6_) δ 7.88 (s, 1H, NCH), 7.74 (d, *J* = 8.6 Hz, 1H, H_Ar_), 7.54–7.39 (m, 5H, H_Ar_), 7.36 (d, *J* = 8.8 Hz, 1H, H_Ar_), 6.94 (s, 1H, H_Ar_), 4.19 (q, *J* = 6.9 Hz, 2H, CH_2_), 3.76 (q, *J* = 6.9 Hz, 2H, CH_2_), 3.16 (q, *J* = 7.0 Hz, 4H, 2CH_2_), 1.34 (t, *J* = 6.9 Hz, 3H, CH_3_), 1.19–0.96 (m, 9H, CH_3_) ppm;

^13^C NMR (101 MHz, DMSO-*d*_6_) δ 155.54, 151.93, 130.99, 129.38, 129.00, 128.10, 127.51, 127.29, 125.81, 125.28, 123.42, 113.72, 110.07 (C_Ar_, C=O), 64.73 (CH_2_), 41.93, 36.46, 30.69 (3CH_2_), 14.41, 14.34, 14.18 (4CH_3_) ppm;

IR (KBr) n 1693 (C=O); 1331; 1270; 1251; 1219; 1200; 1015 (N-C, C-O); 1144 (S=O) cm^−1^.

MS (ESI), *m/z* (%): [M + Na]^+^ = 466.12 (100). Anal. Calcd for C_23_H_29_N_3_O_4_S, %: C 62.28; H 6.59; N 9.47. Found, %: C 62.36; H 6.56; N 9.52.

**4-Hydroxy-3-(2-mercapto-4-phenyl-1*H*-imidazol-1-yl)benzenesulfonamide (22)**

Resynthesis was carried out based on Ref. [32], and the spectral data is in consistent with an original one.

Yield 0.877 g, 50.5%, mp 161–163 °C.

**4-Ethoxy-*N,N*-diethyl-3-(2-(ethylthio)-4-phenyl-1*H*-imidazol-1-yl)benzenesulfonamide (23)**

The compound was obtained according to the procedure of compound **21**.

White solid, yield 1.1 g, 80%. 54–56 °C.

^1^H NMR (400 MHz, DMSO-*d*_6_) δ 7.93–7.86 (m, 2H, NCH, H_Ar_), 7.91 (s, 1H, H_Ar_), 7.89 (br. s, 1H, H_Ar_), 7.82 (d, *J* = 7.8 Hz, 2H, H_Ar_), 7.74 (s, 1H, H_Ar_), 7.45–7.36 (m, 3H, H_Ar_), 7.23 (t, *J* = 7.3 Hz, 1H, H_Ar_), 4.20 (q, *J* = 6.8 Hz, 2H, CH_2_), 3.19 (q, *J* = 7.0 Hz, 4H, CH_2_), 3.00 (q, *J* = 7.3 Hz, 2H, CH_2_), 1.26 (t, *J* = 6.9 Hz, 3H, CH_3_), 1.21 (t, *J* = 7.3 Hz, 3H, CH_3_), 1.05 (t, *J* = 7.0 Hz, 6H, CH_3_) ppm.

^13^C NMR (101 MHz, DMSO-*d*_6_) δ 156.41, 142.45, 140.98, 133.70, 131.44, 129.48, 128.56, 126.97, 126.66, 125.76, 124.26, 119.70, 113.92 (C_Ar_), 64.86, 41.80 (4CH_2_), 14.99, 14.23, 14.05 (4CH_3_) ppm.

IR (KBr) n 1333; 1298; 1274; 1219; 1199; 1089 (N-C, C-O); 1150 (S=O) cm^−1^.

Anal. Calcd for C_23_H_29_N_3_O_3_S_2_, %: C 60.10; H 6.36; N 9.14. Found, %: C 60.18; H 6.32; N 9.10.

**3-((2-Hydroxy-5-sulfamoylphenyl)amino)propanoic acid (24).**

A solution of amine **1** (9.41 g, 50 mmol) and acrylic acid (4.32 g, 60 mmol) in water (25 mL) was heated at reflux for 10 h, then cooled, and the formed precipitate was filtered off and purified by dissolving it in aqueous 2.5% sodium hydroxide, filtering, and acidifying the filtrate with 30% acetic acid to pH 6–7. The process was repeated twice.

White solid, yield 12.18 g, 94%, mp 182–184 °C.

^1^H NMR (400 MHz, DMSO-*d*_6_) δ 12.28 (s, 1H, OH), 10.15 (s, 1H, OH), 6.99 (s, 2H, NH_2_), 6.93 (d, *J* = 8.7 Hz, 2H, H_Ar_), 6.73 (d, *J* = 7.8 Hz, 1H, H_Ar_), 5.02 (s, 1H, NH), 3.30 (t, *J* = 6.7 Hz, 2H, NHC*H*_2_) 2.57 (t, *J* = 6.7 Hz, 2H, CH_2_CO) ppm.

^13^C NMR (101 MHz, DMSO-*d*_6_) δ 173.41 (C=O), 147.17, 137.16, 135.24, 114.25, 112.14, 106.41 (C_Ar_), 38.69, 33.38 (2CH_2_) ppm.

IR (KBr) n 3437; 3346 (O-H); 3258 (N-H); 1697 (C=O); 1290; 1107 (C-N); 1139 (S=O) cm^−1^.

Anal. Calcd for C_9_H_12_N_2_O_5_S, %: C 41.53; H 4.65; N 10.76. Found, %: C 41.61; H 4.68; N 10.72.

**Methyl 3-((2-hydroxy-5-sulfamoylphenyl)amino)propanoate (25).**

To a solution of acid **24** (13.01 g, 50 mmol) in methanol (100 mL), conc. sulfuric acid (1.83 g, 18.66 mmol) was added, and the mixture was refluxed for 5 h. After completion, methanol was evaporated under reduced pressure, and the residue was poured with aqueous 5% sodium carbonate until pH 7 was reached and boiled. Then, the aqueous solution was decanted, and the residue was washed with water (twice) by boiling the mixture. The obtained precipitate was recrystallized from propan-2-ol.

White solid, yield 9.65 g, 70%, mp 181–183 °C.

^1^H NMR (400 MHz, DMSO-*d*_6_) δ 10.86 (s, 1H, OH), 8.23 (br.s, 2H, H_Ar_, NH) 7.45–7.16 (m, 3H, H_Ar_, NH_2_), 6.92 (d, *J* = 8.2 Hz, 1H, H_Ar_), 3.61 (s, 3H, OCH_3_), 3.42 (t, *J* = 6.9 Hz, 2H, NHC*H*_2_), 2.71 (t, *J* = 6.8 Hz, 2H, CH_2_CO) ppm.

^13^C NMR (101 MHz, DMSO-*d*_6_) δ 171.47 (C=O), 150.11, 135.25, 130.74, 120.62, 114.32, 113.49 (C_Ar_), 52.79, 51.69, 31.87 (OCH_3_, 2CH_2_) ppm.

IR (KBr) n 3343 (O-H); 3232 (N-H); 1731 (C=O); 1286; 1097 (N-C); 1147 (S=O) cm^−1^.

Anal. Calcd for C_10_H_14_N_2_O_5_S, %: C 43.79; H 5.14; N 10.21. Found, %: C 43.70; H 5.10; N 10.25.

**3-((2-(1*H*-benzo[d]imidazol-2-yl)ethyl)amino)-4-hydroxybenzenesulfonamide (26)**

A solution of acid **24** (1.5 g, 5 mmol) and aromatic diamine (6 mmol) in 4M hydrochloric acid (25 mL) was refluxed for 24 h, then cooled, and aqueous 25% ammonia was added dropwise to reach pH 8. The formed solid was filtered off, washed with water, and purified by dissolving it in aqueous 2.5% sodium hydroxide, filtering, and acidifying the filtrate with 30% acetic acid to pH 6–7. The purification was repeated twice.

White solid, yield 0.19 g, 11.5%, mp 222–224 °C.

^1^H NMR (400 MHz, DMSO-*d*_6_) δ 12.30 (br. s, 1H, NH_Benz_), 10.19 (br. s, 1H, OH), 7.49 (br. s, 2H, H_Ar_), 7.13 (dd, *J* = 5.6, 3.1 Hz, 2H, H_Ar_), 7.04 (s, 1H, H_Ar_), 7.01 (s, 2H, NH_2_), 6.95 (d, *J* = 7.9 Hz, 1H, H_Ar_), 6.73 (d, *J* = 8.1 Hz, 1H, H_Ar_), 5.24 (s, 1H, NH), 3.63–3.51 (m, 2H, NHC*H*_2_), 3.16 (t, *J* = 7.0 Hz, 2H, CH_2_CO) ppm.

^13^C NMR (101 MHz, DMSO-*d*_6_) δ 153.18, 147.20, 137.24, 135.56, 135.26, 121.30, 117.72, 114.28, 112.16, 106.51 (C_Ar_), 41.03, 28.18 (2CH_2_) ppm.

IR (KBr) n 3392 (O-H); 3255 (N-H); 1283; 1073 (N-C); 1142 (S=O) cm^−1^.

Anal. Calcd for C_15_H_16_N_4_O_3_S, %: C 54.20; H 4.85; N 16.86. Found, %: C 54.29; H 4.89; N 16.82.

***N*** **-((3-Acetamido-4-hydroxyphenyl)sulfonyl)acetamide (27).**

To a solution of acid **24** (1.3 g, 5 mmol) in acetic anhydride (10 mL), conc. sulfuric acid (0.18 g, 1.8 mmol) was added, and the mixture was heated at reflux for 5 h. Then, the reaction mixture was cooled, the volatile fraction was evaporated under reduced pressure, and the residue was poured with aqueous 5% sodium carbonate solution (10 mL). The formed crystalline was filtered off, washed with water, and recrystallized from water.

White solid, yield 1.05 g, 76%, mp 258–260 °C.

^1^H NMR (400 MHz, DMSO-*d*_6_) δ 11.87 (s, 1H, NH), 11.06 (s, 1H, OH), 9.38 (s, 1H, NH), 8.51 (s, 1H, H_Ar_), 7.51 (d, *J* = 8.5 Hz, 1H, H_Ar_), 7.00 (d, *J* = 8.5 Hz, 1H, H_Ar_), 2.12 (s, 3H, CH_3_), 1.89 (s, 3H, CH_3_) ppm.

^13^C NMR (101 MHz, DMSO-*d*_6_) δ 169.19, 168.49 (2C=O), 151.89, 129.08, 126.65, 124.72, 120.79, 114.76 (C_Ar_), 23.81, 23.20 (2CH_3_) ppm.

IR (KBr) n 3240 (N-H, O-H); 1718; 1663 (C=O); 1291; 1081 (N-C); 1155 (S=O) cm^−1^.

Anal. Calcd for C_10_H_12_N_2_O_5_S, %: C 44.11; H 4.44; N 10.29. Found, %: C 44.24; H 4.45; N 10.32.

**1-(2-Hydroxy-5-sulfamoylphenyl)-5-oxopyrrolidine-3-carboxylic acid (28)**

A mixture of itaconic acid (7.81 g, 60 mmol) and 3-amino-4-hydroxybenzenesulfonamide (**1**) (9.41 g, 50 mol) was refluxed in water (40 mL) for 4 h and then cooled down. Then, the formed crystalline precipitate was filtered off, washed with water, and purified by dissolving it in aqueous 5% sodium hydroxide solution (60 mL), filtering, and acidifying the filtrate with hydrochloric acid to pH 5 to give the title compound **28**.

White solid, yield 13.36 g, 89%, mp 130–132 °C.

^1^H NMR (400 MHz, DMSO-*d*_6_) δ 12.68 (br.s, 1H, OH), 10.58 (s, 1H, OH), 7.63 (s, 1H, H_Ar_), 7.60 (d, *J* = 8.5 Hz, 1H, H_Ar_), 7.21 (s, 2H, NH_2_), 7.03 (d, *J* = 8.5 Hz, 1H, H_Ar_), 3.93–3.80 (m, 2H, CH_2_), 3.41–3.38 (m, 1H, CH), 2.76–2.56 (m, 2H, CH_2_CO) ppm.

^13^C NMR (101 MHz, DMSO-*d*_6_) δ 174.38, 172.44 (2C=O), 155.85, 134.79, 126.67, 126.40, 125.18, 116.73 (C_Ar_), 50.77, 36.24 (NCH_2_, CH_2_CO), 33.57 (CH) ppm.

IR (KBr) n 3555-2898 (N-H, O-H); 1720; 1702 (C=O); 1315; 1100 (C-N); 1166 (S=O) cm^−1^.

Anal. Calcd for C_11_H_12_N_2_O_6_S, %: C 44.00; H 4.03; N 9.33. Found, %: C 44.11; H 4.06; N 9.31.

**Methyl 1-(2-hydroxy-5-sulfamoylphenyl)-5-oxopyrrolidine-3-carboxylate (29)**

To a solution of carboxylic acid **28** (15.00 g, 50 mmol) in methanol (200 mL), a catalytic amount of concentrated sulfuric acid (3 mL) was added dropwise, and the mixture was heated at reflux for 8 h. The solvent was then evaporated under reduced pressure, and the residue was neutralized with 5% sodium carbonate solution to pH 8–9. After cooling, the obtained solid was filtered off, washed with plenty of water and hexane, and recrystallized from 2-propanol (60 mL) to give the title compound **29**.

White solid, yield 9.29 g, 59%, mp 123–125 °C.

^1^H NMR (400 MHz, DMSO-*d*_6_) δ 10.59 (s, 1H, OH), 7.63 (s, 1H, H_Ar_), 7.60 (d, *J* = 8.5 Hz, 1H, H_Ar_), 7.21 (s, 2H, NH_2_), 7.03 (d, *J* = 8.5 Hz, 1H, H_Ar_), 3.95–3.84 (m, 2H, NCH_2_), 3.68 (s, 3H, CH_3_), 3.56–3.47 (m, 1H, CH), 2.76–2.61 (m, 2H, CH_2_CO) ppm.

^13^C NMR (101 MHz, DMSO-*d*_6_) δ 173.27, 172.12 (2C=O), 155.84, 134.76, 126.66, 126.42, 125.04, 116.71 (C_Ar_), 52.19 (OCH_3_), 50.50, 36.02 (NCH_2_, CH_2_CO), 33.43 (CH) ppm.

IR (KBr) n 3369 (O-H); 3268 (N-H) 1728; 1679 (C=O); 1221 (C-O); 1332; 1078 (C-N); 1162 (S=O) cm^−1^.

Anal. Calcd for C_12_H_14_N_2_O_6_S, %: C 45.86; H 4.49; N 8.91. Found, %: C 45.79; H 4.53; N 8.84.

**1-(2-Hydroxy-5-sulfamoylphenyl)-5-oxo-*N*-phenylpyrrolidine-3-carboxamide (30)**

To a solution of acid **28** (1.5 g, 5 mmol), aniline (0.56 g, 6 mmol), and HBTU (7.58 g, 20 mmol) in DMF (10 mL), triethylamine (2.02 g, 20 mmol) was added dropwise, and the mixture was stirred at room temperature for 24 h. After completion of the reaction, the mixture was diluted with water (40 mL) and the formed solid was filtered off, washed with water, and recrystallized from propan-2-ol.

White solid, yield 1.15 g, 61%, mp 195–198 °C.

^1^H NMR (400 MHz, DMSO-*d*_6_) δ 10.59 (s, 1H, NH), 10.16 (s, 1H, OH), 7.68–7.59 (m, 4H, H_Ar_), 7.32 (t, *J* = 7.8 Hz, 2H, H_Ar_), 7.22 (s, 2H, NH_2_), 7.10–7.03 (m, 2H, H_Ar_), 4.00–3.80 (m, 2H, NCH_2_), 3.55–3.48 (m, 1H, CH), 2.72 (d, *J* = 8.4 Hz, 2H, CH_2_CO) ppm.

^13^C NMR (101 MHz, DMSO-*d*_6_) δ 172.55, 171.05 (2C=O), 155.82, 138.98, 134.80, 128.81, 126.67, 126.34, 125.20, 123.53, 119.39, 116.68 (C_Ar_), 51.42, 38.04 (NCH_2_, CH_2_CO), 34.15 (CH) ppm.

IR (KBr) n 3362 (O–H); 3254 (N–H); 1595; 1578 (C=O); 1281; 1093 (N–C); 1142 (S=O) cm^−1^.

Anal. Calcd for C_17_H_17_N_3_O_5_S, %: C 54.39; H 4.56; N 11.19. Found, %: C 54.46; H 4.53; N 11.14.

**4-(*1H*-benzo[d]imidazol-2-yl)-2-oxopyrrolidin-1-yl)-4-hydroxybenzenesulfonamide (31)**

A solution of compound **28** (2 g, 5.6 mmol) and benzene-1,2-diamine (1.21 g, 11.2 mmol) in 4M hydrochloric acid (35 mL) was heated at reflux for 24 h, then cooled, and the formed hydrochloride salt was filtered off and washed with acetone. The product was purified by dissolving it in hot water and adding 25% ammonia to pH 8.

White solid, yield 1.24 g, 66%, mp 280–283 °C.

^1^H NMR (400 MHz, DMSO-*d*_6_) δ 12.63 (s, 1H, NH), 11.66 (s, 1H, OH), 7.71 (s, 1H, H_Ar_), 7.66 (d, *J* = 8.6 Hz, 1H, H_Ar_), 7.55 (br. s, 2H, H_Ar_), 7.27–7.19 (m, 4H, NH_2_, H_Ar_), 7.10 (d, *J* = 8.6 Hz, 1H, H_Ar_), 4.18–4.00 (m, 3H, CH, NCH_2_), 3.09–3.01 (dd, *J* = 16.4, 8.3 Hz, 1H, CH_2_CO), 2.68 (dd, *J* = 16.4, 4.5 Hz, 1H, CH_2_CO) ppm.

^13^C NMR (101 MHz, DMSO-*d*_6_) δ 172.49 (C=O), 156.65, 156.00, 134.66, 126.96, 126.78, 124.83, 122.02, 117.14 (C_Ar_), 53.05, 36.61 (NCH_2_, CH_2_CO), 32.19 (CH) ppm.

IR (KBr) n 3600-3277 (O-H, N-H); 1672 (C=O); 1625 (C=N); 1300; 1085 (N-C); 1149 (S=O) cm^−1^.

Anal. Calcd for C_17_H_16_N_4_O_4_S, %: C 54.83; H 4.33; N 15.05. Found, %: C 54.91; H 4.33; N 15.10.

**4-Hydroxy-3-(4-(5-methyl-*1H*-benzo[d]imidazol-2-yl)-2-oxopyrrolidin-1-yl)benzenesulfonamide (32)**

White solid, yield 0.75 g, 39%, mp 175–177 °C.

^1^H NMR (400 MHz, DMSO-*d*_6_) δ 12.48 (s, 1H, NH), 11.86 (s, 1H, OH), 7.69 (s, 1H, H_Ar_), 7.65 (m, *J* = 8.8 Hz, 1H, H_Ar_), 7.42 (d, *J* = 7.7 Hz, 1H, H_Ar_), 7.33 (s, 1H, H_Ar_), 7.23 (s, 2H, NH_2_), 7.08 (d, *J* = 8.5 Hz, 1H, H_Ar_), 7.02 (d, *J* = 8.1 Hz, 1H, H_Ar_), 3.94–4.16 (m, 3H, CH, NCH_2_), 3.03 (dd, *J* = 16.4, 8.4 Hz, 1H, CH_2_CO), 2.66 (dd, *J* = 16.4, 3.8 Hz, 1H, CH_2_CO), 2.41 (s, 3H, CH_3_) ppm.

^13^C NMR (101 MHz, DMSO-*d*_6_) δ 172.48 (C=O), 156.78, 134.60, 131.30, 126.81, 124.77, 123.39, 117.19 (C_Ar_), 53.05, 36.72 (NCH_2_, CH_2_CO), 32.22 (CH), 21.25 (CH_3_) ppm.

IR (KBr) n 3569 (O-H); 3195 (N-H); 1682 (C=O); 1573 (C=N); 1284; 1068 (N-C); 1147 (S=O) cm^−1^.

Anal. Calcd for C_18_H_18_N_4_O_4_S, %: C 55.95; H 4.70; N 14.50. Found, %: C 55.91; H 4.75; N 14.54.

**3-(4-(6-Fluoro-*1H*-benzo[d]imidazol-2-yl)-2-oxopyrrolidin-1-yl)-4-hydroxybenzenesulfonamide (33)**

White solid, yield 1.11 g, 57%, mp 289–291 °C.

IR (KBr) n 3600–3208 (O–H, N–H); 1667 (C=O); 1573 (C=N); 1273; 1065 (N–C); 1157 (S=O) cm^−1^.

^1^H NMR (400 MHz, DMSO-*d*_6_) δ 12.72 (s, 1H, NH), 11.22 (s, 1H, OH), 7.70 (s, 1H, H_Ar_), 7.64 (d, *J* = 8.6 Hz, 1H, H_Ar_), 7.57–7.51 (m, 1H, H_Ar_), 7.36 (d, *J* = 9.3 Hz, 1H, H_Ar_), 7.23 (s, 2H, NH_2_), 7.09–7.02 (m, 2H, H_Ar_), 4.16–4.00 (m, 3H, CH, NCH_2_), 3.00 (dd, *J* = 16.5, 8.0 Hz, 1H, CH_2_CO), 2.77 (dd, *J* = 16.5, 5.2 Hz, 1H, CH_2_CO) ppm.

^13^C NMR (101 MHz, DMSO-*d*_6_) δ 172.44 (CO), 158.50 (d, *J* = 234.9 Hz, CF), 157.33, 156.33, 134.73, 126.74 (d, *J* = 20.3 Hz, CF), 124.93, 116.97 (C_Ar_), 109.88 (d, *J* = 23.2 Hz, CF), 52.94, 36.28 (NCH_2_, CH_2_CO), 32.08 (CH) ppm.

Anal. Calcd for C_17_H_15_FN_4_O_4_S, %: C 52.30; H 3.87; N 14.35. Found, %: C 52.23; H 3.87; N 14.29.

**3-(4-(6-Chloro-*1H*-benzo[d]imidazol-2-yl)-2-oxopyrrolidin-1-yl)-4-hydroxybenzenesulfonamide (34)**

The compound was prepared from 4-chloro-1,2-diaminobenzene (0.85 g, 6 mmol) by the method applied for the synthesis of compound **31**.

White solid, yield 1.05 g, 52%, mp 312–315 °C.

^1^H NMR (400 MHz, DMSO-*d*_6_) δ 12.77 (br.s, 1H, NH), 11.14 (s, 1H, OH), 7.64 (d, *J* = 8.6 Hz, 1H, H_Ar_), 7.61 (s, 1H, H_Ar_), 7.55 (d, *J* = 8.4 Hz, 1H, H_Ar_), 7.26–7.18 (m, 3H, NH_2_, H_Ar_), 7.07 (d, *J* = 8.6 Hz, 1H, H_Ar_), 4.17–3.99 (m, 3H, CH, NCH_2_), 3.05–2.95 (m, 1H, CH_2_CO), 2.85–2.75 (m, 1H, CH_2_CO) ppm.

^13^C NMR (101 MHz, DMSO-*d*_6_) δ 172.45 (C=O), 157.27, 156.28, 134.75, 127.27, 127.07, 126.84, 126.64, 126.26, 122.11, 116.96 (C_Ar_), 52.92, 36.20 (NCH_2_, CH_2_CO), 32.05 (CH) ppm.

IR (KBr) n 3600–3207 (O–H, N–H) 1664 (C=O); 1571 (C=N); 1266; 1067 (N–C); 1157 (S=O); 825 (C–Cl) cm^−1^.

Anal. Calcd for C_17_H_15_ClN_4_O_4_S, %: C 50.19; H 3.72; N 13.77. Found, %: C 50.10; H 3.76; N 13.80.

**Ethyl 1-(2-ethoxy-5-sulfamoylphenyl)-5-oxopyrrolidine-3-carboxylate (35)**

To a solution of compound **28** (15 g, 50 mmol), dimethylformamide potassium hydroxide (4.61 g, 70 mmol) and potassium carbonate (5 g, 36 mmol) were slowly added, and the mixture was left to stir at room temperature for 15 min. Afterwards, ethyl iodide (15.6 g, 100 mmol) was added dropwise, and the mixture was stirred at room temperature for 14 h. When the reaction was completed (TLC), the excess ethyl iodide was evaporated under reduced pressure, and the residue was diluted with water (150 mL). The formed precipitate was filtered off, washed with water, and recrystallized from propan-2-ol.

White solid, yield 10.31 g, 58%, mp 110–112 °C

^1^H NMR (400 MHz, DMSO-*d*_6_) δ 7.74 (d, *J* = 8.8 Hz, 1H, H_Ar_), 7.70 (s, 2H, H_Ar_), 7.30 (s, 2H, NH_2_), 7.27 (d, *J* = 8.8 Hz, 1H, H_Ar_), 4.31–4.00 (m, 4H, CH_2_), 3.99–3.79 (m, 2H, NCH_2_), 3.53–3.38 (m, 1H, CH), 2.83–2.56 (m, 2H, CH_2_CO), 1.33 (t, *J* = 6.8 Hz, 3H, CH_3_), 1.22 (t, *J* = 7.0 Hz, 3H, CH_3_) ppm.

^13^C NMR (101 MHz, DMSO-*d*_6_) δ 172.80, 172.15 (C=O), 156.12, 135.96, 126.57, 126.451, 126.38, 113.26 (C_Ar_), 64.41, 60.80, 50.70, 36.14, 33.33 (4CH_2_, CH), 14.40, 14.04 (2CH_3_) ppm.

IR (KBr) n 3298 (N–H); 1729; 1668 (C=O); 1336; 1148 (C–O); 1273; 1094 (N–C); 1162 (S=O) cm^−1^.

Anal. Calcd for C_15_H_20_N_2_O_6_S, %: C 50.55; H 5.66; N 7.86. Found, %: C 50.50; H 5.61; N 7.91.

**1-(2-Ethoxy-5-sulfamoylphenyl)-5-oxopyrrolidine-3-carboxylic acid (36)**

A solution of compound **35** (1.07 g, 3 mmol) in aqueous 10% hydrochloric acid (10 mL). was refluxed for 2 h, then cooled, and the obtained precipitate was filtered off, washed with diethyl ether, and recrystallized from diethyl ether.

White solid, yield 0.79 g, 80.5%, mp 173–175 °C.

^1^H NMR (400 MHz, DMSO-*d*_6_) δ 12.73 (s, 1H, OH), 7.73 (d, *J* = 8.8 Hz, 1H, H_Ar_), 7.69 (s, 2H, H_Ar_), 7.29 (s, 2H, NH_2_), 7.26 (d, *J* = 8.8 Hz, 1H, H_Ar_), 4.14 (q, *J* = 6.7 Hz, 2H, CH_2_), 4.01–3.72 (m, 2H, NCH_2_), 3.40–3.34 (m, 1H, CH), 2.77–2.54 (m, 2H, CH_2_CO), 1.34 (t, *J* = 6.8 Hz, 3H, CH_3_) ppm.

^13^C NMR (101 MHz, DMSO-*d*_6_) δ 174.27, 172.38 (2C=O), 156.14, 135.93, 126.67, 126.41, 126.38, 113.24 (C_Ar_), 64.42, 50.90, 36.33 (3CH_2_), 33.44 (CH), 14.42 (CH_3_) ppm.

IR (KBr) n 3254 (N–H); 3088 (O–H); 1728; 1683 (C=O); 1306 (C–O); 1286; 1089 (N–C); 1161 (S=O) cm^−1^.

Anal. Calcd for C_13_H_16_N_2_O_6_S, %: C 47.56; H 4.91; N 8.53. Found, %: C 47.62; H 4.86; N 8.49.

**3-(4-(1*H*-benzo[d]imidazol-2-yl)-2-oxopyrrolidin-1-yl)-4-ethoxybenzenesulfonamide (37)**

A solution of compound **35** (2 g, 5.6 mmol) and *o*-phenylenediamine (1.21 g, 11.2 mmol) in aqueous 4M hydrochloric acid (35 mL) was heated at reflux for 24 h. After completion of the reaction, the mixture was cooled, and the formed hydrochloride salt was filtered off and washed with acetone. To obtain pure target solid, the salt was dissolved in hot water and the solution was treated with aqueous 25% ammonia to pH 8.

White solid, yield 0.75 g, 33.5%, mp 138–140 °C.

^1^H NMR (400 MHz, DMSO-*d*_6_) δ 12.49 (s, 1H, NH), 7.76 (s, 1H, H_Ar_), 7.74 (d, *J* = 8.9 Hz, 1H, H_Ar_), 7.54 (dd, *J* = 5.1, 3.4 Hz, 2H, H_Ar_), 7.31 (s, 2H, NH_2_), 7.26 (d, *J* = 8.7 Hz, 1H, H_Ar_), 7.17 (dd, *J* = 5.5, 3.1 Hz, 2H, H_Ar_), 4.19–3.97 (m, 5H, CH, NCH_2_, CH_2_), 3.04–2.91 (m, 2H, CH_2_CO), 1.23 (t, *J* = 6.9 Hz, 3H, CH_3_) ppm.

^13^C NMR (101 MHz, DMSO-*d*_6_) δ 172.60 (C=O), 156.12, 155.01, 138.79, 135.95, 126.79, 126.41, 126.37, 121.74, 115.29, 115.02, 113.27 (C_Ar_), 64.38, 53.20, 35.49 (3CH_2_), 31.92 (CH), 14.31 (CH_3_) ppm.

IR (KBr) n 3491 (N–H); 1659 (C=O); 1596 (C=N); 1341; 1270; 1067 (N–C, C–O); 1158 (S=O) cm^−1^.

Anal. Calcd for C_19_H_20_N_4_O_4_S, %: C 56.99; H 5.03; N 13.99. Found, %: C 57.06; H 5.02; N 13.94.

**3-(4-(5-Chloro-1*H*-benzo[d]imidazol-2-yl)-2-oxopyrrolidin-1-yl)-4-ethoxybenzenesulfonamide (38)**

A solution of compound **35** (1.78 g, 5 mmol) and 4-chloro-1,2-diaminobenzene (0.85 g, 6 mmol) in aqueous 4M hydrochloric acid (35 mL) was heated at reflux for 24 h. After completion of the reaction, the mixture was cooled and treated with aqueous 25% ammonia to pH 8. The formed solid was filtered off, washed with water, and recrystallized from acetone.

White solid, yield 1.25 g, 57.5%, mp 160–162 °C.

^1^H NMR (400 MHz, DMSO-*d*_6_) δ 7.87 (s, 1H, H_Ar_), 7.81 (s, 2H, H_Ar_), 7.80–7.70 (m, 1H, H_Ar_), 7.52 (d, *J* = 8.7 Hz, 1H, H_Ar_), 7.52 (d, *J* = 8.7 Hz, 1H, H_Ar_), 7.34 (br. s, 2H, NH_2_), 7.28 (d, *J* = 8.7 Hz, 1H, H_Ar_), 4.44–4.34 (m 1H, CH), 4.23 (t, *J* = 8.9 Hz, 1H, NCH_2_), 4.19–4.04 (m, 3H, NCH_2_, CH_2_), 3.21–3.00 (m, 2H, CH_2_), 1.25 (t, *J* = 6.8 Hz, 3H, CH_3_) ppm.

^13^C NMR (101 MHz, DMSO-*d*_6_) δ 171.46 (C=O), 156.09, 155.33_,_ 135.98, 131.31, 133.33,129.35, 126.70, 126.52, 126.23, 125.36, 115.59, 113.87, 113.33 (C_Ar_), 64.46, 52.13, 34.79, 30.89 (3CH_2_, CH), 14.33 (CH_3_) ppm.

IR (KBr) n 3319 (N-H); 1678 (C=O); 1596 (C=N); 1335; 1267; 1040 (N-C, C-O); 1162 (S=O); 807 (C-Cl) cm^−1^.

Anal. Calcd for C_19_H_19_ClN_4_O_4_S, %: C 52.47; H 4.40; N 12.88. Found, %: 52.39; H 4.44; N 12.92.

### 3.2. Affinity Measurements

Twelve catalytically active recombinant human carbonic anhydrases were produced and purified as previously reported [48,49]. For transmembrane isoenzymes, only catalytic domains were produced. The compound affinities were measured using the fluorescent thermal shift assay (FTSA). The dissociation constant (*K*_d_) for each protein–ligand pair was determined from a series of nine experimental points. Each 10 μL sample contained a solution of 5–10 μM of tested CA and a solution of the ligand at various concentrations (prepared by serial dilution) ranging from 0 to 200 μM in 50 mM phosphate buffer containing 100 mM NaCl and 4% DMSO at pH 7.0. The prepared samples were analyzed using a Rotor-Gene Q qPCR instrument (Qiagen, Hilden, Germany). A blue filter (excitation at 365 ± 20 nm, detection at 460 ± 15 nm) was used for the measurements. The initial temperature was 25 °C, and the final temperature was 99 °C; the temperature steadily increased from the initial temperature at a rate of 1 °C/min, and the fluorescence of the samples was measured every 30 s.

The raw experimental FTSA data were analyzed using the Thermott software online tool (https://thermott.com, accessed on 18 April 2023) [36]. The determined melting temperature (*T*_m_) of each sample was then used to acquire the ligand dosing curve and the protein–ligand dissociation constant obtained by fitting the thermal stabilization model to the experimental data.

### 3.3. Crystallization and Structure Solution

The complex of CAI with compound **25** was prepared by mixing the protein with a four-fold excess of **25** (50 mM solution in DMSO). The complex was concentrated to 35 mg/mL and crystallized by the sitting drop method. Crystals of the CAII-**25** complex were prepared by soaking crystals with reservoir solution supplemented with inhibitor at a concentration of 1 mM. Reservoir solutions and cryo-protection buffers are described in Supplementary Table S1.

All datasets were collected at beamline P13 operated by EMBL Hamburg at the PETRA III storage ring (DESY, Hamburg, Germany). Collected datasets were integrated by XDS [50] and scaled using AIMLESS v.0.7.4 and other CCP4 tools v.7.1.002 [51]. The structure was solved by molecular replacement using MOLREP v.11.7.02 [52] and 2CAB as an initial model. The model was refined by REFMAC v.5.8.0258 [53] and rebuilt in COOT v.0.9 [54]. The inhibitor model was created and minimized using AVOGADRO v.1.2.0 [55].

### 3.4. Biological Experiments in Cancer Cells In Vitro

Human triple-negative breast cancer MDA-MB-231, human glioblastoma (U-87), and human prostate adenocarcinoma (PPC-1) were obtained from the American Type Culture Collection (ATCC, Manassas, VA, USA). Human foreskin fibroblasts (HFs), CRL-4001, also originally sourced from ATCC, were kindly provided by Prof. Helder Santos (University of Helsinki, Helsinki, Finland). All cell lines were cultivated in Dulbecco’s modified Eagle’s GlutaMAX medium (Gibco, Carlsbad, CA, USA) enriched with 10,000 U/mL penicillin, 10 mg/mL streptomycin (Gibco, Waltham, MA, USA), and 10% fetal bovine serum (Gibco). Cell cultures were maintained at 37 °C in a humidified atmosphere containing 5% CO_2_ and used up to passage 20.

To evaluate the effects of the compounds on cell viability, the MTT assay was employed, as previously described [56]. Briefly, cells were seeded in triplicate into 96-well tissue culture-treated flat-bottom plates at a density of 4 × 10^3^ cells/well for U-87, MDA-MB-231, and PPC-1, and 5 × 10^3^ cells/well for HFs. After a 24 h incubation, cells were treated with 100 μM of the test compounds. After 72 h, the 3-(4,5-dimethylthiazol-2-yl)-2,5-diphenyltetrazolium bromide (MTT; Sigma-Aldrich Co., St Louis, MO, USA) was added, and the resulting formazan crystals were dissolved in 2-propanol (Sigma-Aldrich Co., St. Louis, MO, USA). Absorbance was measured at 570 and 630 nm using multidetection microplate reader Multiskan GO (Thermo Fisher Scientific Oy, Ratastie, Finland).

The EC_50_ values of the most active compounds—**9**, **12**, **18**, and **21**—and the comparative compounds acetazolamide (AZM) and U-104 (Sigma-Aldrich Co., St. Louis, MO, USA) were determined using the same MTT method. Compounds **9**, **12**, **18**, and **21** were serially diluted from 50 µM to 1.56 µM, and AZM and U-104–from 500 µM to 15.6 µM, before being added to the cells. The EC_50_, defined as the concentration reducing cell metabolic activity by 50%, was calculated using the Hill equation.

The compound activity in 3D cell cultures (cancer spheroids) was assessed by examining their effects on spheroid growth and cell viability. Briefly, cells at 70% confluence were incubated with Nanoshuttle (n3D Biosciences, Inc., Houston, TX, USA) for 8 h. The cells were then trypsinized, centrifuged, and seeded into ultra-low-attachment 96-well plates at a density of 1.5 × 10^3^ cancer cells and 1.5 × 10^3^ fibroblasts per well. Plates were then incubated for two days at 37 °C in a humidified environment on a magnetic drive. Subsequently, medium containing 10 µM of the test compounds **9** and **21** was added. Spheroid images were taken every two days using an Olympus IX73 inverted microscope (Olympus Corporation, Tokyo, Japan), and spheroid sizes were analyzed using ImageJ version 1.53o (National Institutes of Health, Bethesda, MD, USA) and Microsoft Excel for Mac 2025 software version 16.95.4 (Microsoft Corporation, Redmond, WA, USA). On the final day of the experiment, 20 µL of MTT reagent was added to each well. After a 10 h incubation, the medium was removed, and the formazan crystals were dissolved in 100 µL of DMSO overnight. Absorbance was measured at 570 and 630 nm using the Multiskan GO microplate reader (Thermo Fisher Scientific Oy, Ratastie, Finland).

All biological experiments were conducted at least three times, and results were expressed as mean ± standard deviation. Data were analyzed using Microsoft Excel for Mac 2025 software. The statistical analysis was performed using Student’s *t*-test. The level of significance was set as *p* < 0.05.

### 3.5. Statistical Analysis

All biological experiments were repeated at least three times, calculating the mean and standard deviation. The data were processed using Microsoft Excel for Mac 2025 software version 16.95.4 (Microsoft Corporation, Redmond, WA, USA). Statistical analysis was performed by using Student’s *t*-test. The level of significance was set as *p* < 0.05.

## 4. Conclusions

In this study, we successfully synthesized and characterized a series of novel functionalized Schiff base, aminoketone, 5-oxopyrrolidine, imidazole, and *β*-amino acid derivatives on the basis of 3-amino-4-hydroxybenzenesulfonamide and evaluated their biological activity. We assessed the binding affinities of synthesized compounds to recombinant human carbonic anhydrases using the fluorescent thermal shift assay. The compounds were grouped into five categories based on their structural similarities.

Our structure–activity analysis indicated that adding groups such as ethyl or acetyl to the primary sulfonamide fragment diminished the binding ability of certain compounds (specifically **21**, **23**, and **27**) to the CA isoenzymes. Overall, the binding affinities of the primary sulphonamide compounds for the cancer-associated CAIX isoenzyme were in the range of 0.51–22 µM and for CAXII in the range of 6.2–75 μM.

Compounds that featured an -OEt group instead of an -OH group (compounds **35**, **36**, **37**, and **38**) exhibited stronger binding than their hydroxy group counterparts. Additionally, aminoketone compounds (**12**, **14**, and **18**) showed good binding to CAI, CAII, CAVII, CAIX, and CAXIV isoenzymes, with dissociation constants (*K*_d_ values) in the range of 0.0062–0.98 µM.

Moreover, compounds from the imidazole group (**19**) and the *β*-amino acid group (**25**) also demonstrated high affinity for CAI and CAII. Structural analysis revealed that the benzene ring of compound **25** was oriented differently in the active sites of CAI and CAII. In both instances, the substituent at position 3 was flexible and could adopt alternative conformations: one oriented toward the hydrophilic side and the other toward the hydrophobic side of the cavity.

Generally, most compounds were not very active against human glioblastoma U-87, triple-negative breast cancer MDA-MB-231, or prostate adenocarcinoma PPC-1 cell lines. Only four compounds (**9**, **12**, **18**, and **21**) reduced the viability of at least one of the tested cell lines up to <20%. These compounds showed very distinct CA inhibitory profiles: compounds **12** and **18** were characterized by relatively low K_d_s towards most CAs, while compound **9** showed a modest affinity and compound **21** did not bind to CAs.

The EC_50_ values of the most active compounds varied in a wide range from 13.0 to 148.3 μM against different cell lines. However, even though their cytotoxicity was not very high, all four compounds were more active against the triple-negative breast cancer cell line MDA-MB-231 than CA-IX inhibitor U-104, and more active against all cell lines than the non-selective CA inhibitor acetazolamide.

Compound **9** (inhibitor of multi-CAs) and compound **21** (not binding to CAs) were identified as the most promising candidates. EC_50_ values for compound **9** ranged from 13.0 to 23.3 μM and for compound **21**–from 43.7 to 148.3 μM. Both compounds reduced the viability of spheroids formed from all cancer cell lines. U-87 and PPC-1 spheroids became looser in the presence of compound **9**, while the growth of MDA-MB-231 spheroids was slower compared to the control. Compound **21** reduced the growth of U-87 and MDA-MB-231 3D cultures, with no significant effect on PPC-1 spheroids.

The study results suggest that the strongest binding to CAs is not the only or the most important factor to achieve a higher effect in cancer cell 2D and 3D cultures. Activity in the cell monolayer and spheroids could also be determined by different testing conditions, including cell line, CA expression, oxygen concentration, cell permeability, off-target effects, etc. The biological activity of 3-amino-4-hydroxy-benzenesulfonamides could be related to different mechanisms of action. However, we recognize the limitations of our study, which did not include the determination of CA expression or hypoxia level, and suggest that further thorough analysis is needed to come to clearer conclusions on the correlations between distinct binding to CAs and biological activity in clearly defined in vitro tumor models.

## Figures and Tables

**Table 1:** Affinity of recombinant human CA isoenzymes with studied compounds as determined by FTSA, with dissociation constants (*K*_d_, μM).

Compound	CAI	CAII	CAIII	CAIV	CAVA	CAVB	CAVI	CAVII	CAIX	CAXII	CAXIII	CAXIV
**1**	130	17	≥200	20	130	nd	110	100	15	40	140	22
**2**	≥200	20	≥200	28	≥200	nd	89	120	16	33	130	26
**3**	90	18	≥200	29	140	nd	79	100	16	58	160	22
**4**	120	11	≥200	14	140	nd	39	83	13	28	150	22
**5**	150	23	≥200	12	≥200	nd	120	100	17	43	130	16
**6**	47	13	≥200	37	67	nd	93	112	8.4	54	98	20
**7**	59	16	≥200	5.5	170	nd	130	120	18	41	≥200	30
**8**	120	16	≥200	9.9	nd	nd	79	92	16	41	120	21
**9**	76	17	≥200	9.1	13	nd	80	31	9	65	100	0.68
**10**	3.1	0.14	≥200	13	≥200	nd	4.5	0.49	2.3	17	6.9	0.36
**11**	6	0.15	≥200	3.7	29	nd	12	0.87	2.1	19	12	0.67
**12**	0.0062	0.097	≥200	17	8	0.88	20	0.24	0.98	27	5.5	0.34
**13**	7.7	3.6	≥200	7.3	85	nd	82	22	4.3	37	26	5.2
**14**	0.052	0.16	≥200	11	22	nd	18	0.39	0.51	20	4	0.78
**15**	60	8	≥200	1.9	≥200	nd	2.4	17	1.9	11	27	1.6
**16**	30	7.3	≥200	3	≥200	nd	5.1	7.5	1.5	15	28	1.7
**17**	180	14	≥200	23	160	93	4.7	27	4.8	56	25	2.9
**18**	0.0077	0.11	≥200	6.6	14	nd	13	0.25	0.53	6.2	3.2	0.27
**19**	0.52	3	≥200	34	160	nd	17	32	5.3	25	15	3.3
**20**	21	10	≥200	17	180	nd	12	140	16	18	28	1.6
**21**	≥200	≥200	≥200	≥200	nd	nd	≥200	≥200	≥200	≥200	≥200	≥200
**22**	28	9.8	≥200	7.1	≥200	nd	32	100	0.98	41	14	5.9
**23**	≥200	≥200	≥200	≥200	≥200	nd	≥200	≥200	≥200	≥200	≥200	≥200
**24**	≥200	≥200	≥200	≥200	≥200	≥200	≥200	≥200	22	33	≥200	≥200
**25**	1.6	5.4	≥200	12	≥200	nd	120	150	4.2	74	39	9
**26**	≥200	24	≥200	7.6	35	nd	≥200	180	14	74	65	21
**27**	≥200	≥200	≥200	≥200	≥200	nd	≥200	≥200	≥200	≥200	≥200	≥200
**28**	≥200	47	≥200	≥200	≥200	≥200	≥200	100	14	57	90	46
**29**	≥200	58	≥200	54	≥200	96	140	≥200	7.1	75	47	34
**30**	≥200	22	≥200	100	29	3	48	43	14	48	16	26
**31**	≥200	16	≥200	24	120	1.1	98	25	9.7	59	19	14
**32**	≥200	11	≥200	9.2	150	4	150	18	8.2	48	21	12
**33**	160	16	≥200	24	87	3.7	120	28	8.2	72	22	16
**34**	110	10	≥200	16	66	4.9	96	15	7.1	50	17	11
**35**	≥200	8.1	≥200	4	≥200	nd	38	62	0.39	12	18	0.48
**36**	≥200	43	≥200	52	≥200	nd	96	92	2.3	6.1	42	5
**37**	≥200	15	≥200	2.2	130	nd	49	12	1.2	8.9	25	0.94
**38**	≥200	8.6	≥200	2.1	58	nd	2.9	49	0.83	9.1	15	0.92
AZM *	2.4	0.046	40	0.087	0.84	0.14	0.22	0.013	0.021	0.13	0.12	0.063
U-104 *	1.1	0.33	≥200	3.1	6	0.43	10	0.25	0.1	5.5	2.2	0.27

nd—Not determined. * Published data [37].

**Table 2:** Established EC_50_ values for the most active and comparative compounds.

Compound	EC_50_ (μM)			
	U-87	MDA-MB-231	PPC-1	HF
**9**	23.3 ± 2.9	14.0 ± 3.2	13.0 ± 3.5	13.8 ± 5.2
**12**	137.0 ± 15.4	147.7 ± 22.5	137.3 ± 15.5	166.0 ± 7.0
**18**	105.0 ± 6.0	126.7 ± 20.8	107.5 ± 17.7	134.3 ± 11.1
**21**	148.3 ± 10.4	57.3 ± 15.0	43.7 ± 15.0	259.3 ± 8.4
U-104	ND	239.8 ± 18.0	ND	ND
AZM	>500	>500	>500	>500

Abbreviations: ND—not determined, AZM—acetazolamide.

## Data Availability

Data are contained within the article and Supplementary Materials. The compounds are available from the corresponding author on a reasonable request.

## Supplementary Materials

The following supporting information can be downloaded at: https://www.mdpi.com/article/10.3390/ijms26136466/s1.

## References

[1] Rutkauskas K., Zubrienė A., Tumosienė I., Kantminienė K., Mickevičius V., Matulis D. (2017). Benzenesulfonamides Bearing Pyrrolidinone Moiety as Inhibitors of Carbonic Anhydrase IX: Synthesis and Binding Studies. Med. Chem. Res..

[2] Balandis B., Ivanauskaitė G., Smirnovienė J., Kantminienė K., Matulis D., Mickevičius V., Zubrienė A. (2020). Synthesis and Structure–Affinity Relationship of Chlorinated Pyrrolidinone-Bearing Benzenesulfonamides as Human Carbonic Anhydrase Inhibitors. Bioorg. Chem..

[3] Vaškevičienė I., Paketurytė V., Pajanok N., Žukauskas Š., Sapijanskaitė B., Kantminienė K., Mickevičius V., Zubrienė A., Matulis D. (2019). Pyrrolidinone-Bearing Methylated and Halogenated Benzenesulfonamides as Inhibitors of Carbonic Anhydrases. Bioorg. Med. Chem..

[4] Afifi N., Barrero C.A. (2023). Understanding Breast Cancer Aggressiveness and Its Implications in Diagnosis and Treatment. JCM.

[5] Roda D., Veiga P., Melo J.B., Carreira I.M., Ribeiro I.P. (2024). Principles in the Management of Glioblastoma. Genes.

[6] Bray F., Ferlay J., Soerjomataram I., Siegel R.L., Torre L.A., Jemal A. (2018). Global Cancer Statistics 2018: GLOBOCAN Estimates of Incidence and Mortality Worldwide for 36 Cancers in 185 Countries. CA A Cancer J. Clin..

[7] Ward C., Meehan J., Mullen P., Supuran C., Dixon J.M., Thomas J.S., Winum J.-Y., Lambin P., Dubois L., Pavathaneni N.-K. (2015). Evaluation of Carbonic Anhydrase IX as a Therapeutic Target for Inhibition of Breast Cancer Invasion and Metastasis Using a Series of in Vitro Breast Cancer Models. Oncotarget.

[8] Haapasalo J., Nordfors K., Haapasalo H., Parkkila S. (2020). The Expression of Carbonic Anhydrases II, IX and XII in Brain Tumors. Cancers.

[9] Zhao K., Schäfer A., Zhang Z., Elsässer K., Culmsee C., Zhong L., Pagenstecher A., Nimsky C., Bartsch J.W. (2021). Inhibition of Carbonic Anhydrase 2 Overcomes Temozolomide Resistance in Glioblastoma Cells. Int. J. Mol. Sci..

[10] Smyth L.G., O’Hurley G., O’Grady A., Fitzpatrick J.M., Kay E., Watson R.W.G. (2010). Carbonic Anhydrase IX Expression in Prostate Cancer. Prostate Cancer Prostatic Dis..

[11] Ivanov S., Liao S.-Y., Ivanova A., Danilkovitch-Miagkova A., Tarasova N., Weirich G., Merrill M.J., Proescholdt M.A., Oldfield E.H., Lee J. (2001). Expression of Hypoxia-Inducible Cell-Surface Transmembrane Carbonic Anhydrases in Human Cancer. Am. J. Pathol..

[12] Chiche J., Ilc K., Laferrie`re J., Trottier E., Dayan F., Mazure N.M., Brahimi-Horn M.C., Pouysségur J. (2009). Hypoxia-Inducible Carbonic Anhydrase IX and XII Promote Tumor Cell Growth by Counteracting Acidosis through the Regulation of the Intracellular pH. Cancer Res..

[13] 13 Pastorekova S., Gillies R.J. (2019). The role of carbonic anhydrase IX in cancer development: Links to hypoxia, acidosis, and beyond. Cancer Metastasis Rev..

[14] Mboge M.Y., McKenna R., Frost S.C. (2015). Advances in Anti-Cancer Drug Development Targeting Carbonic Anhydrase IX and XII. Top. Anticancer Res..

[15] Kirsh S.M., Pascetta S.A., Uniacke J., Ursini-Siegel J. (2023). Spheroids as a 3D Model of the Hypoxic Tumor Microenvironment. The Tumor Microenvironment.

[16] Riffle S., Hegde R.S. (2017). Modeling Tumor Cell Adaptations to Hypoxia in Multicellular Tumor Spheroids. J. Exp. Clin. Cancer Res..

[17] Sulfanilamide|Drug|Britannica. https://www.britannica.com/science/sulfanilamide.

[18] Gaffer H.E., Mahmoud S.A., El-Sedik M.S., Aysha T., Abdel-Rhman M.H., Abdel-latif E. (2024). Synthesis, Molecular Modelling, and Antibacterial Evaluation of New Sulfonamide-Dyes Based Pyrrole Compounds. Sci. Rep..

[19] Abd El Salam H.A., Abdel-Aziz M.S., El-Sawy E.R., Shaban E. (2023). Synthesis and Antibacterial Activity of Azo-Sulfa-Based Disperse Dyes and Their Application in Polyester Printing. Fibers Polym..

[20] Bringmann G., Dreyer M., Faber J.H., Dalsgaard P.W., Stærk D., Jaroszewski J.W., Ndangalasi H., Mbago F., Brun R., Christensen S.B. (2004). Ancistrotanzanine C and Related 5,1′- and 7,3′-Coupled Naphthylisoquinoline Alkaloids from *Ancistrocladus tanzaniensis*. J. Nat. Prod..

[21] Souza A.O.D., Galetti F.C.S., Silva C.L., Bicalho B., Parma M.M., Fonseca S.F., Marsaioli A.J., Trindade A.C.L.B., Gil R.P.F., Bezerra F.S. (2007). Antimycobacterial and Cytotoxicity Activity of Synthetic and Natural Compounds. Quím. Nova.

[22] Raczuk E., Dmochowska B., Samaszko-Fiertek J., Madaj J. (2022). Different Schiff Bases—Structure, Importance and Classification. Molecules.

[23] Ceramella J., Iacopetta D., Catalano A., Cirillo F., Lappano R., Sinicropi M.S. (2022). A Review on the Antimicrobial Activity of Schiff Bases: Data Collection and Recent Studies. Antibiotics.

[24] Rehman W., Baloch M.K., Muhammad B., Badshah A., Khan K.M. (2004). Characteristic Spectral Studies Andin Vitro Antifungal Activity of Some Schiff Bases and Their Organotin (IV) Complexes. Chin. Sci. Bull..

[25] Karthikeyan M.S., Prasad D.J., Poojary B., Subrahmanya Bhat K., Holla B.S., Kumari N.S. (2006). Synthesis and Biological Activity of Schiff and Mannich Bases Bearing 2,4-Dichloro-5-Fluorophenyl Moiety. Bioorg. Med. Chem..

[26] Kayser O., Kiderlen A.F., Croft S.L. (2003). Natural Products as Antiparasitic Drugs. Parasitol. Res..

[27] Przybylski P., Huczynski A., Pyta K., Brzezinski B., Bartl F. (2009). Biological Properties of Schiff Bases and Azo Derivatives of Phenols. Curr. Org. Chem..

[28] Murtaza S., Akhtar M.S., Kanwal F., Abbas A., Ashiq S., Shamim S. (2017). Synthesis and Biological Evaluation of Schiff Bases of 4-Aminophenazone as an Anti-Inflammatory, Analgesic and Antipyretic Agent. J. Saud. Chem. Soc..

[29] Gujjarappa R., Kabi A.K., Sravani S., Garg A., Vodnala N., Tyagi U., Kaldhi D., Velayutham R., Singh V., Gupta S., Swain B.P. (2022). Overview on Biological Activities of Imidazole Derivatives. Nanostructured Biomaterials.

[30] Luca L. (2006). Naturally Occurring and Synthetic Imidazoles: Their Chemistry and Their Biological Activities. Curr. Med. Chem..

[31] Rashid M., Maqbool A., Shafiq N., Bin Jardan Y.A., Parveen S., Bourhia M., Nafidi H.-A., Khan R.A. (2023). The Combination of Multi-Approach Studies to Explore the Potential Therapeutic Mechanisms of Imidazole Derivatives as an MCF-7 Inhibitor in Therapeutic Strategies. Front. Chem..

[32] Balandis B., Mickevičius V., Petrikaitė V. (2021). Exploration of Benzenesulfonamide-Bearing Imidazole Derivatives Activity in Triple-Negative Breast Cancer and Melanoma 2D and 3D Cell Cultures. Pharmaceuticals.

[33] Holzer W., Hahn K. (2003). Synthesis of Substituted 3-phenyl-6 *h* -pyrazolo[4,3-d]Isoxazoles from Corresponding 4-benzoyl-5-hydroxypyrazoles. J. Heterocycl. Chem..

[34] Chourasiya S.S., Kathuria D., Wani A.A., Bharatam P.V. (2019). Azines: Synthesis, Structure, Electronic Structure and Their Applications. Org. Biomol. Chem..

[35] DeLeeuw L.W., Monsen R.C., Petrauskas V., Gray R.D., Baranauskiene L., Matulis D., Trent J.O., Chaires J.B. (2021). POT1 Stability and Binding Measured by Fluorescence Thermal Shift Assays. PLoS ONE.

[36] Gedgaudas M., Baronas D., Kazlauskas E., Petrauskas V., Matulis D. (2022). Thermott: A Comprehensive Online Tool for Protein–Ligand Binding Constant Determination. Drug Discov. Today.

[37] Matulienė J., Žvinys G., Petrauskas V., Kvietkauskaitė A., Zakšauskas A., Shubin K., Zubrienė A., Baranauskienė L., Kačenauskaitė L., Kopanchuk S. (2022). Picomolar Fluorescent Probes for Compound Affinity Determination to Carbonic Anhydrase IX Expressed in Live Cancer Cells. Sci. Rep..

[38] Cherinka B., Andrews B.H., Sánchez-Gallego J., Brownstein J., Argudo-Fernández M., Blanton M., Bundy K., Jones A., Masters K., Law D.R. (2019). Marvin: A Tool Kit for Streamlined Access and Visualization of the SDSS-IV MaNGA Data Set. Astron. J..

[39] Alterio V., Di Fiore A., D’Ambrosio K., Supuran C.T., De Simone G. (2012). Multiple Binding Modes of Inhibitors to Carbonic Anhydrases: How to Design Specific Drugs Targeting 15 Different Isoforms?. Chem. Rev..

[40] Li L., Lu Y. (2011). Optimizing a 3D Culture System to Study the Interaction between Epithelial Breast Cancer and Its Surrounding Fibroblasts. J. Cancer.

[41] Roque Marques K.M., Do Desterro M.R., De Arruda S.M., De Araújo Neto L.N., Do Carmo Alves De Lima M., De Almeida S.M.V., Da Silva E.C.D., De Aquino T.M., Da Silva-Júnior E.F., De Araújo-Júnior J.X. (2019). 5-Nitro-Thiophene-Thiosemicarbazone Derivatives Present Antitumor Activity Mediated by Apoptosis and DNA Intercalation. Curr. Top. Med. Chem..

[42] Daunys S., Petrikaitė V. (2020). The Roles of Carbonic Anhydrases IX and XII in Cancer Cell Adhesion, Migration, Invasion and Metastasis. Biol. Cell.

[43] Bonardi A., Supuran C.T. (2025). Polypharmacology of Carbonic Anhydrase Inhibitors and Activators. Expert. Opin. Pharmacother..

[44] Ovung A., Bhattacharyya J. (2021). Sulfonamide Drugs: Structure, Antibacterial Property, Toxicity, and Biophysical Interactions. Biophys. Rev..

[45] Freake H.C., Sankavaram K. (2013). Zinc: Physiology, Dietary Sources, and Requirements. Encyclopedia of Human Nutrition.

[46] Oh S., Moon H., Son I., Jung J. (2007). Synthesis of Sulfonamides and Evaluation of Their Histone Deacetylase (HDAC) Activity. Molecules.

[47] Luchinat E., Barbieri L., Cremonini M., Nocentini A., Supuran C.T., Banci L. (2020). Intracellular Binding/Unbinding Kinetics of Approved Drugs to Carbonic Anhydrase II Observed by in-Cell NMR. ACS Chem. Biol..

[48] Dudutienė V., Matulienė J., Smirnov A., Timm D.D., Zubrienė A., Baranauskienė L., Morkunaite V., Smirnovienė J., Michailovienė V., Juozapaitienė V. (2014). Discovery and Characterization of Novel Selective Inhibitors of Carbonic Anhydrase IX. J. Med. Chem..

[49] Mickevičiūtė A., Juozapaitienė V., Michailovienė V., Jachno J., Matulienė J., Matulis D., Matulis D. (2019). Recombinant Production of 12 Catalytically Active Human CA Isoforms. Carbonic Anhydrase as Drug Target.

[50] Kabsch W. (2010). *XDS*. Acta Crystallogr. D Biol. Crystallogr..

[51] Agirre J., Atanasova M., Bagdonas H., Ballard C.B., Baslé A., Beilsten-Edmands J., Borges R.J., Brown D.G., Burgos-Mármol J.J., Berrisford J.M. (2023). The CCP 4 Suite: Integrative Software for Macromolecular Crystallography. Acta Crystallogr. D Struct. Biol..

[52] Vagin A., Teplyakov A. (2010). Molecular Replacement with *MOLREP*. Acta Crystallogr. D Biol. Crystallogr..

[53] Murshudov G.N., Skubák P., Lebedev A.A., Pannu N.S., Steiner R.A., Nicholls R.A., Winn M.D., Long F., Vagin A.A. (2011). *REFMAC* 5 for the Refinement of Macromolecular Crystal Structures. Acta Crystallogr. D Biol. Crystallogr..

[54] Emsley P., Lohkamp B., Scott W.G., Cowtan K. (2010). Features and Development of *Coot*. Acta Crystallogr. D Biol. Crystallogr..

[55] Hanwell M.D., Curtis D.E., Lonie D.C., Vandermeersch T., Zurek E., Hutchison G.R. (2012). Avogadro: An Advanced Semantic Chemical Editor, Visualization, and Analysis Platform. J. Cheminform..

[56] Skaraitė I., Maccioni E., Petrikaitė V. (2023). Anticancer Activity of Sunitinib Analogues in Human Pancreatic Cancer Cell Cultures under Normoxia and Hypoxia. Int. J. Mol. Sci..

