# Supplementary material for: Novel Derivatives of 3-Amino-4-hydroxy-benzenesulfonamide: Synthesis, Binding to Carbonic Anhydrases, and Activity in Cancer Cell 2D and 3D Cultures
Source: Int J Mol Sci. 2025 Jul 4;26(13):6466. doi: 10.3390/ijms26136466 (PMC12249541; doi:10.3390/ijms26136466)
Supplement: Supplementary file 1 [file ijms-26-06466-s001.zip › ijms-3709128-supplementary.pdf]

Supplementary Materials

Novel derivatives of 3-amino-4-hydroxy-benzenesulfonamide: synthesis, binding to carbonic anhydrases, and activity in cancer cell 2D and 3D cultures

Valdas Vainauskas[1], Rugilė Norvaišaitė[2], Birutė Grybaitė[1], Rita Vaickelionienė[1], Alexey Smirnov[2], Tautvydas Kojis[2], Lina Baranauskiene[2], Elena Manakova[3], Saulius Gražulis[4], Asta Zubrienė[2], Daumantas Matulis[2], Vytautas Mickevičius[1],Vilma Petrikaitė[5,6*]

1 Department of Organic Chemistry, Kaunas University of Technology, Radvilėnų Rd. 19, LT-50254 Kaunas, Lithuania; karolina.kairyte@ktu.edu (K.K.); birute.grybaite@ktu.lt (B.G.); rita.vaickelioniene@ktu.lt (R.V.); birute.sapijanskaite@ktu.lt (B.S.-B); vytautas.mickevicius@ktu.lt (V.M.)

2 Department of Biothermodynamics and Drug Design, Institute of Biotechnology, Life Sciences Center, Vilnius University, Saulėtekio 7, LT-10257 Vilnius, Lithuania; rugile117@gmail.com (R.N.); alexey.smirnov@bti.vu.lt (A.S.); tautvydas.kojis@gmc.vu.lt (T.K.); lina.baranauskiene@bti.vu.lt (L.B.) daumantas.matulis@bti.vu.lt (D.M.); asta.zubriene@bti.vu.lt (A.Z.)

3 Department of Protein–DNA Interactions, Institute of Biotechnology, Life Sciences Center, Vilnius University, Saulėtekio al. 7, LT-10257 Vilnius, Lithuania; elena.manakova@bti.vu.lt (E.M.)

4 Sector of Crystallography and Chemical Informatics Institute of Biotechnology, Life Sciences Center, Vilnius University, Saulėtekio al. 7, LT-10257 Vilnius, Lithuania; saulius.grazulis@bti.vu.lt (S.G.)

5 Institute of Biotechnology, Life Sciences Center, Vilnius University, Saulėtekio 7, LT-10257 Vilnius, Lithuania; vilma.petrikaite@bti.vu.lt (V.P.)

6 Laboratory of Drug Targets Histopathology, Institute of Cardiology, Lithuanian University of Health Sciences, Sukilėlių 13, LT-50162 Kaunas, Lithuania; vilma.petrikaite@lsmu.lt (V.P.)

* Correspondence: vilma.petrikaite@lsmu.lt, vytautas.mickevicius@ktu.lt

NMR Spectra (compounds 2–38, all in DMSO-d_6, Figures S1–S68)
Mass spectrum of compound 18 (Figure S69)

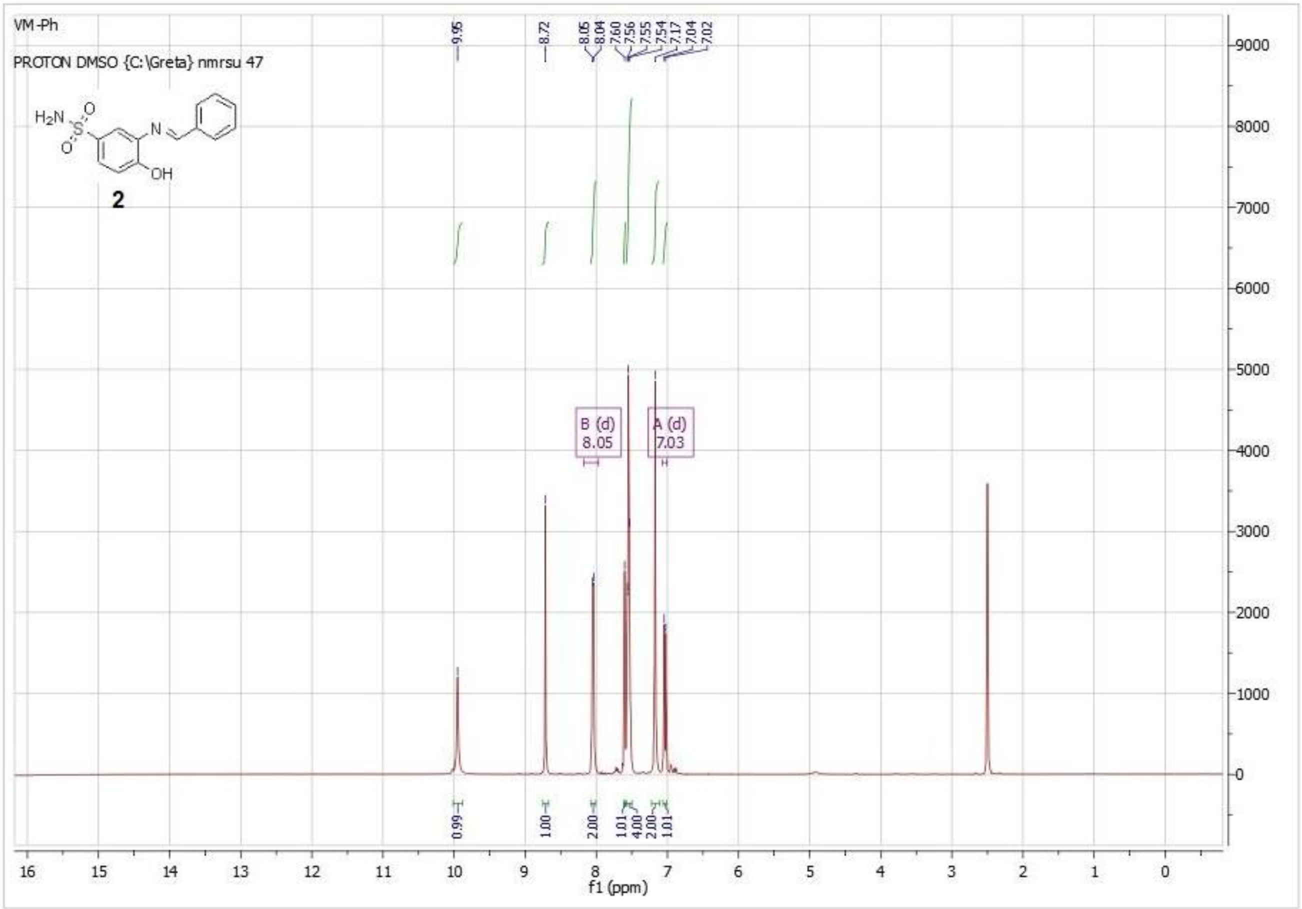

Figure S1. ^{1}H NMR of compound **2.**

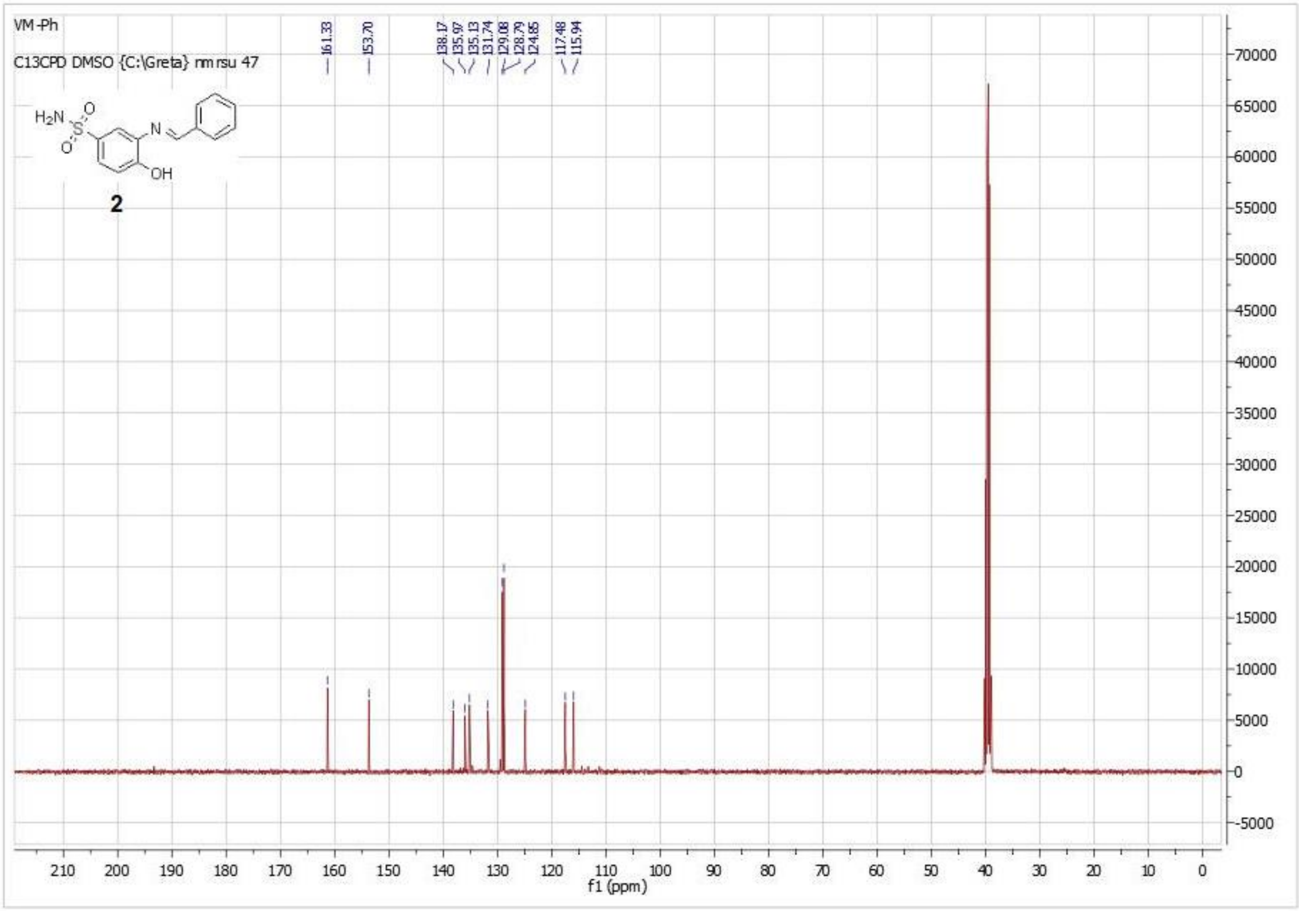

Figure S2. ^{13}C NMR of compound **2.**

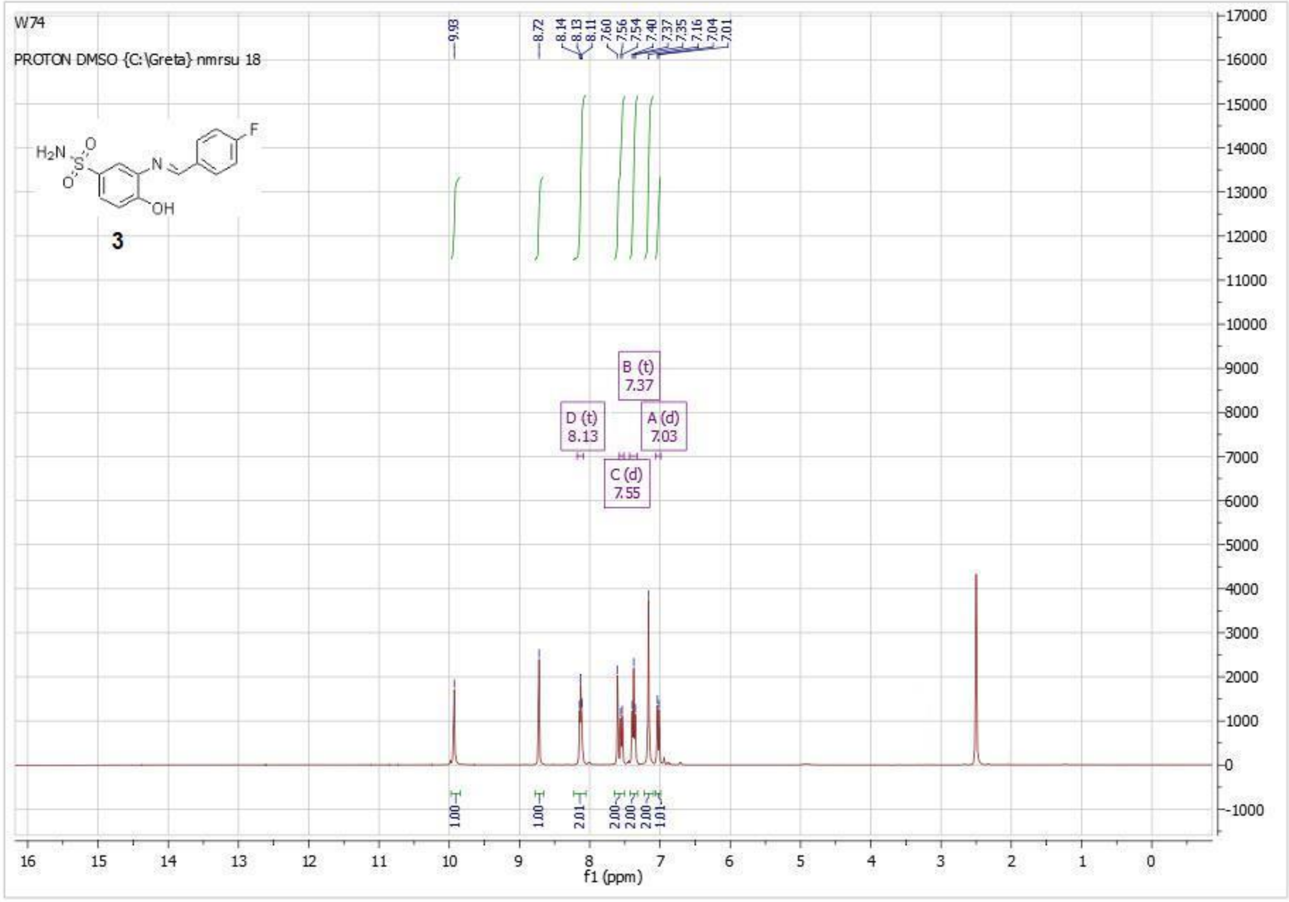

Figure S3. ^{1}H NMR of compound **3.**

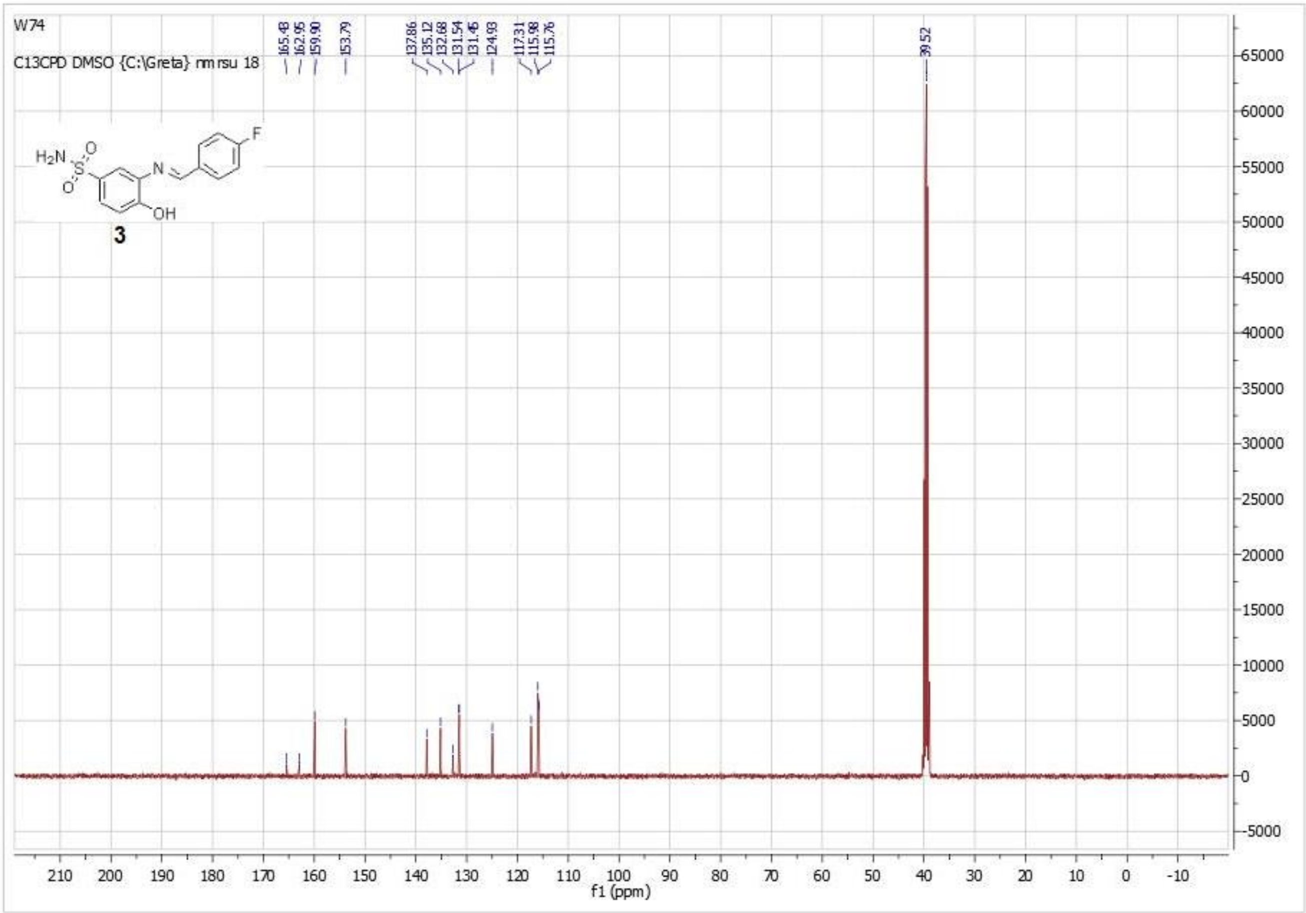

Figure S4. ^{13}C NMR of compound **3.**

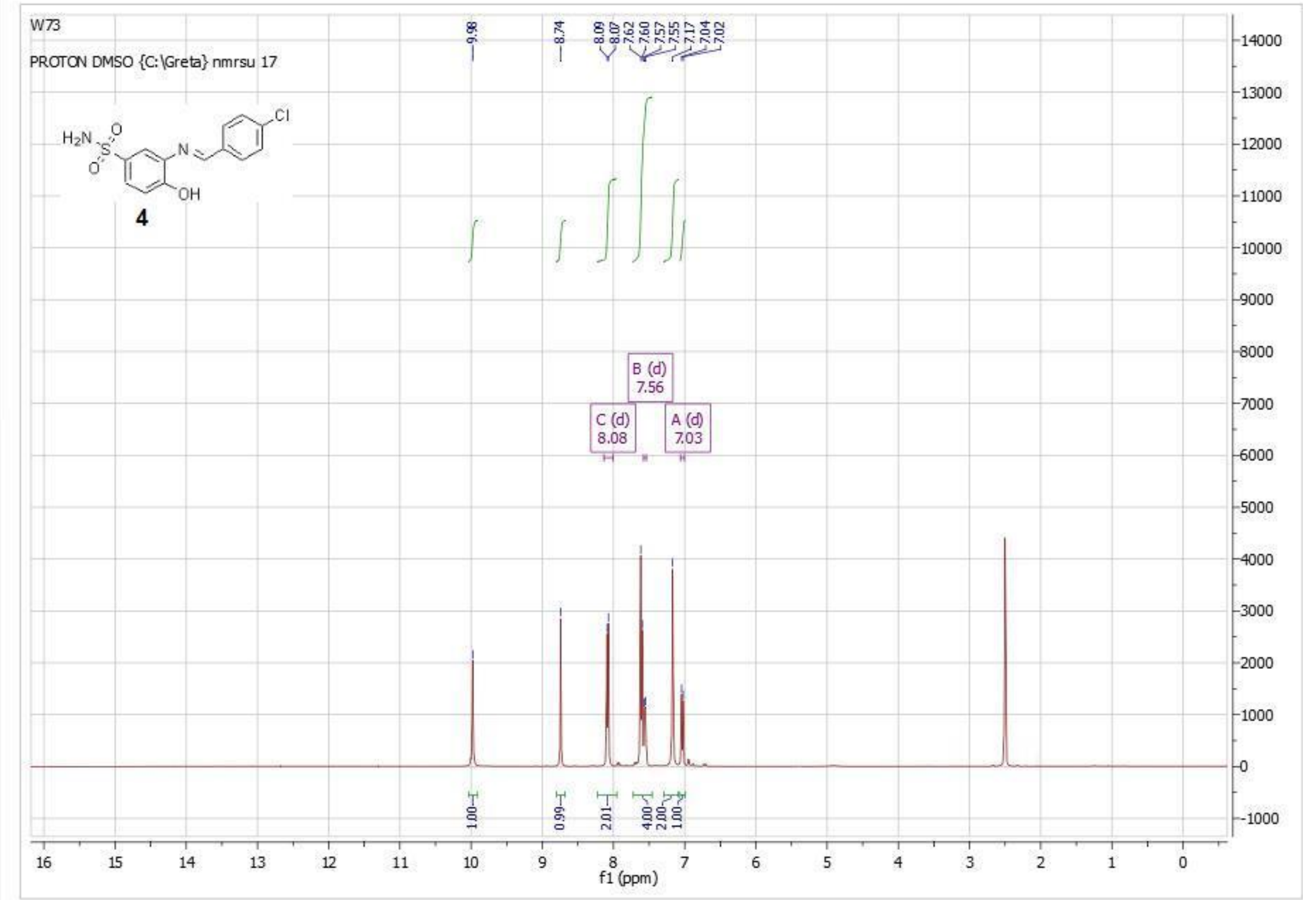

Figure S5. ^{1}H NMR of compound **4.**

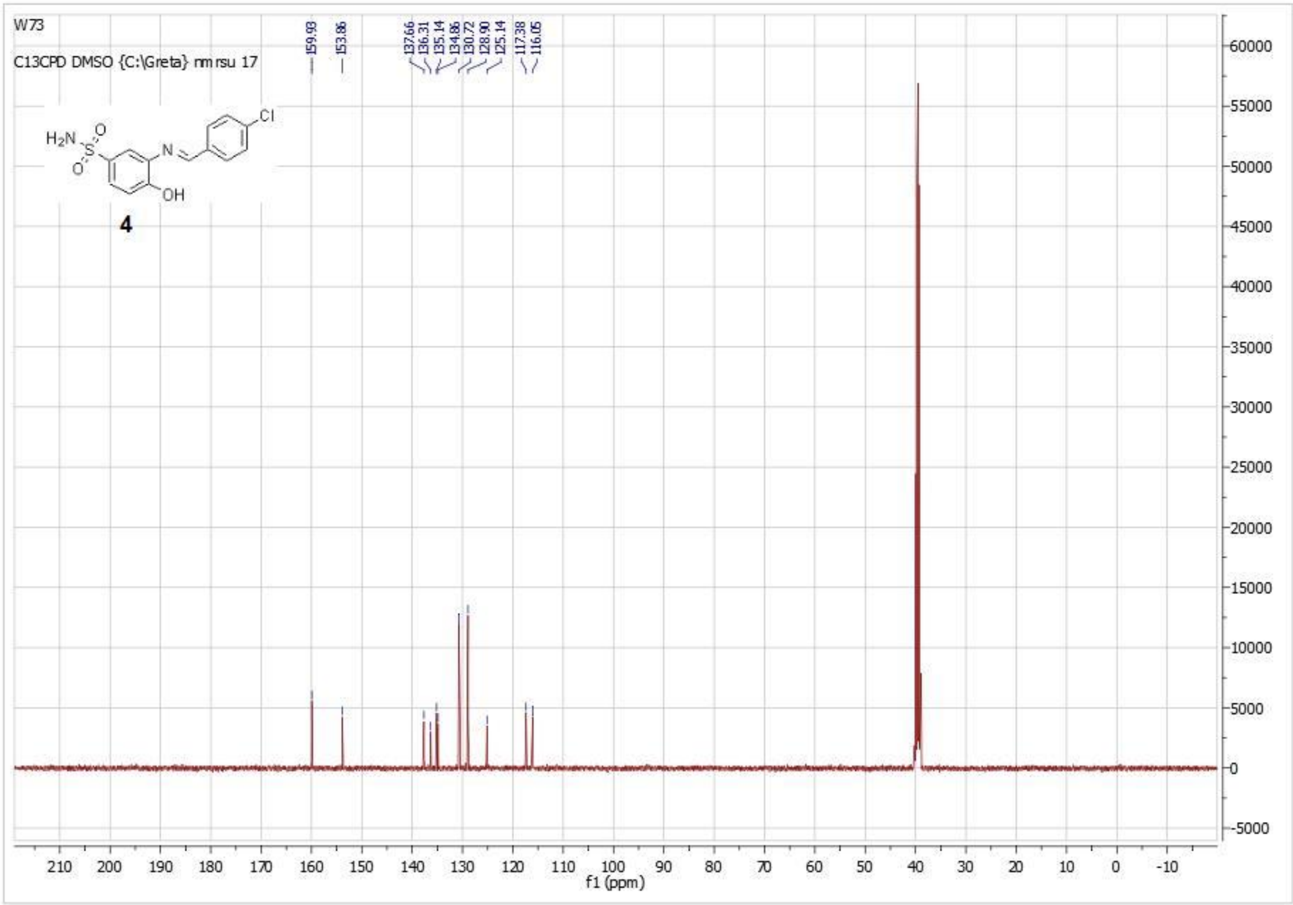

Figure S6. ^{1}H NMR of compound **4.**

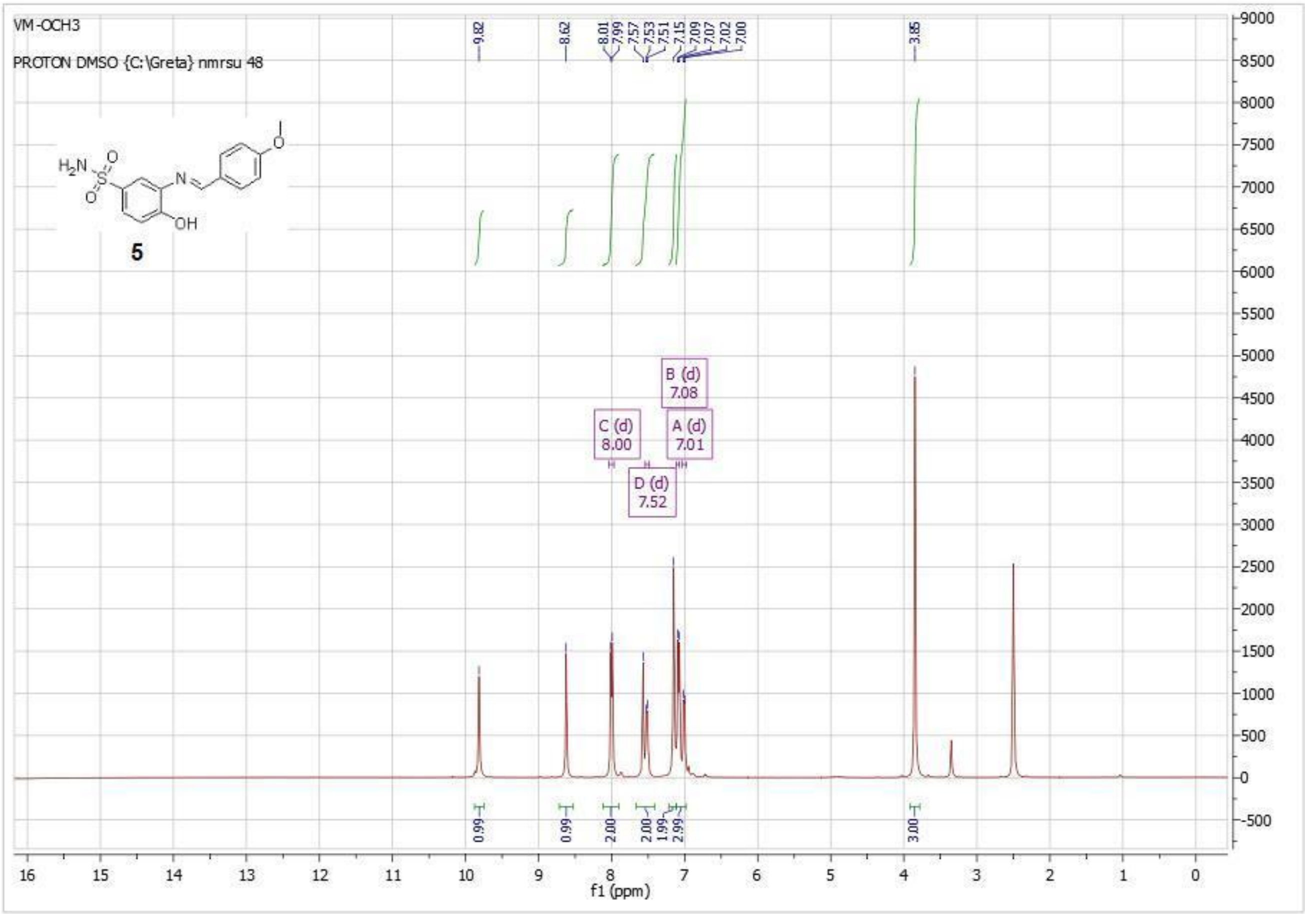

Figure S7. ^{1}H NMR of compound **5.**

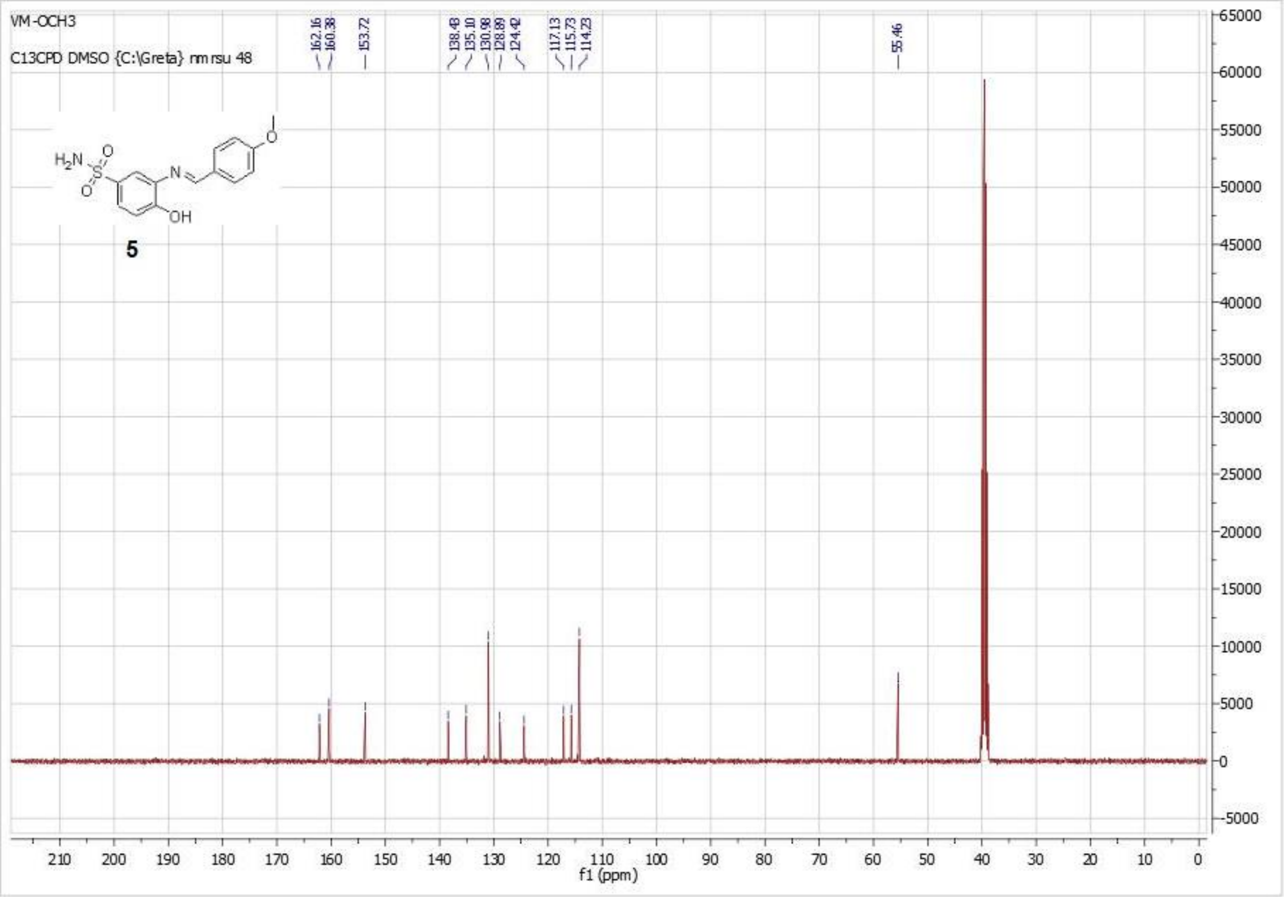

Figure S8. ^{1}H NMR of compound **5.**

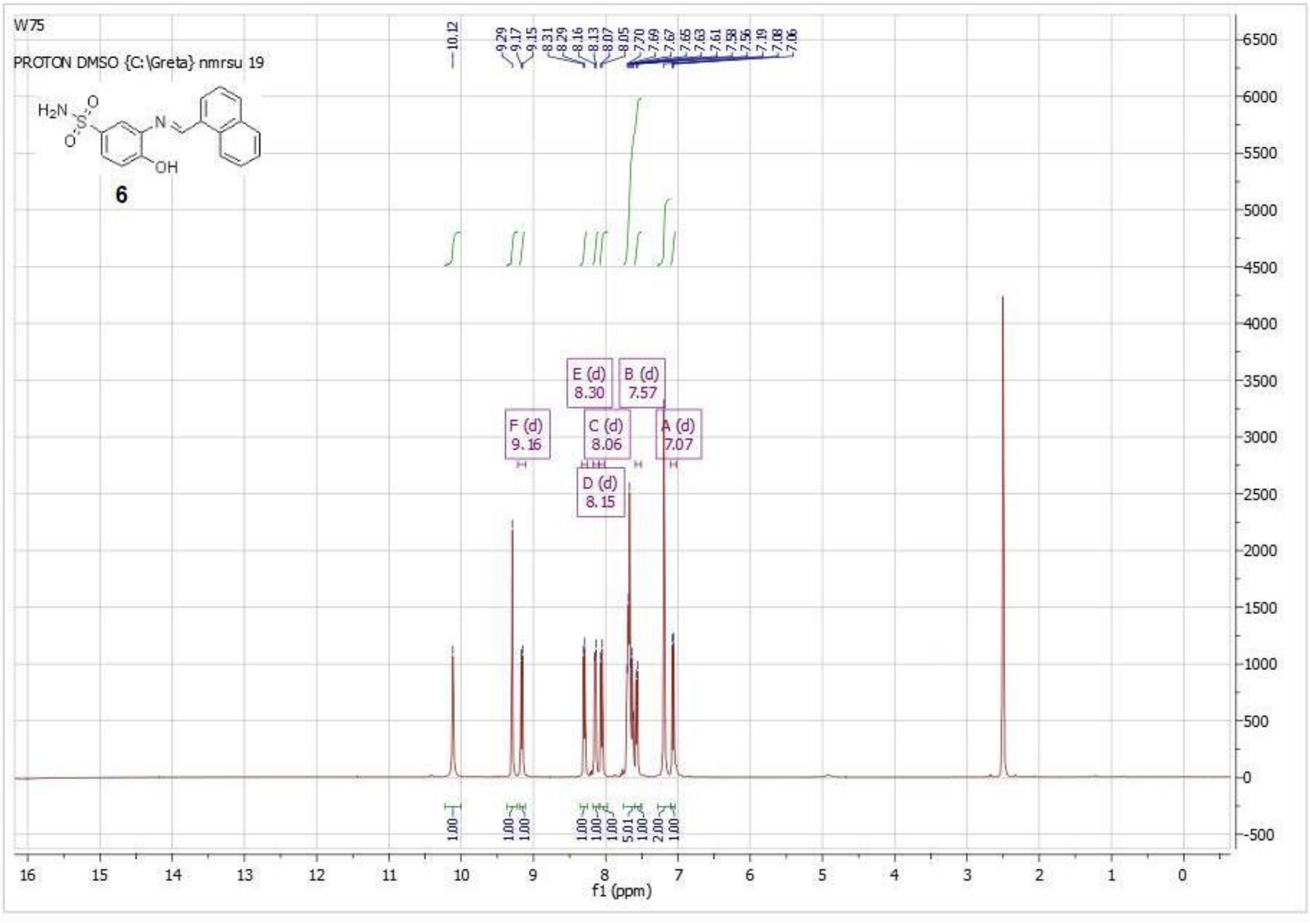

Figure S9. ^{1}H NMR of compound **6.**

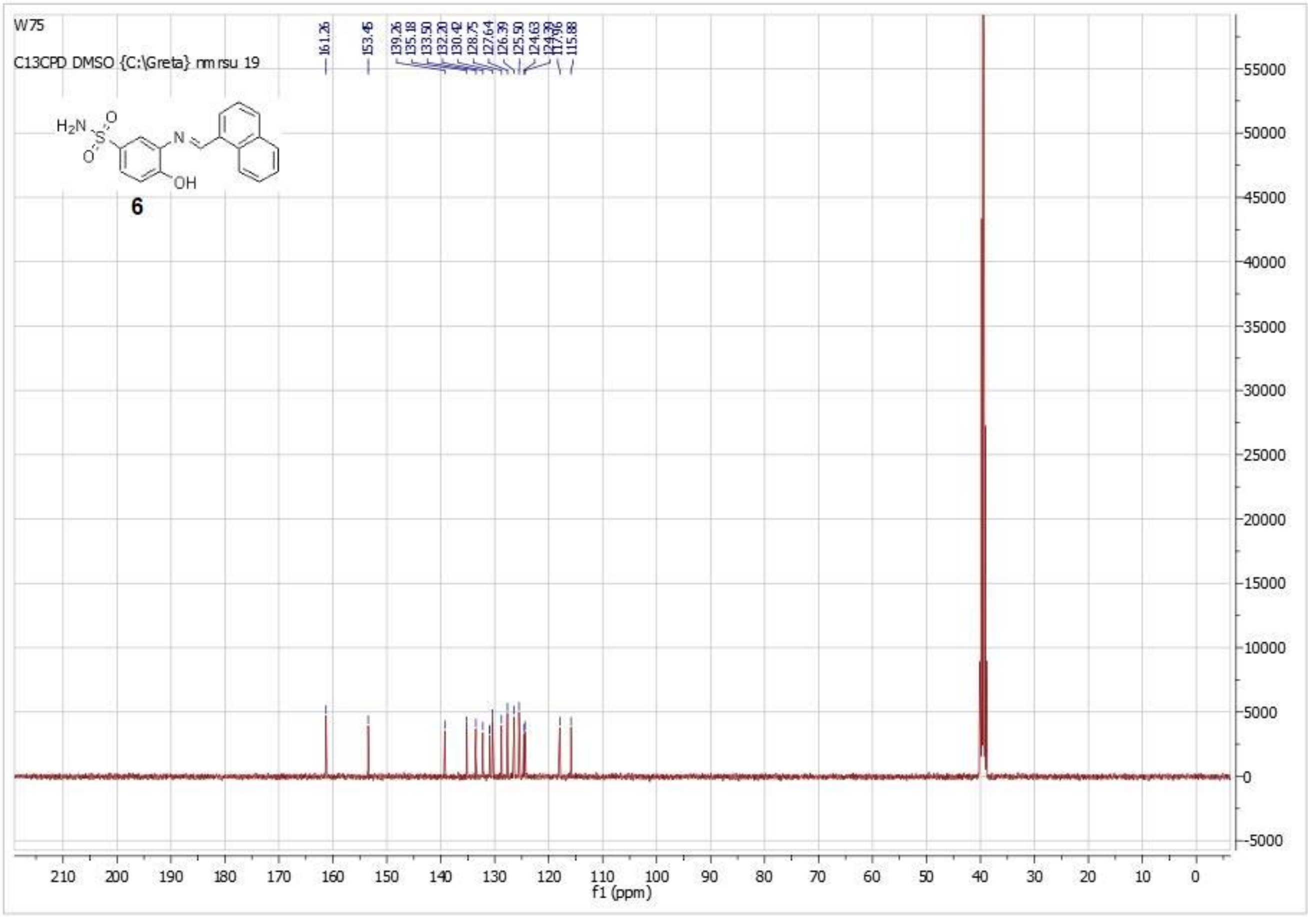

Figure S10. ^{13}C NMR of compound **6.**

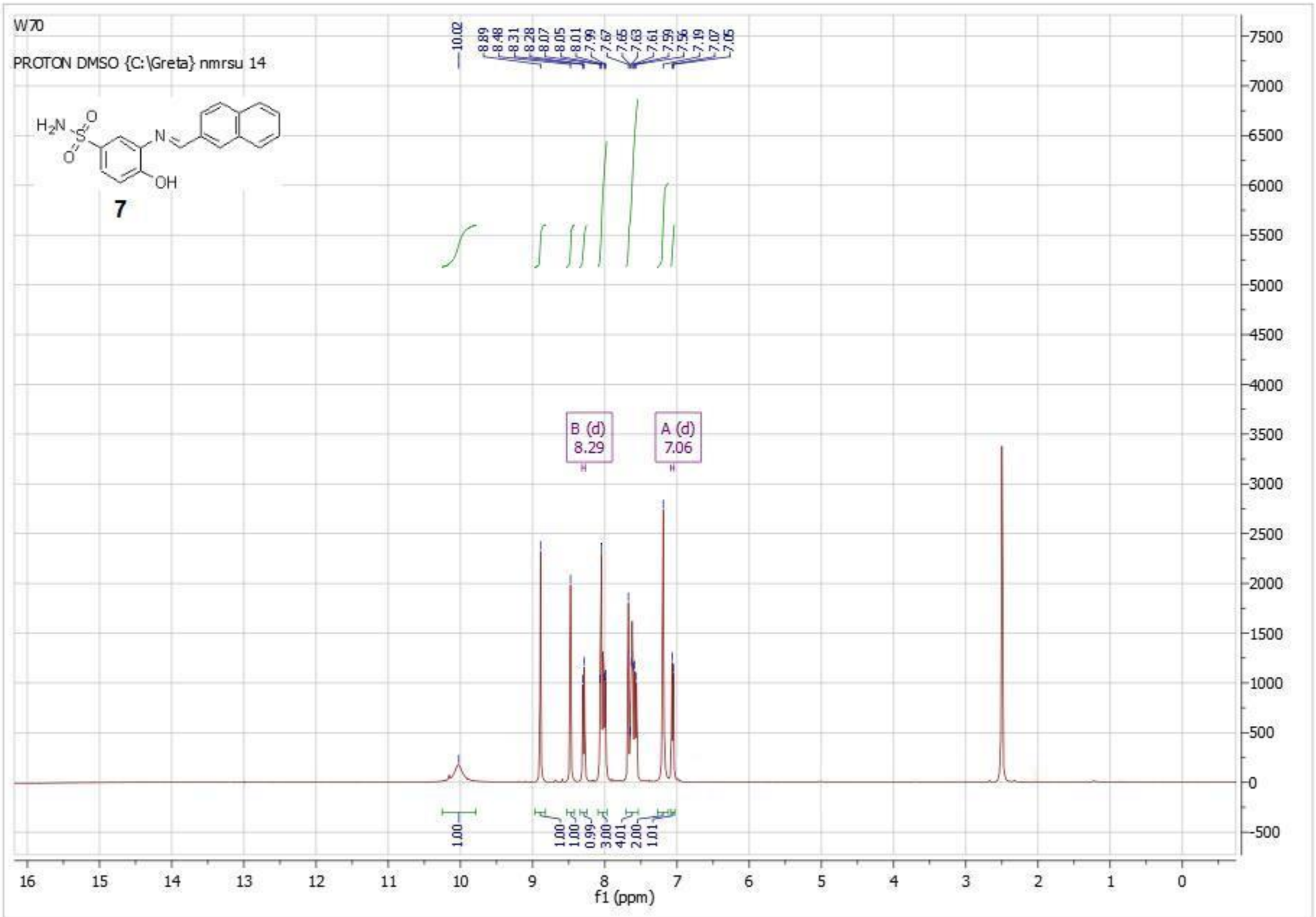

Figure S11. ^{1}H NMR of compound **7.**

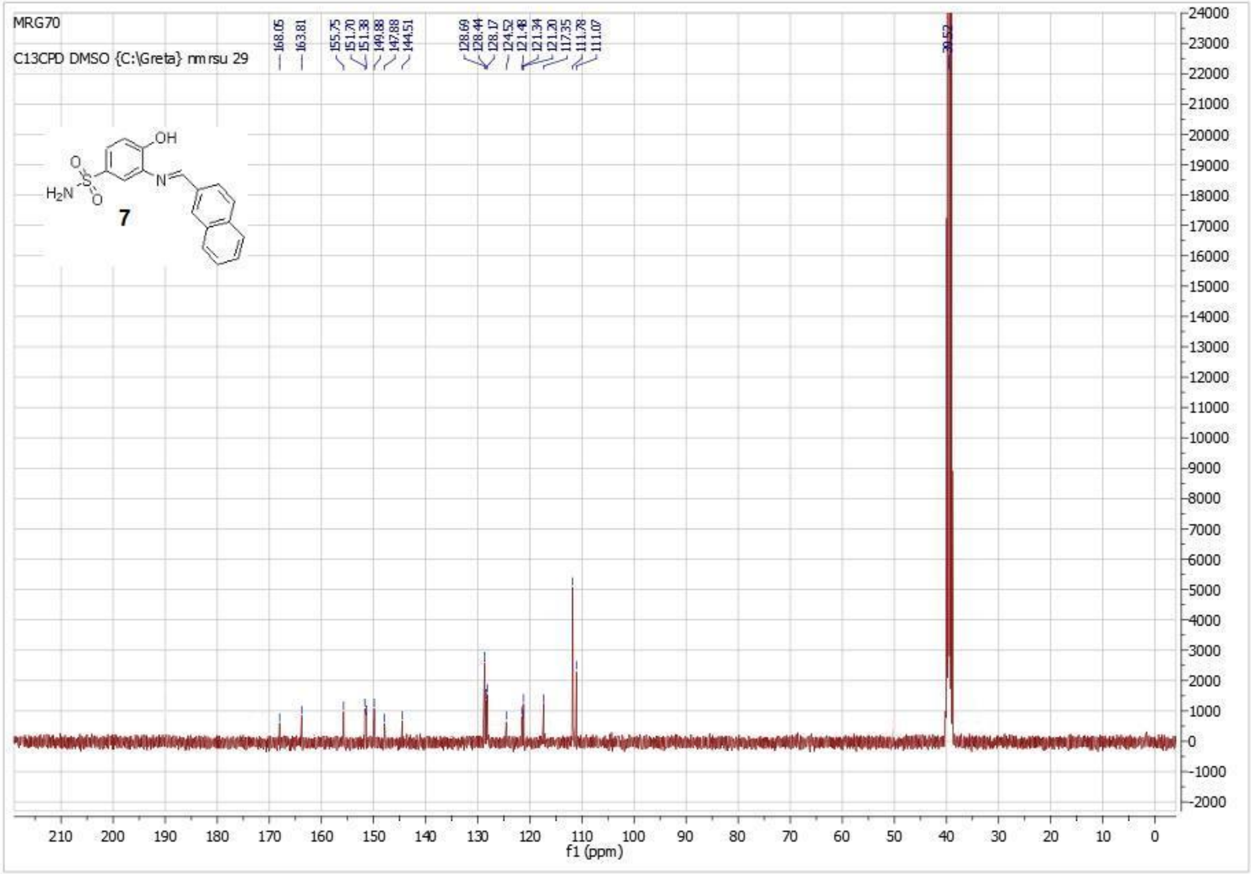

Figure S12. ^{13}C NMR of compound **7.**

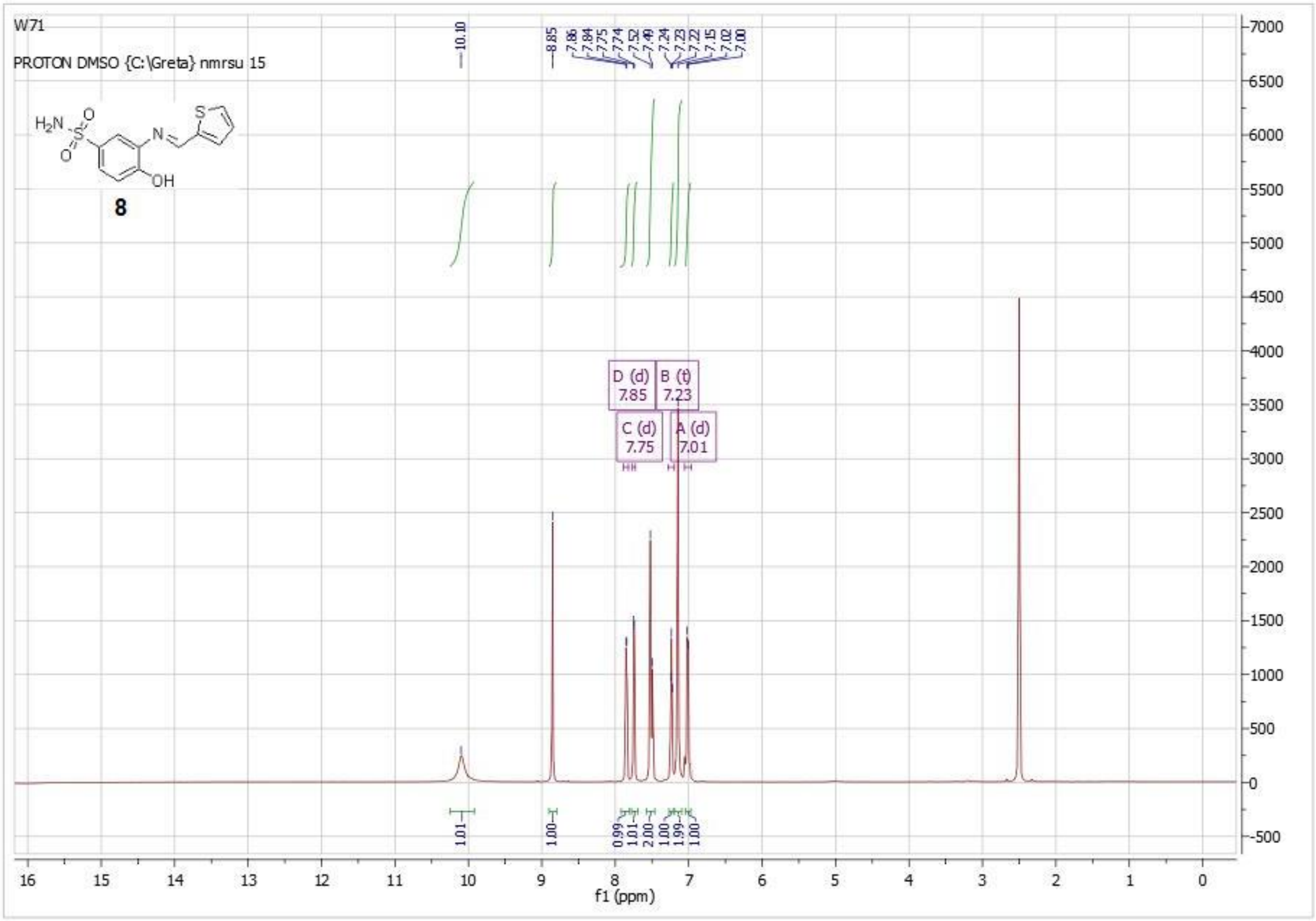

Figure S13. ^{1}H NMR of compound **8.**

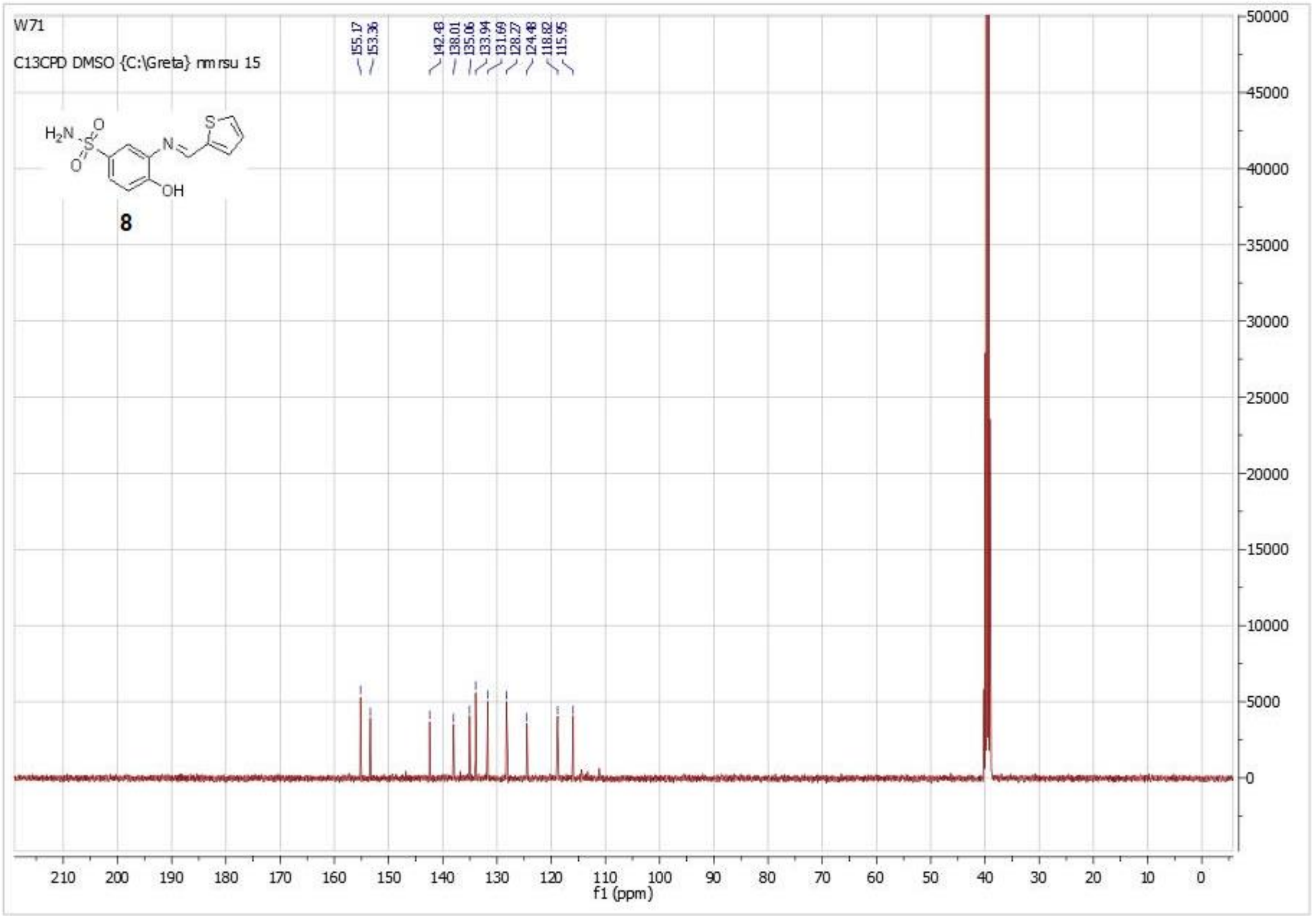

Figure S14. ^{13}C NMR of compound **8.**

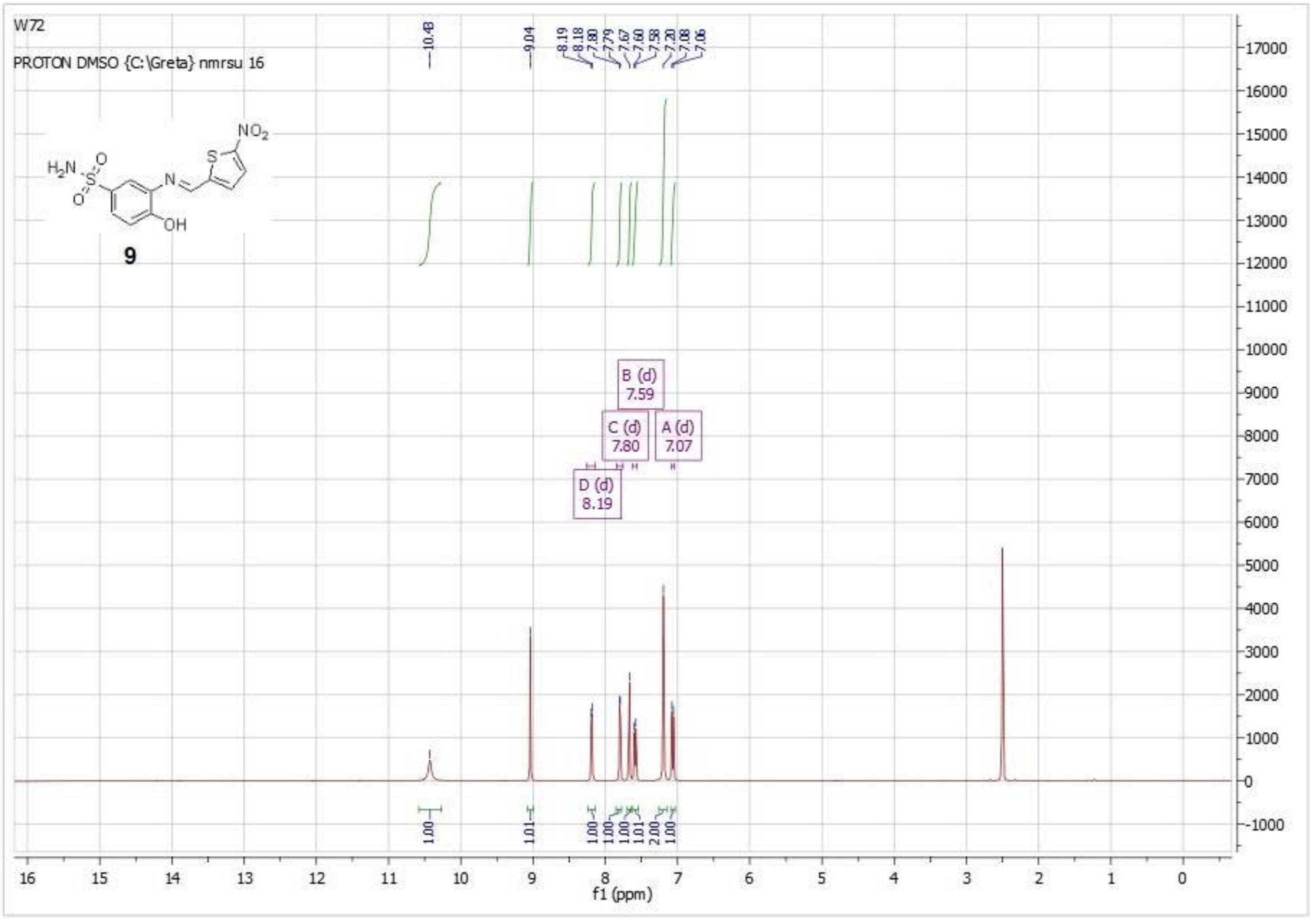

Figure S15. ^{1}H NMR of compound **9.**

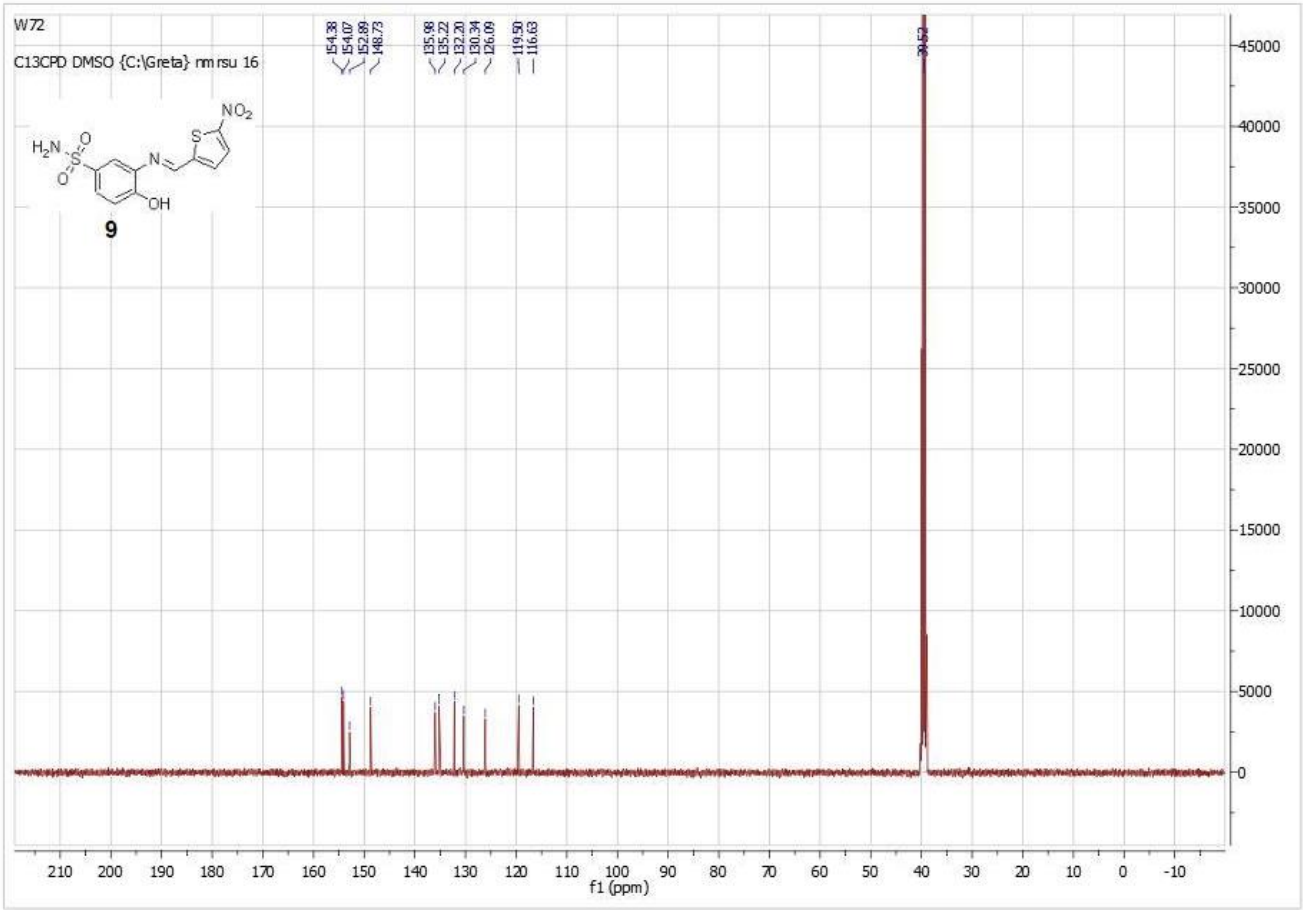

Figure S16. ^{13}C NMR of compound **9.**

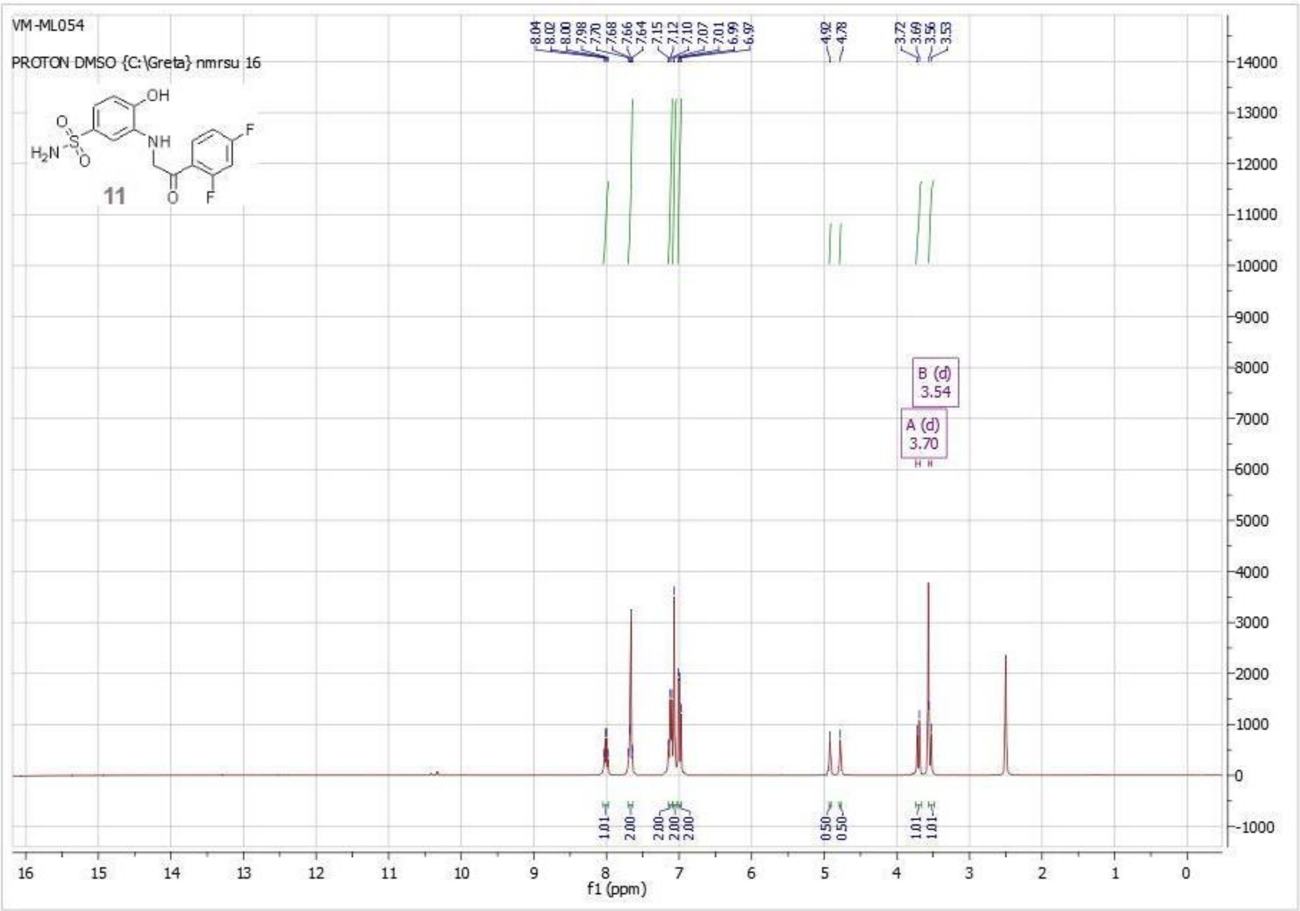

Figure S17. ^{1}H NMR of compound **11.**

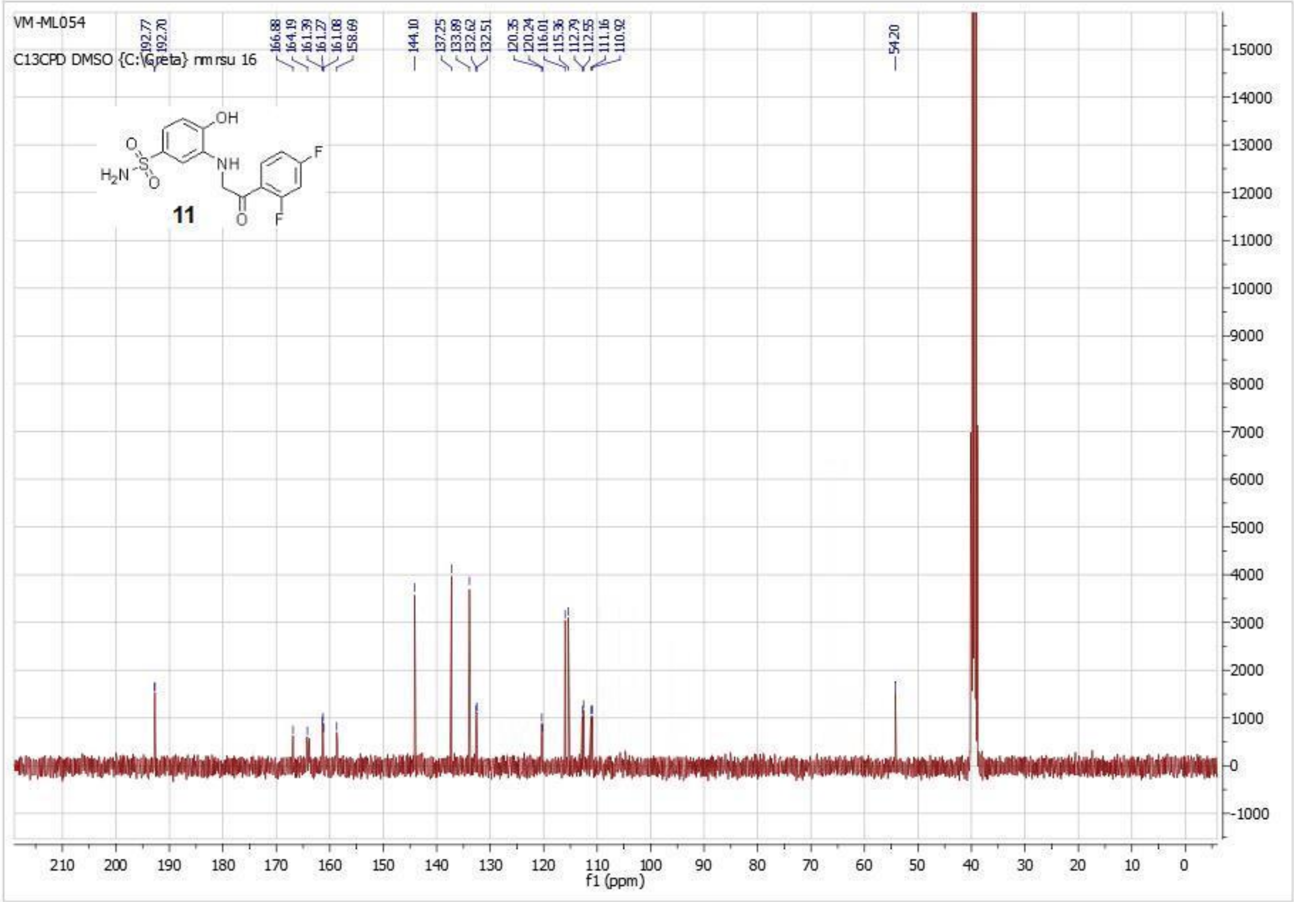

Figure S18. ^{13}C NMR of compound **11.**

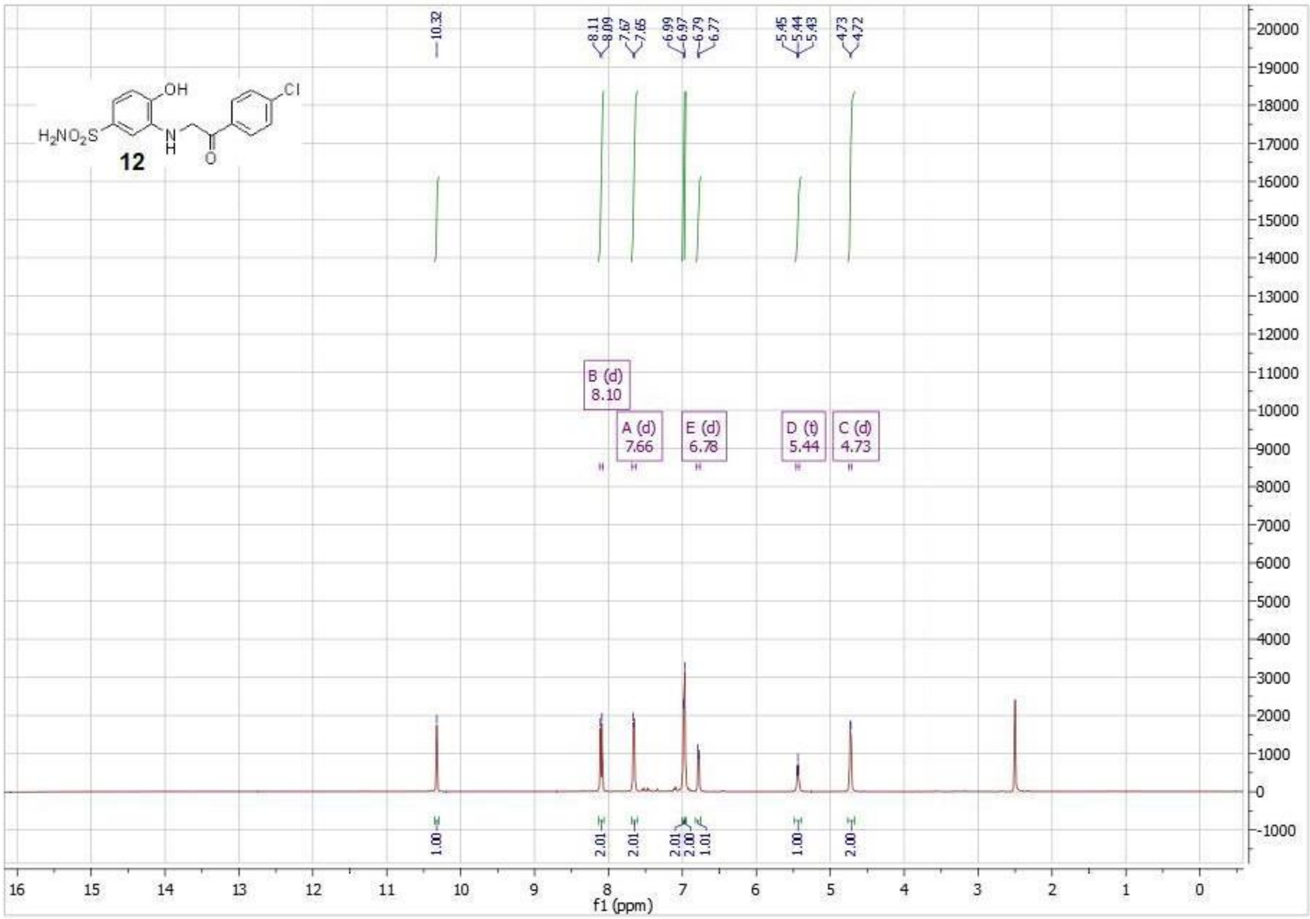

Figure S19. ^{1}H NMR of compound **12.**

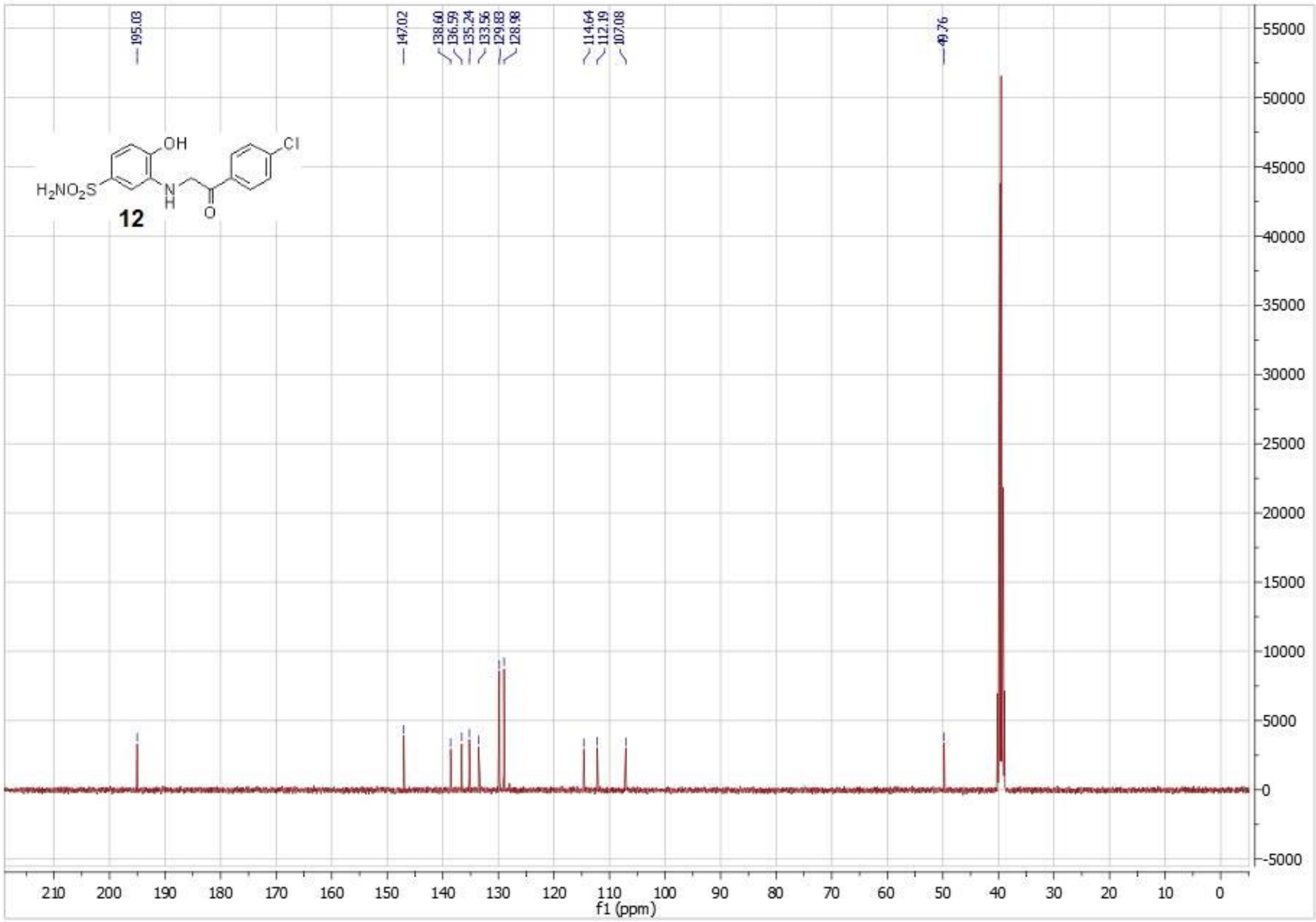

Figure S20. ^{13}C NMR of compound **12.**

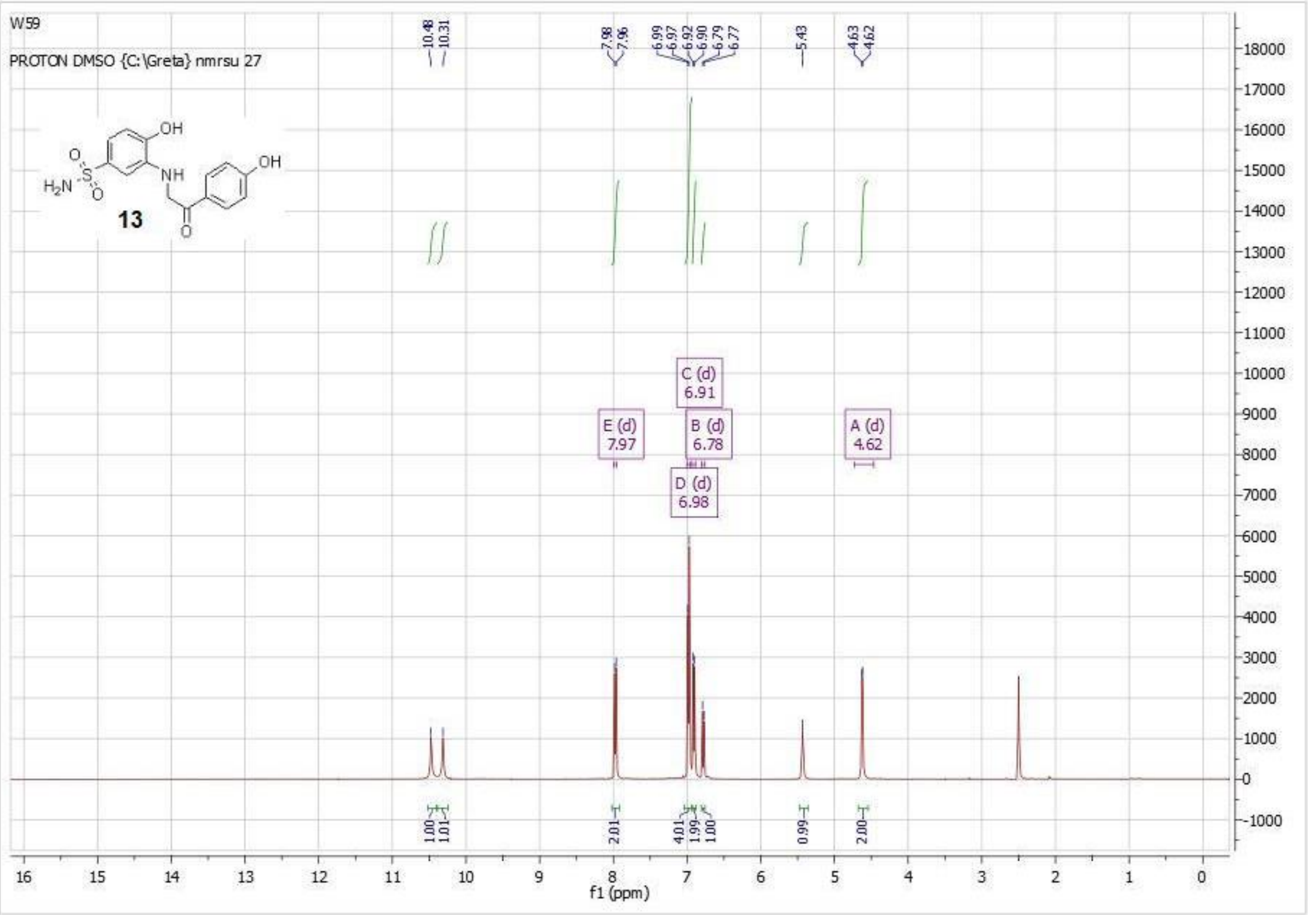

Figure S21. ^{1}H NMR of compound **13.**

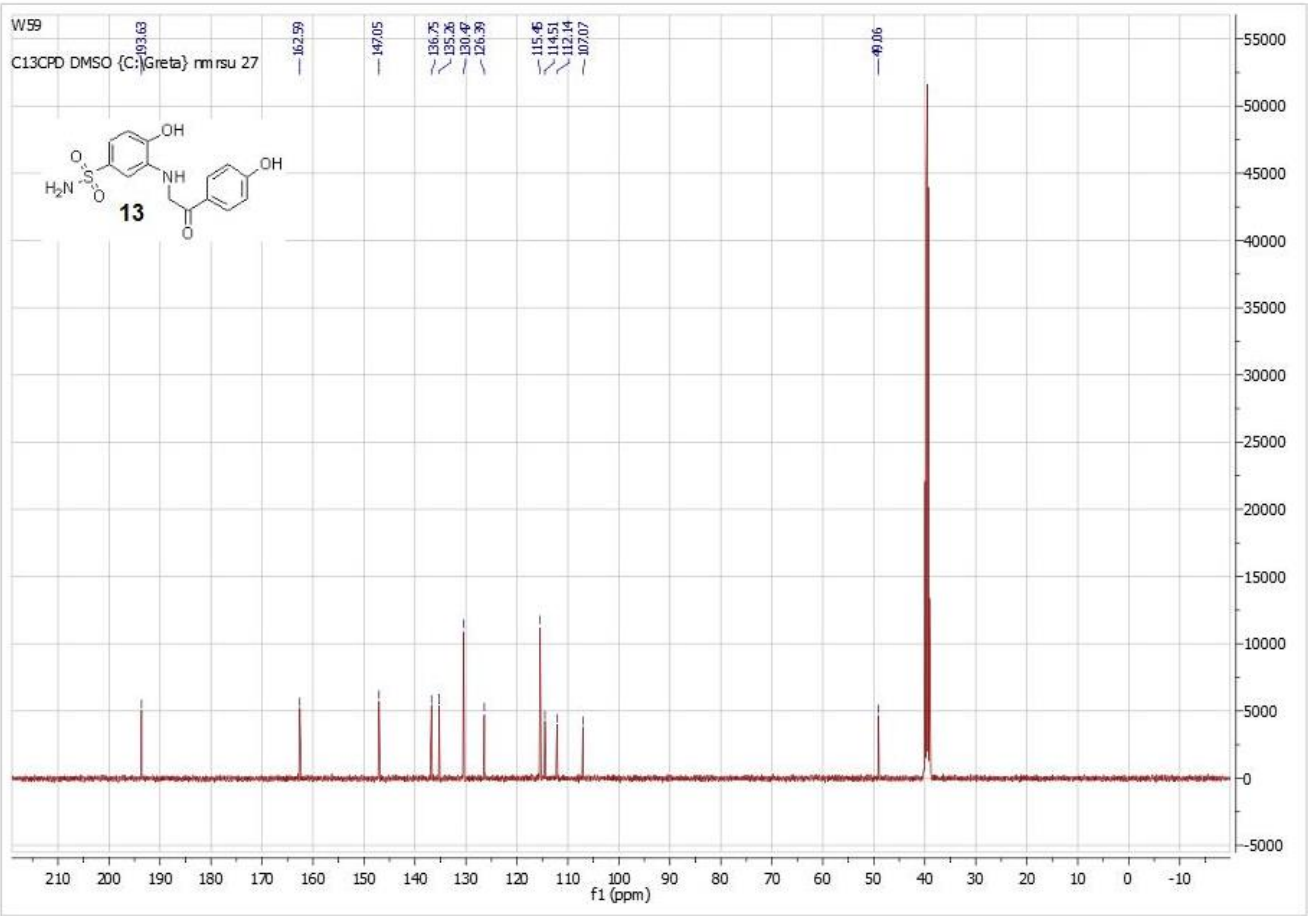

Figure S22. ^{13}C NMR of compound **13.**

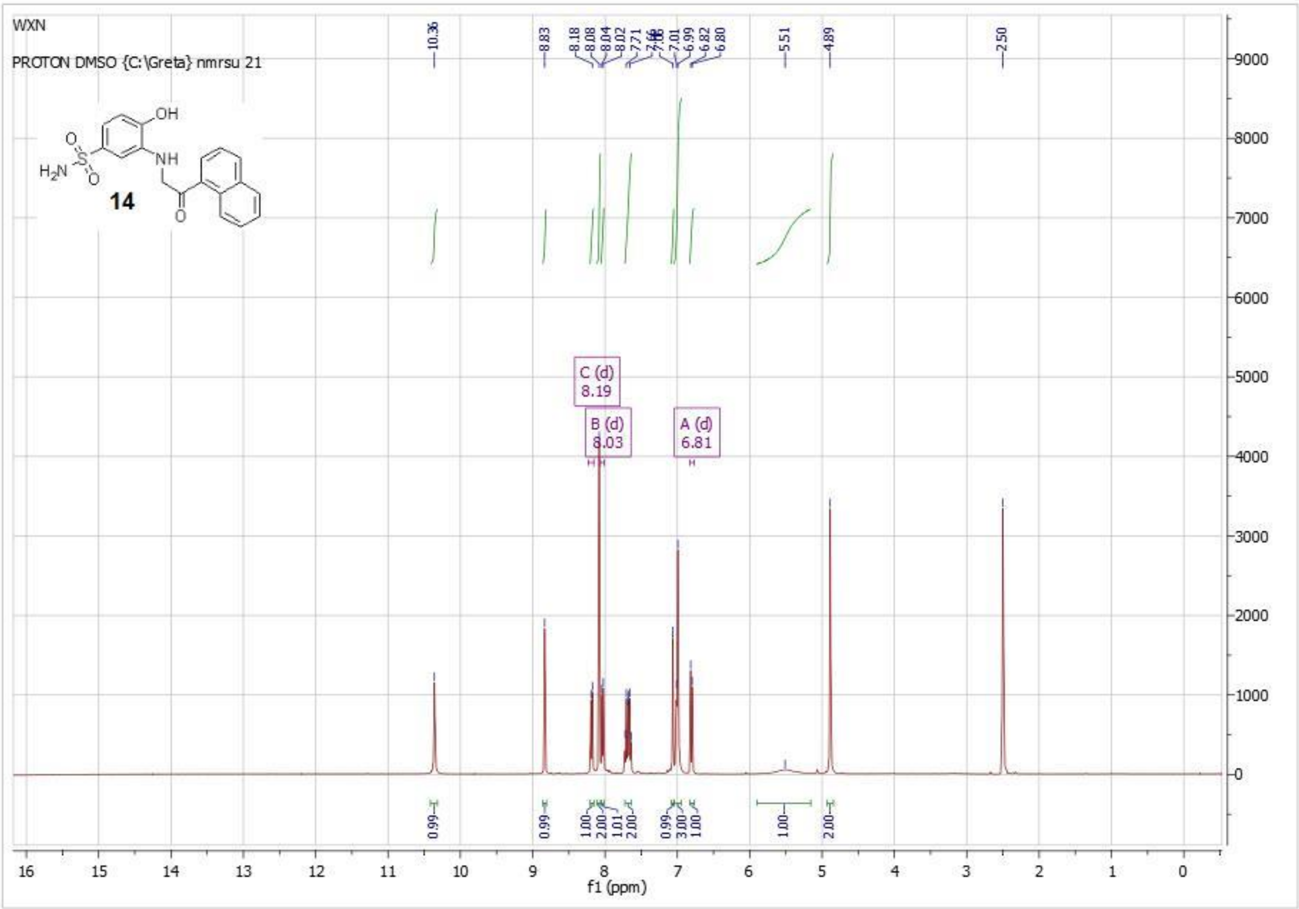

Figure S23. ^{1}H NMR of compound **14.**

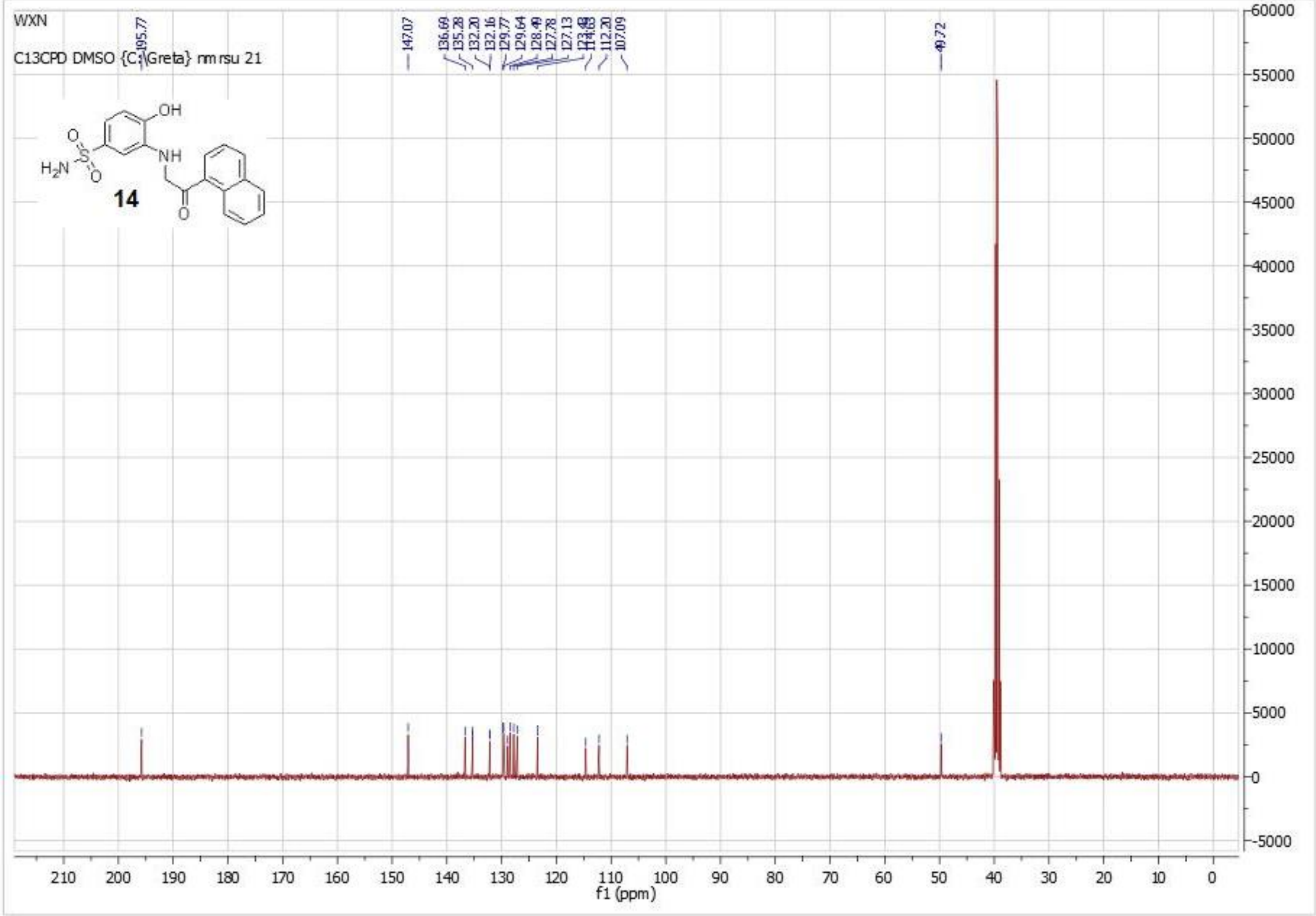

Figure S24. ^{13}C NMR of compound **14.**

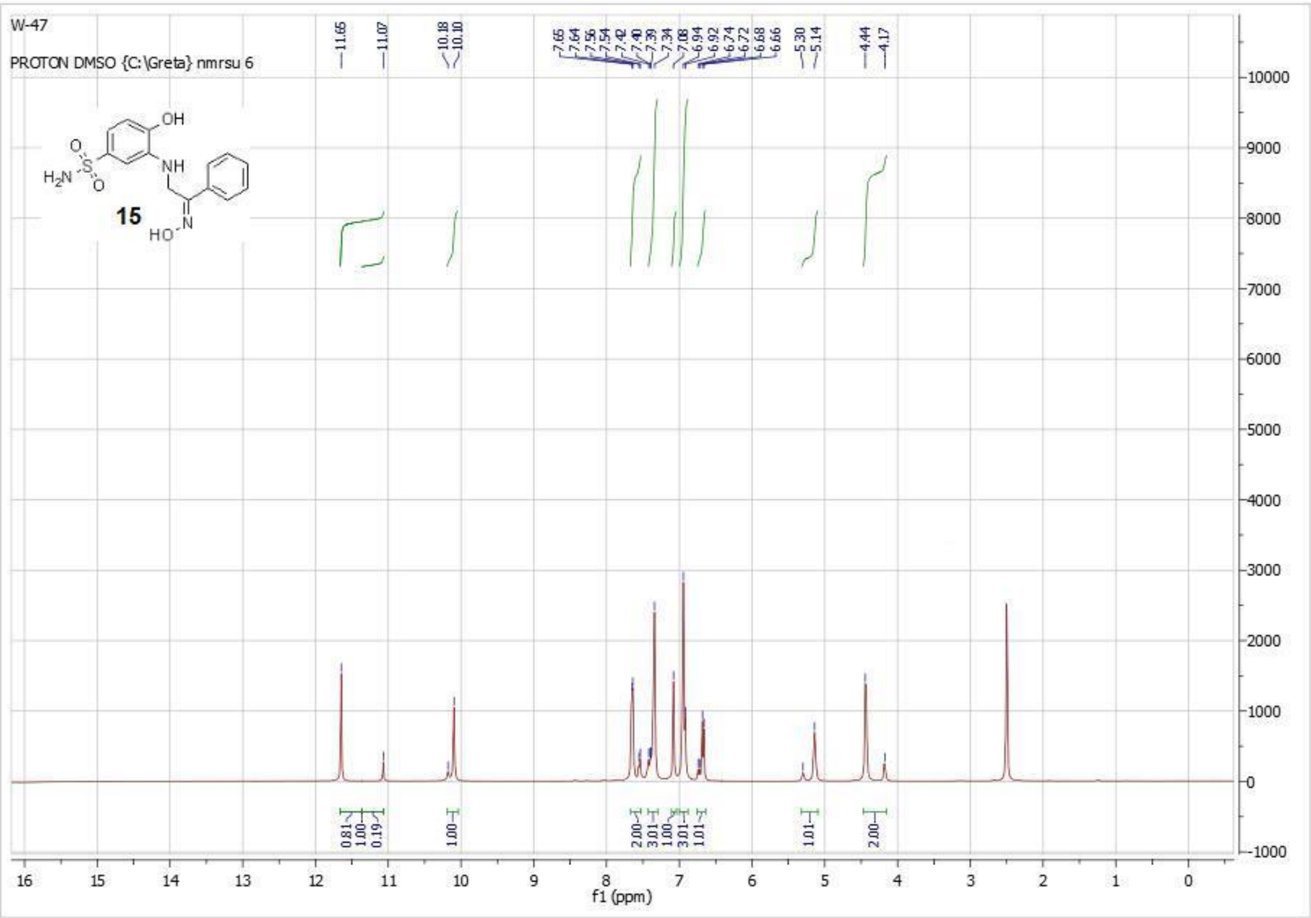

Figure S25. ^{1}H NMR of compound **15.**

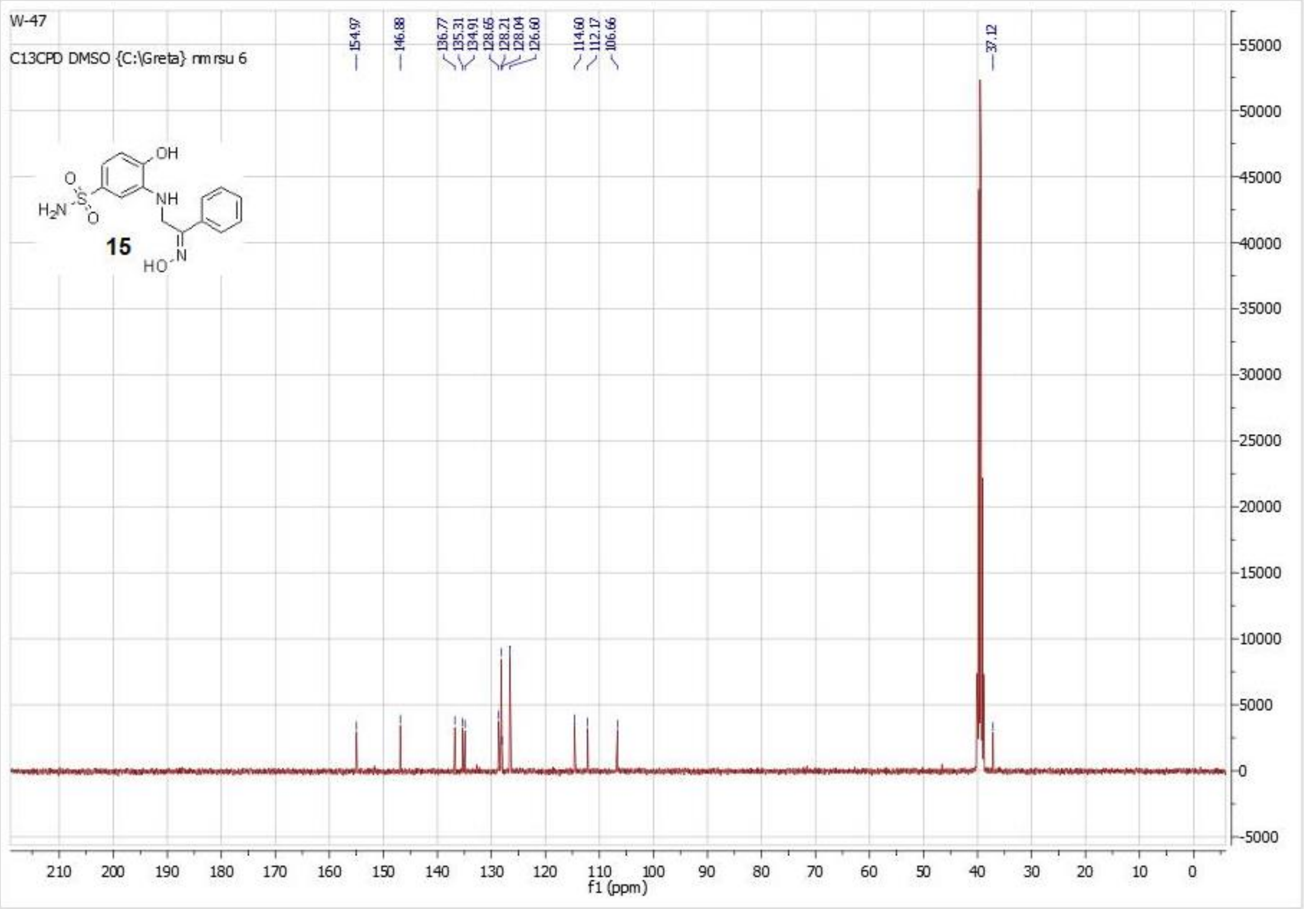

Figure S26. ^{13}C NMR of compound **15.**

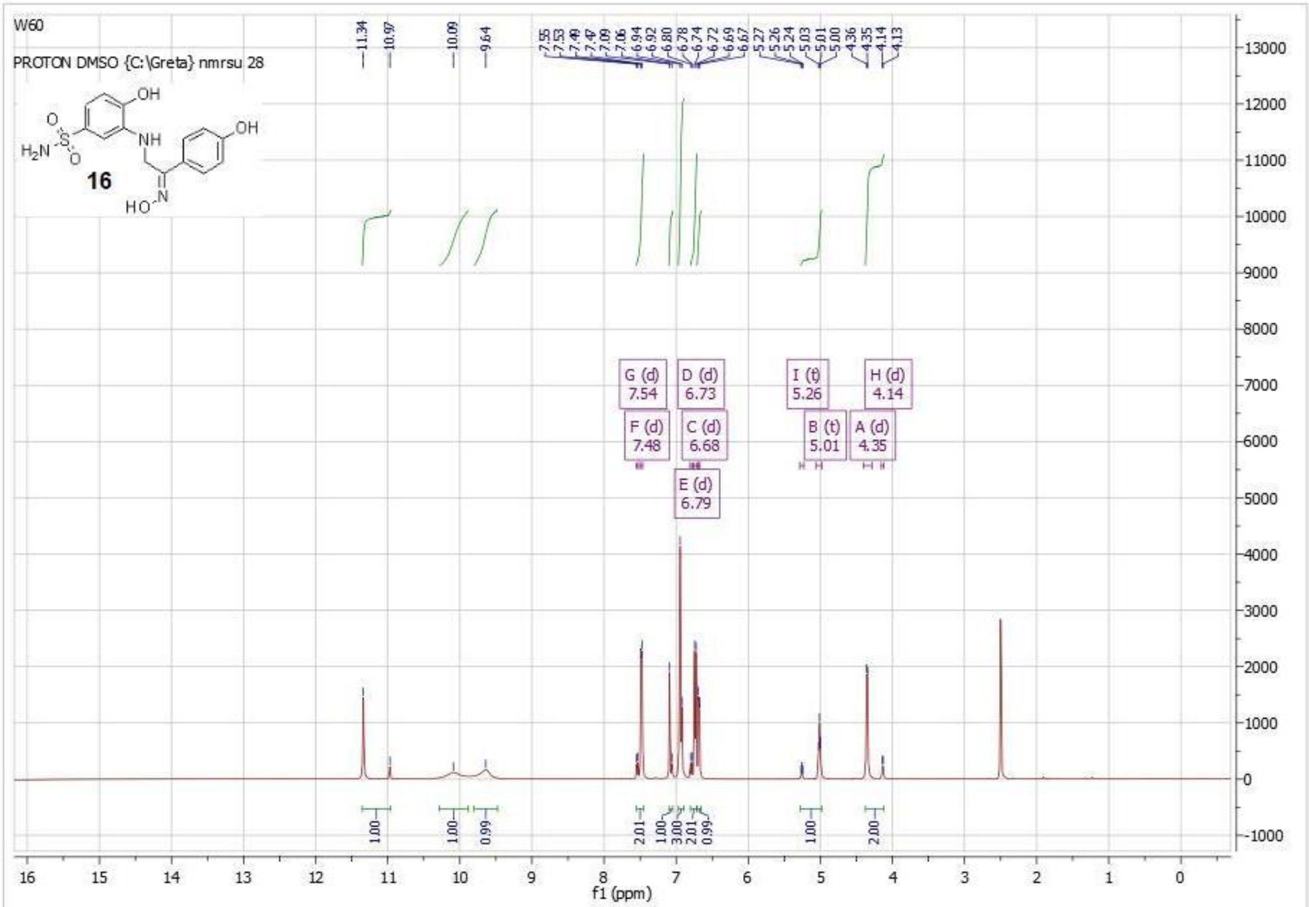

Figure S27. ^{1}H NMR of compound **16.**

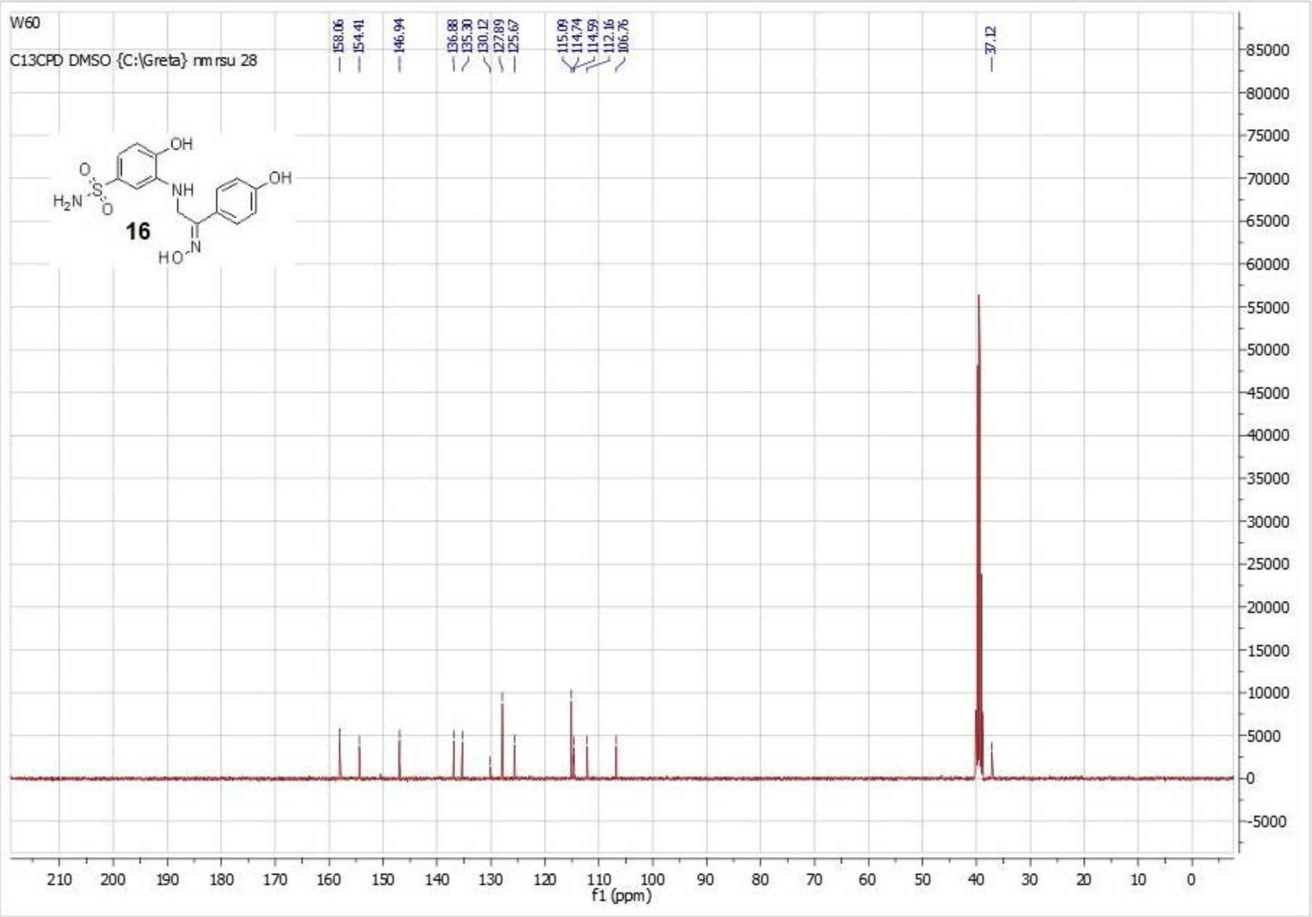

Figure S28. ^{13}C NMR of compound **16.**

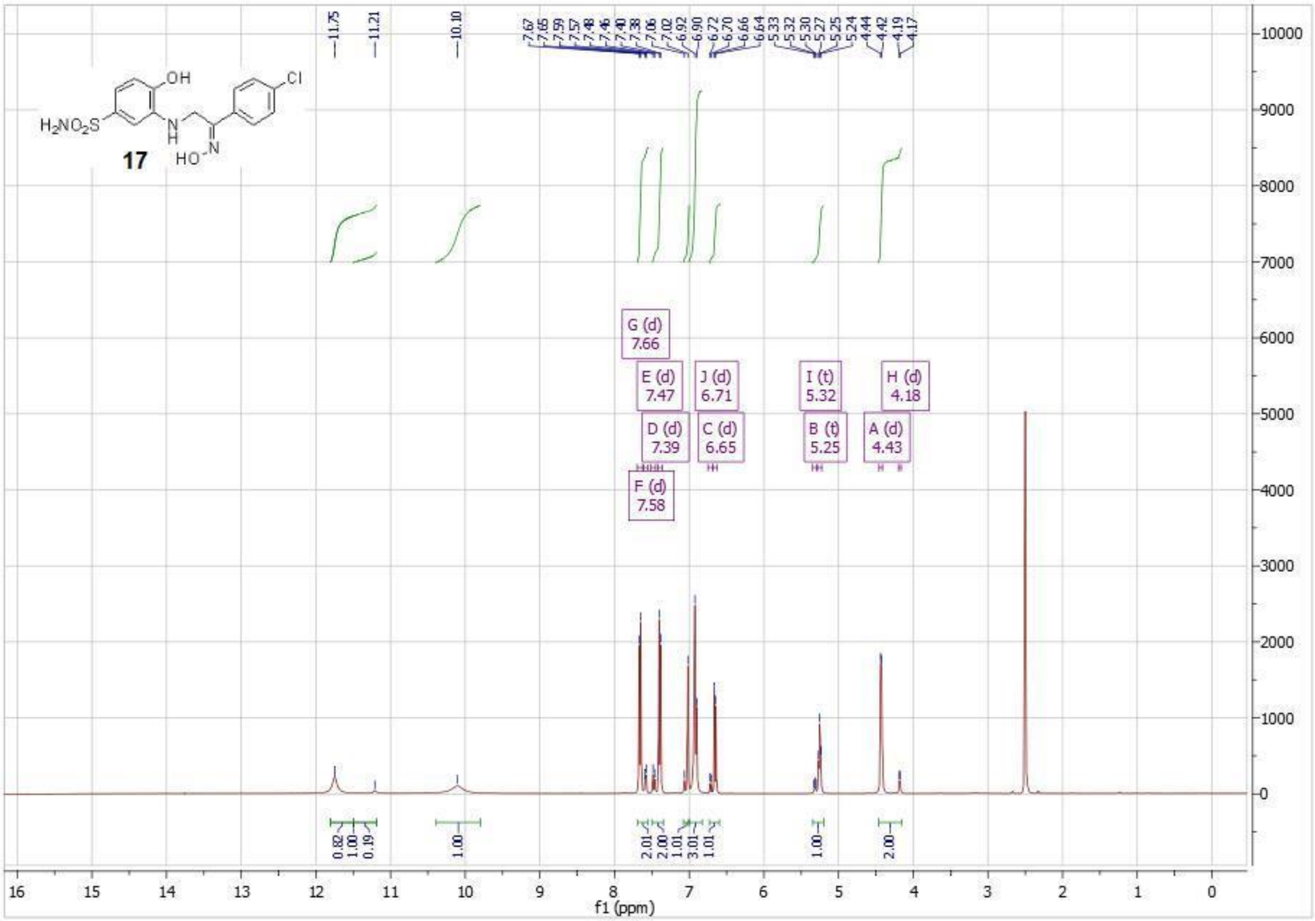

Figure S29. ^{1}H NMR of compound **17.**

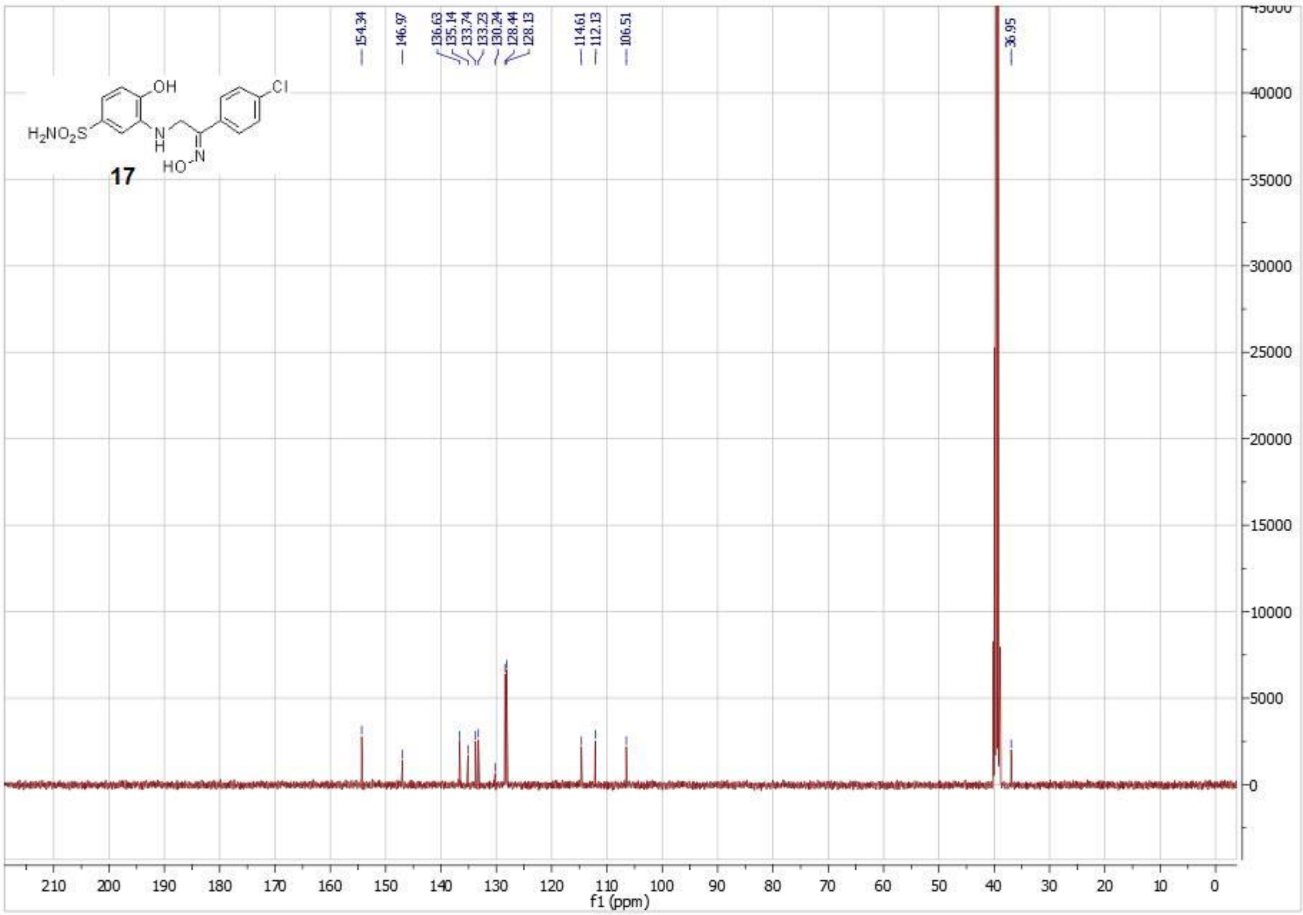

Figure S30. ^{13}C NMR of compound **17.**

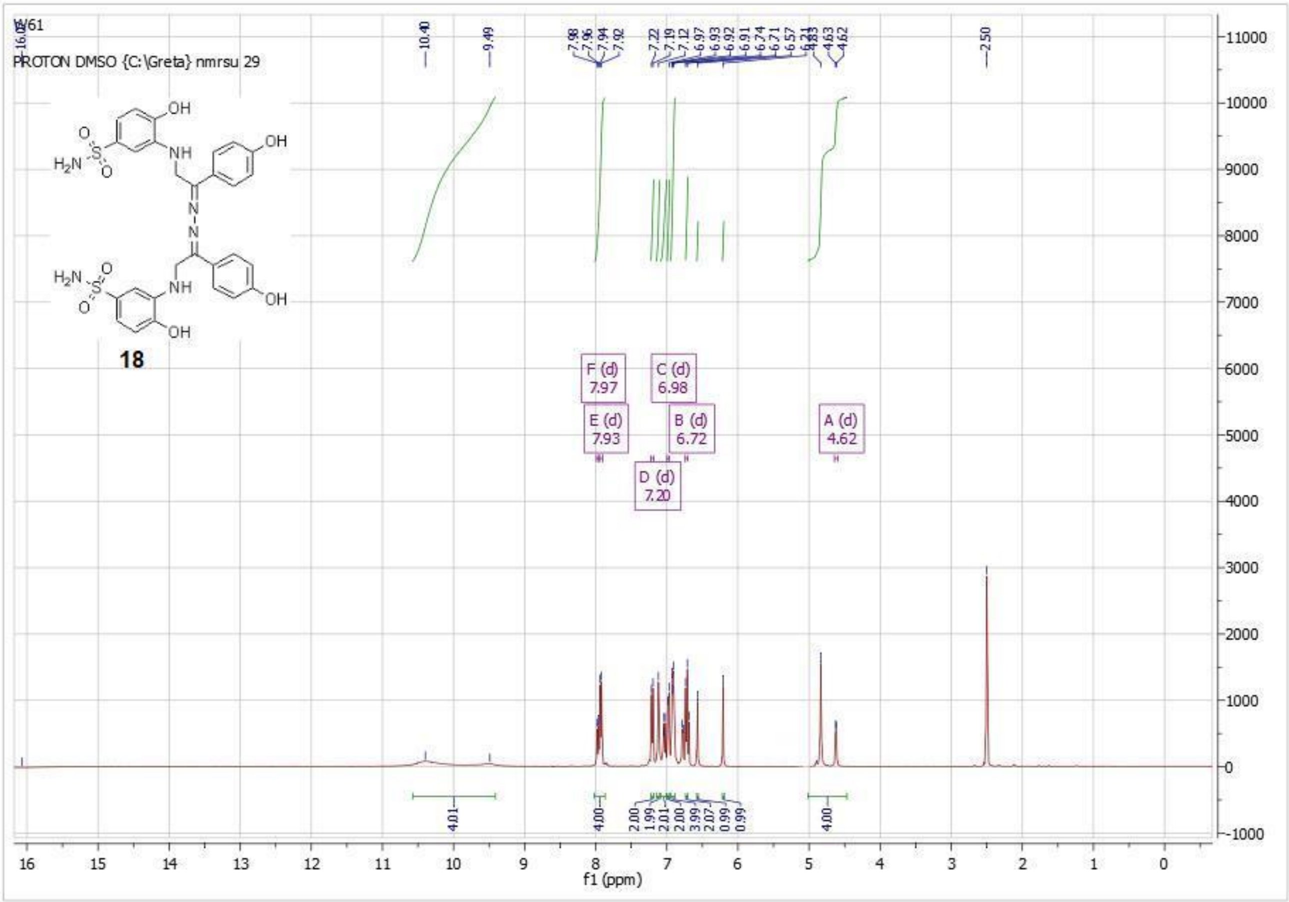

Figure S31. ^{1}H NMR of compound **18.**

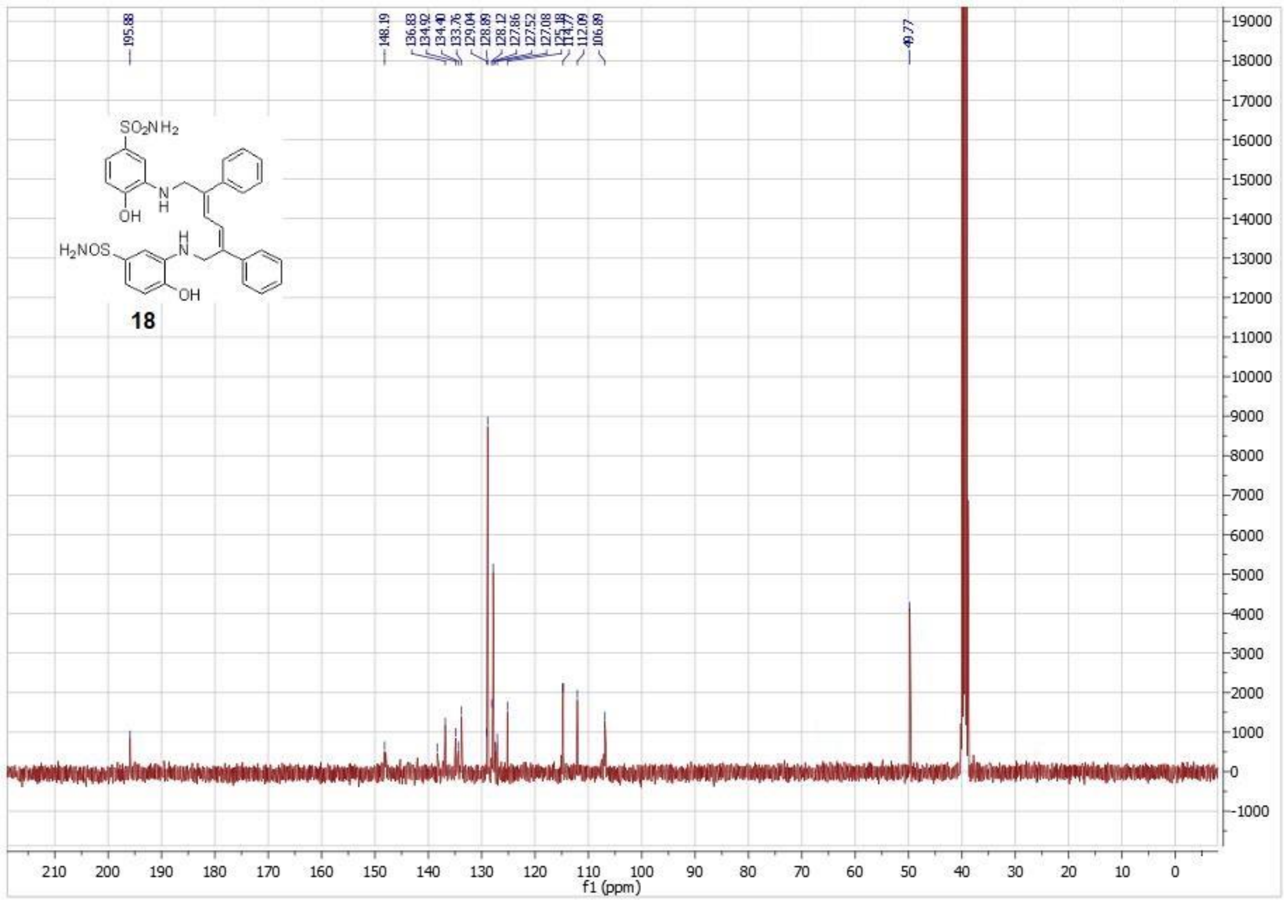

Figure S32. ^{13}C NMR of compound **18.**

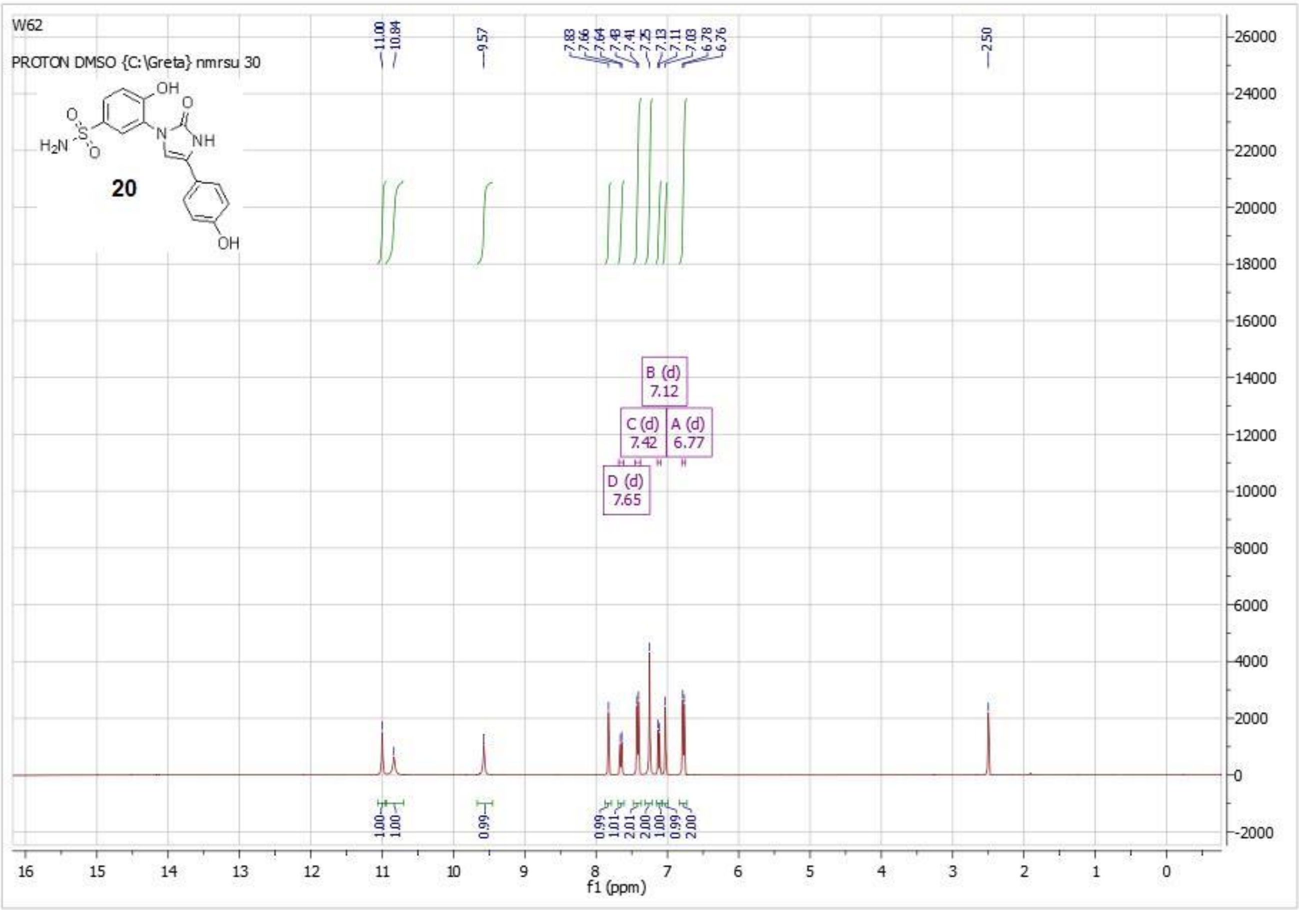

Figure S33. ^{1}H NMR of compound **20.**

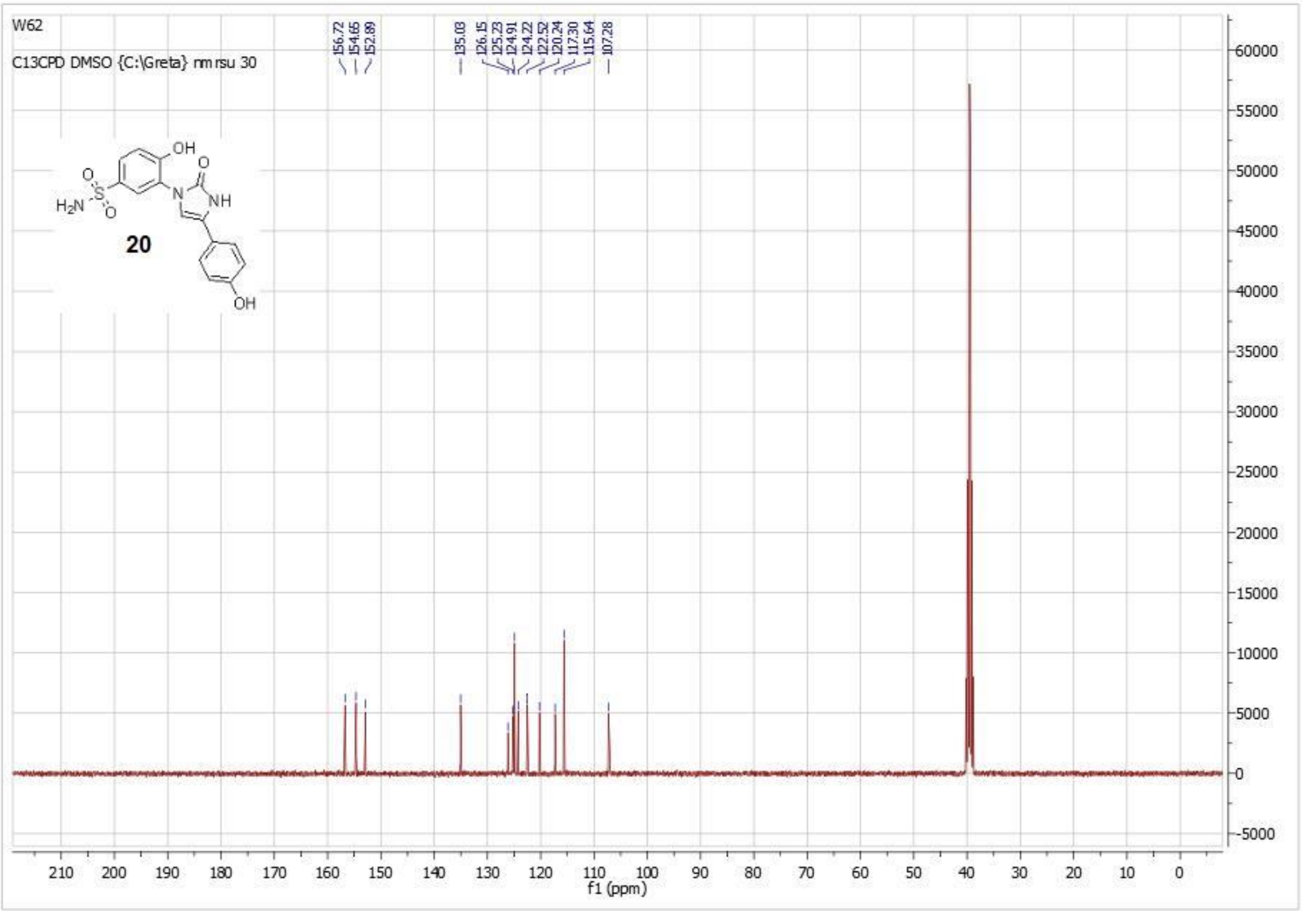

Figure S34. ^{13}C NMR of compound **20.**

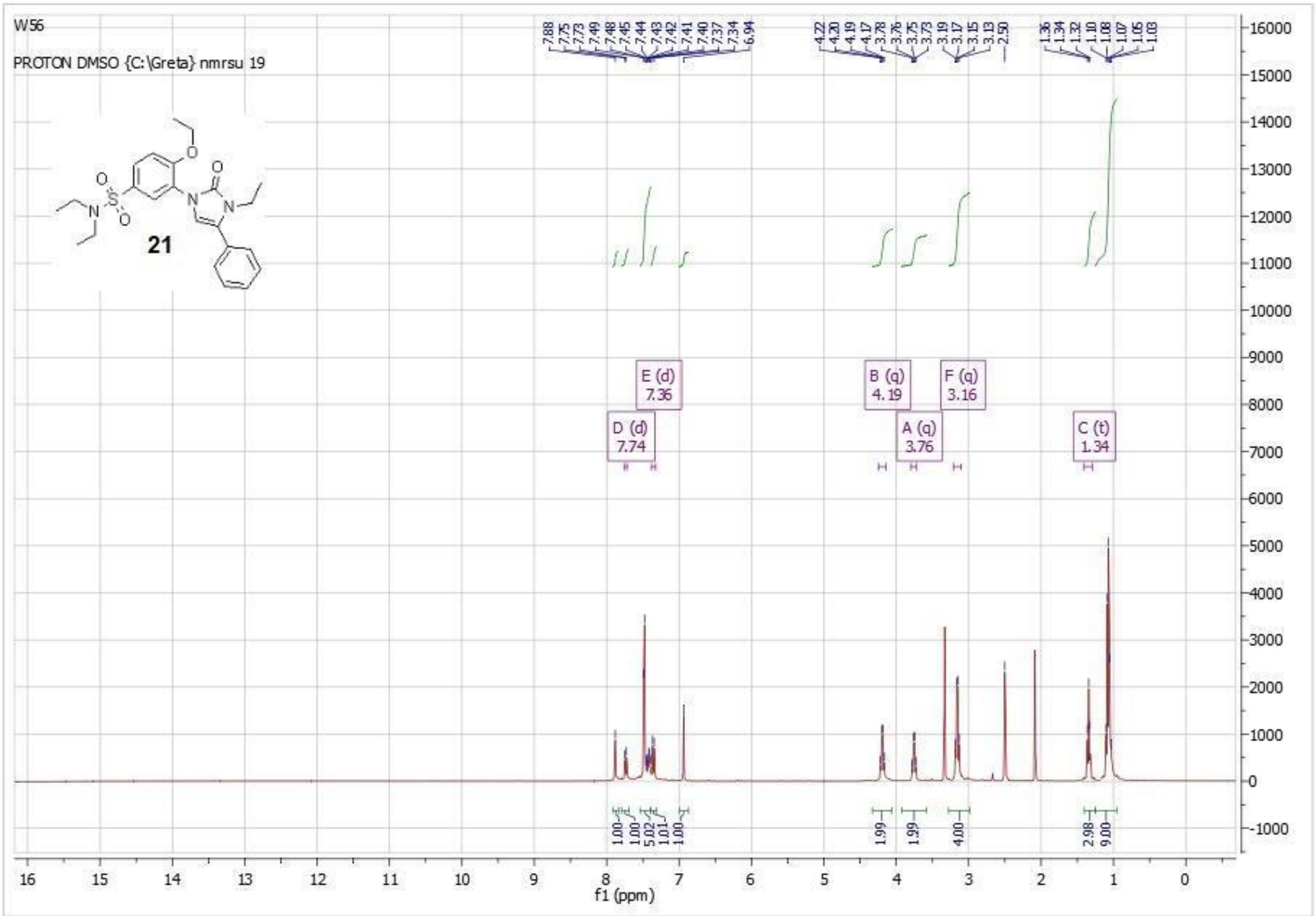

Figure S35. ¹H NMR of compound **21.**

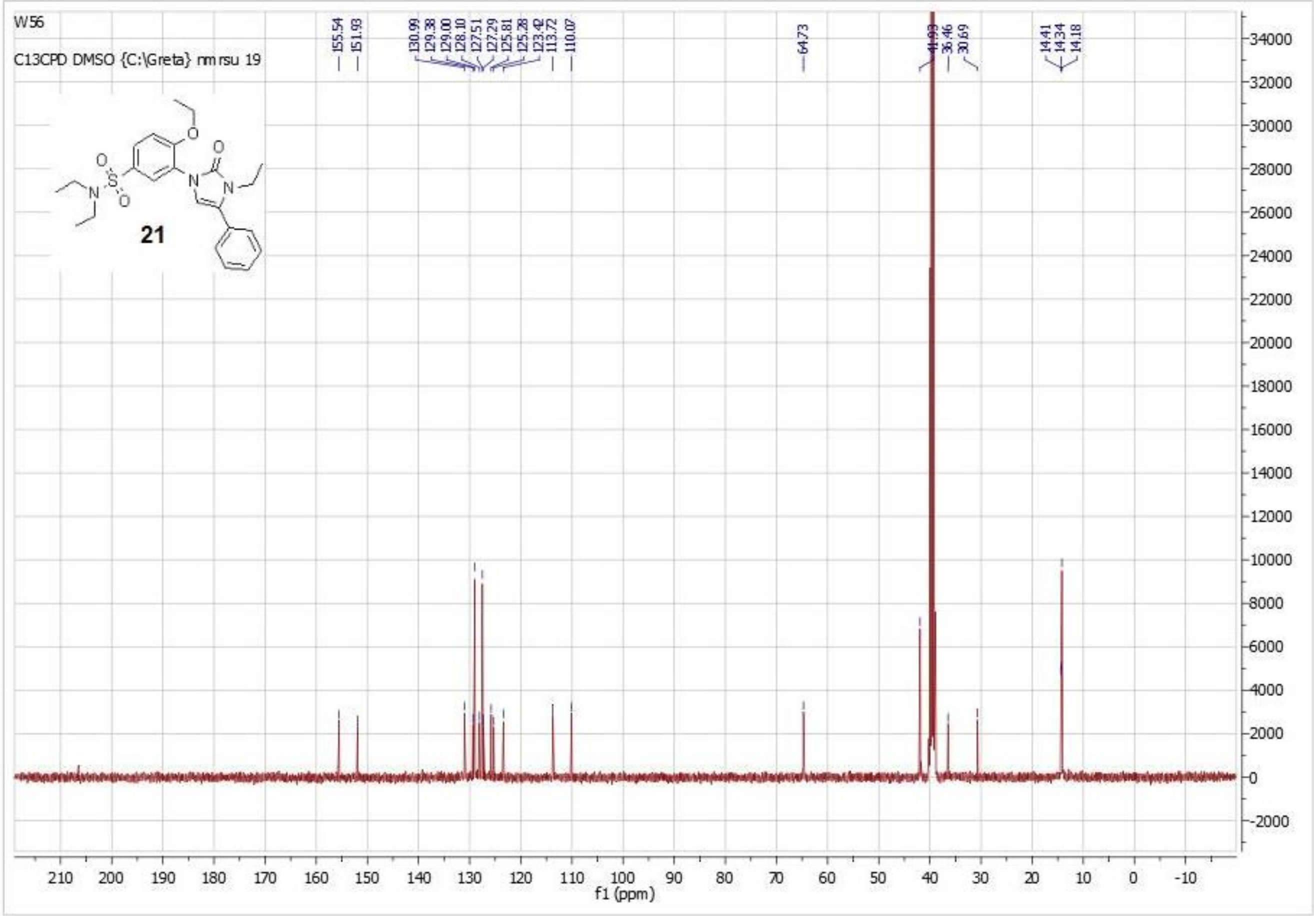

Figure S36. ¹³C NMR of compound **21.**

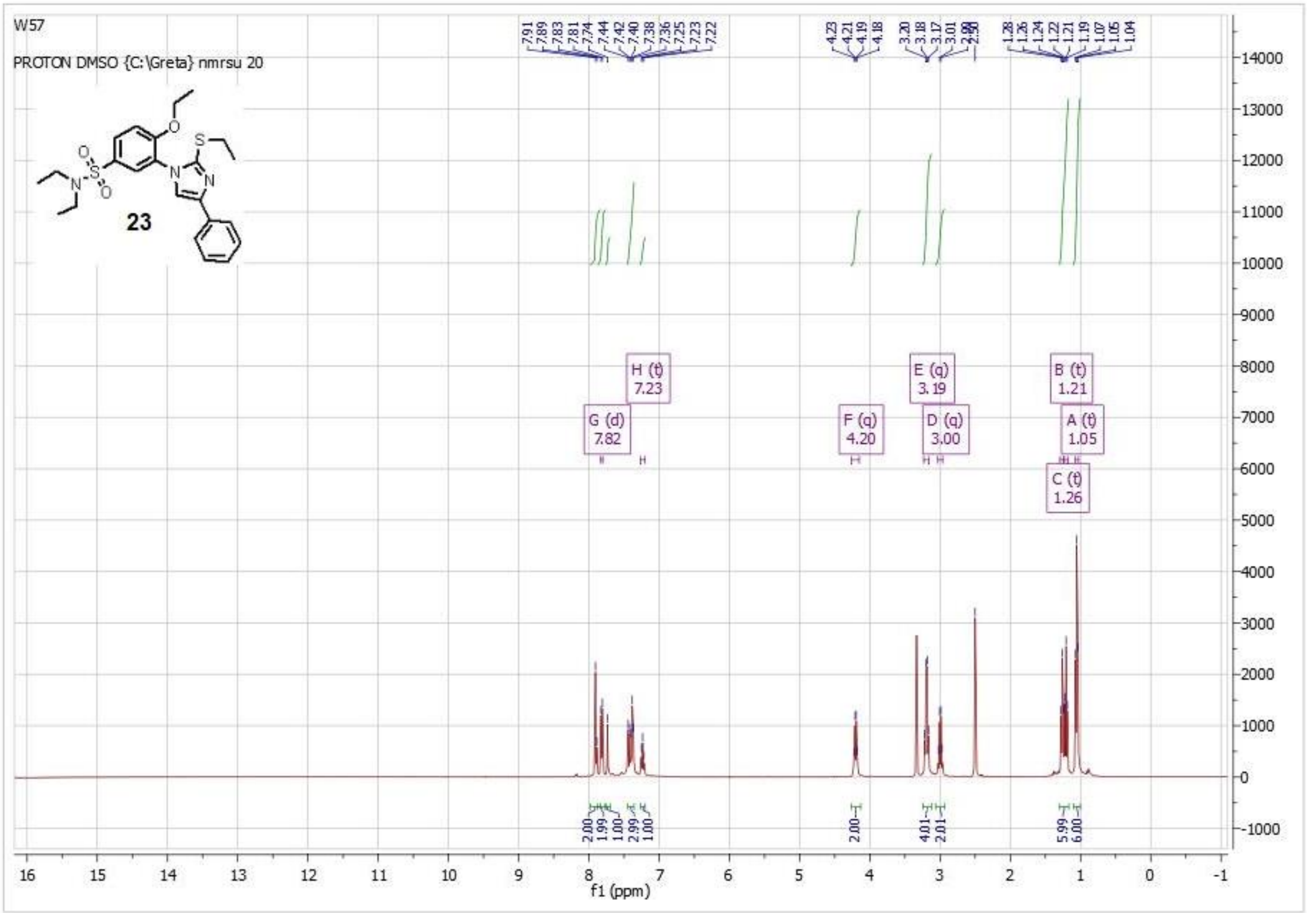

Figure S37 ^{1}H NMR of compound **23.**

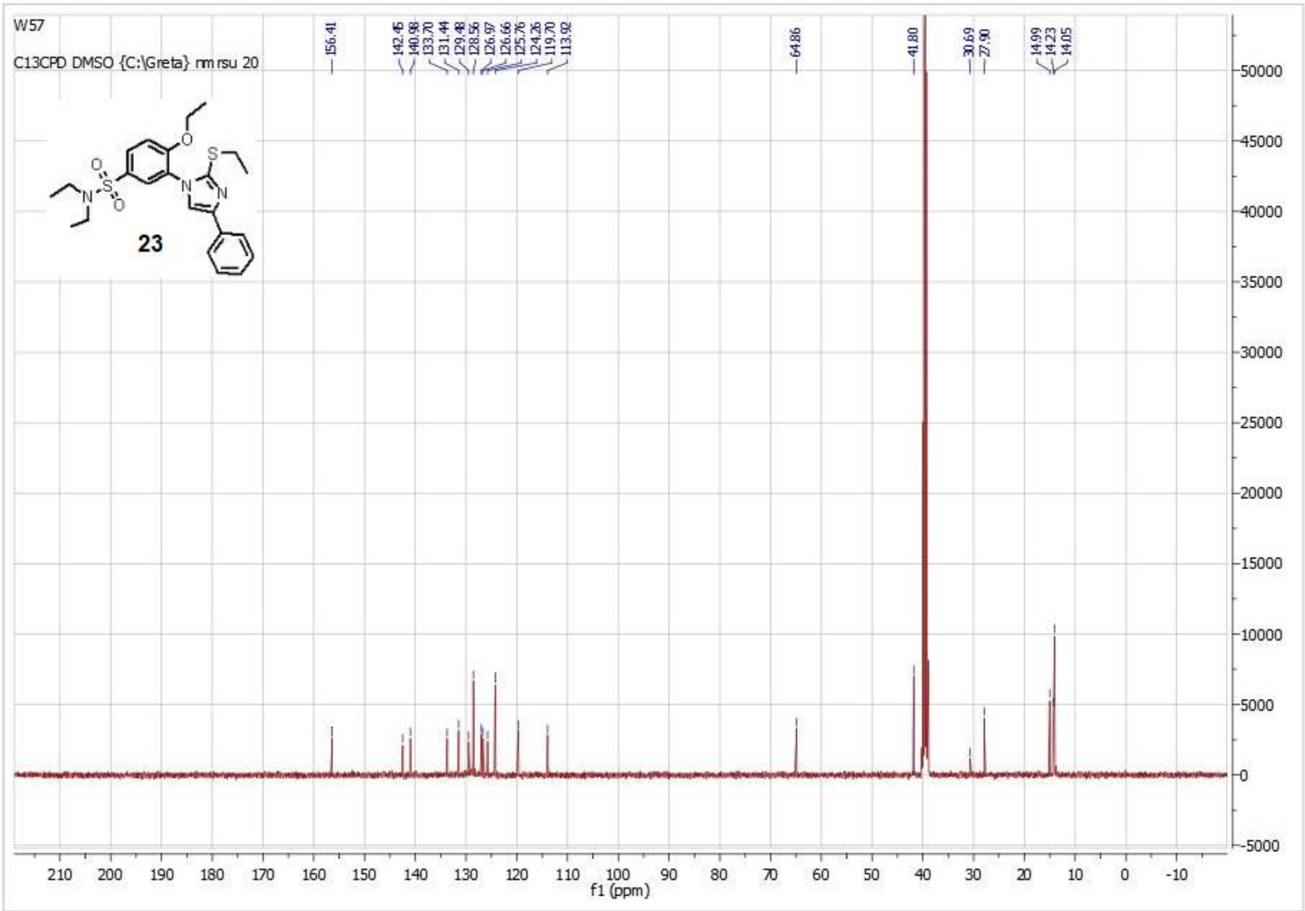

Figure S38. ^{13}C NMR of compound **23.**

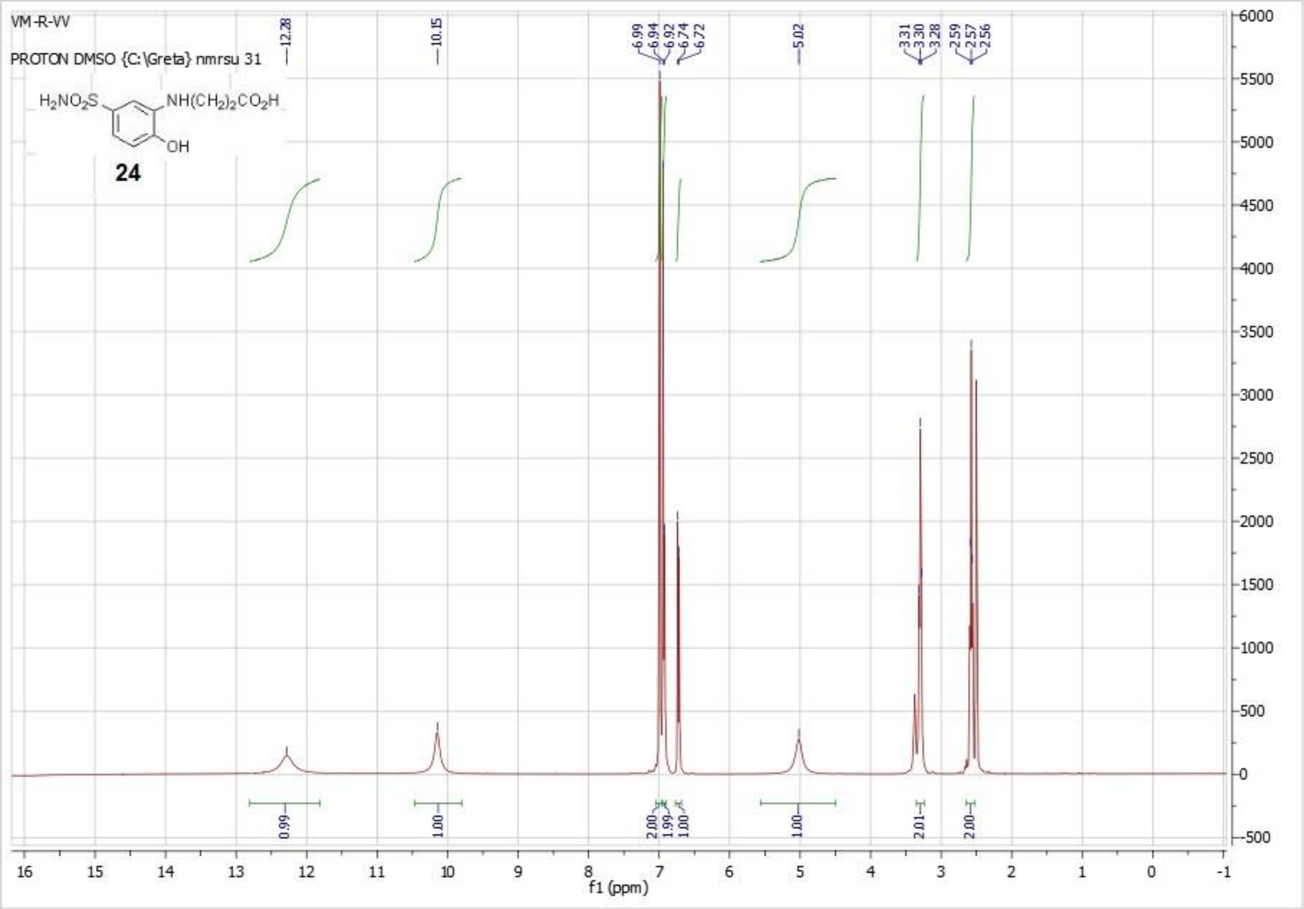

Figure S39. ^{1}H NMR of compound **24.**

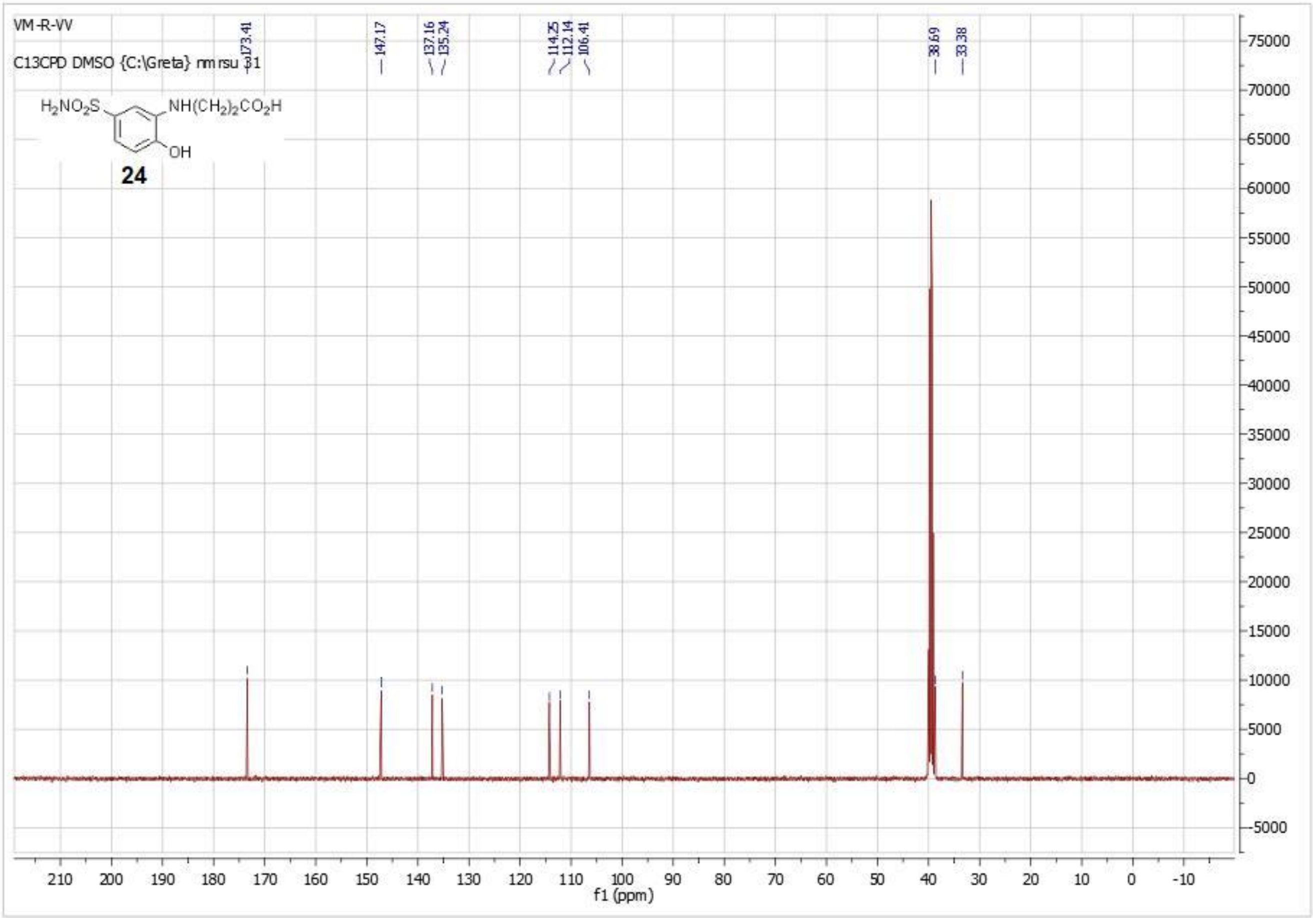

Figure S40. ^{13}C NMR of compound **24.**

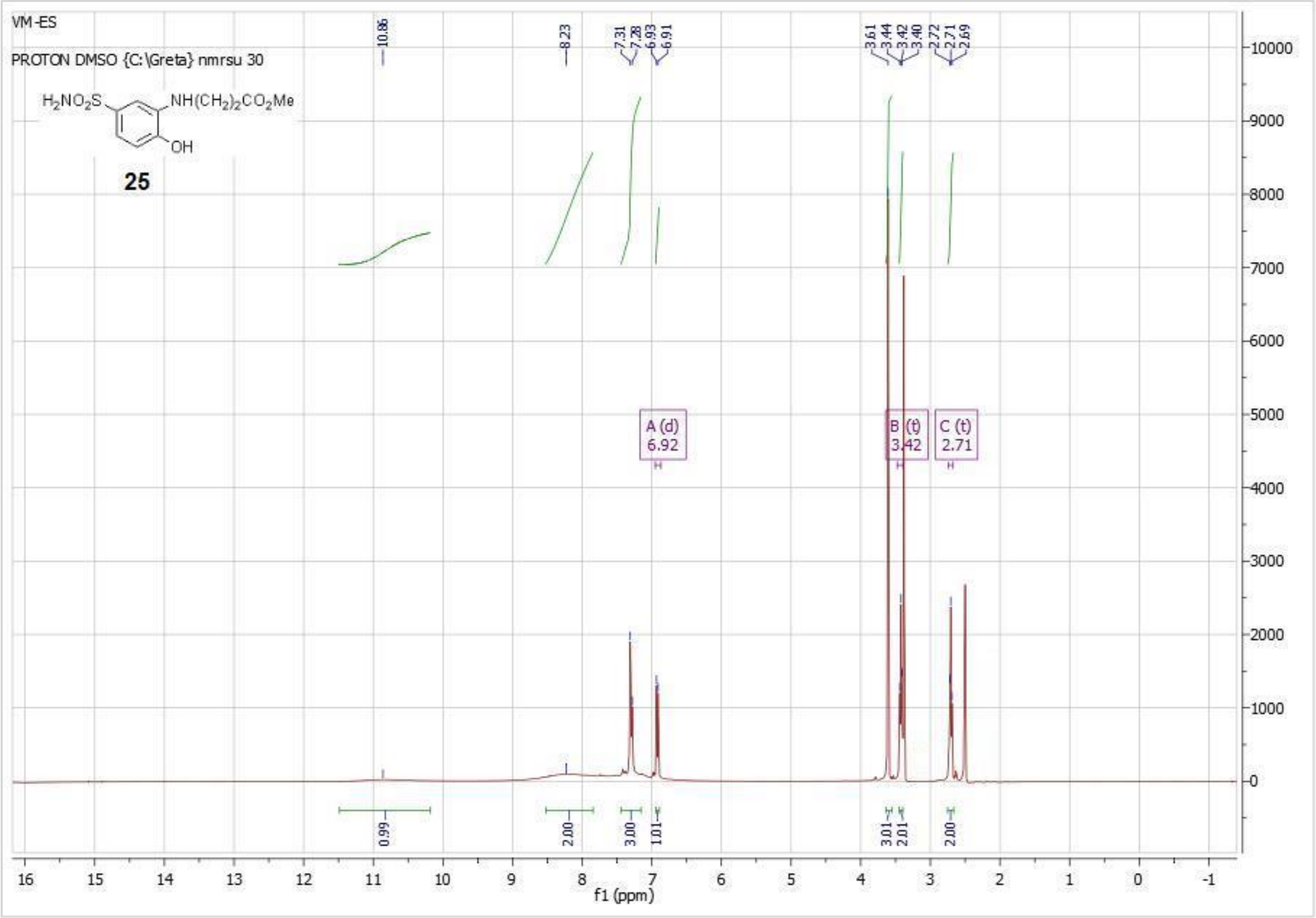

Figure S41. ^{1}H NMR of compound **25.**

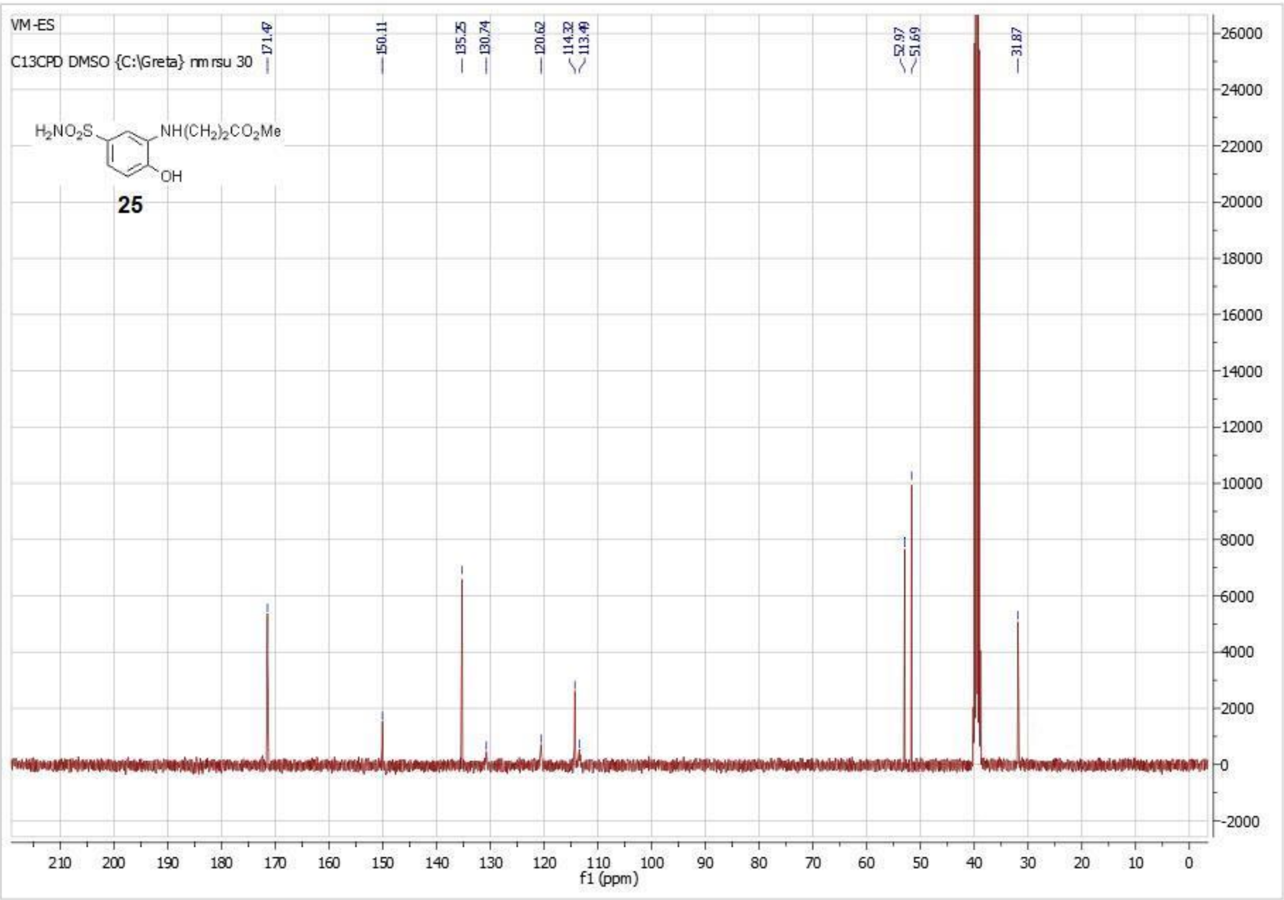

Figure S42. ^{13}C NMR of compound **25.**

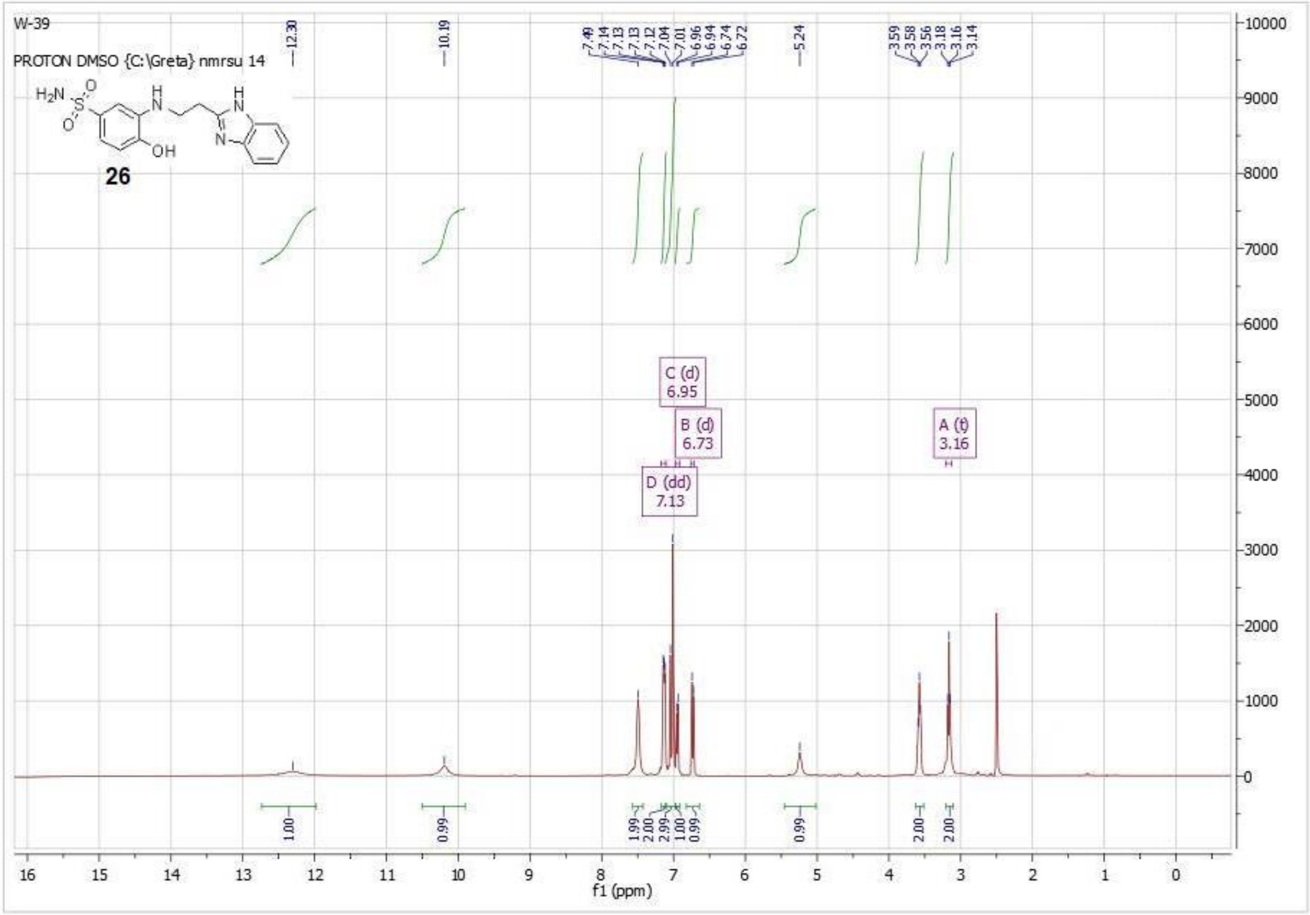

Figure S43. ^{1}H NMR of compound **26.**

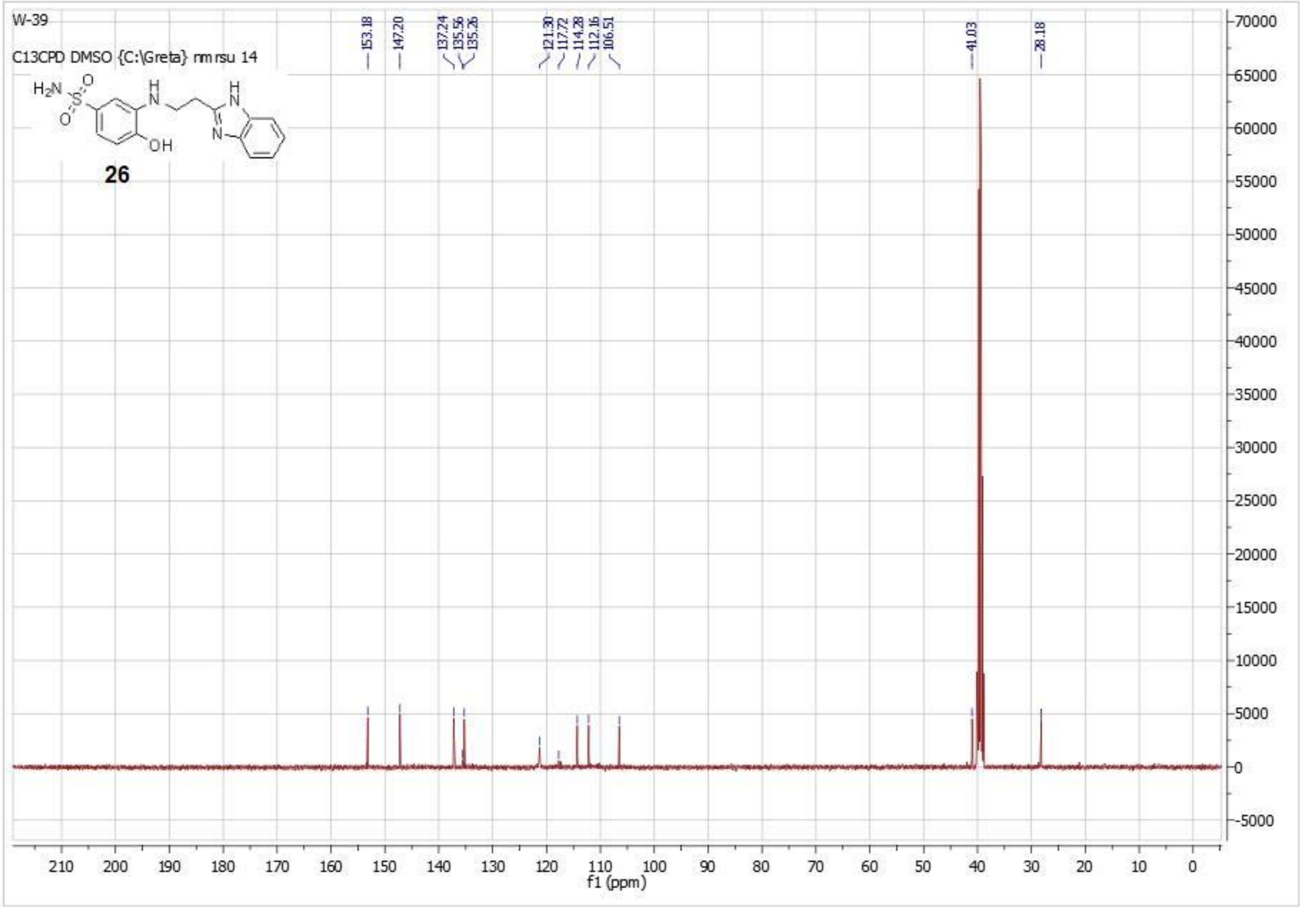

Figure S44. ^{13}C NMR of compound **26.**

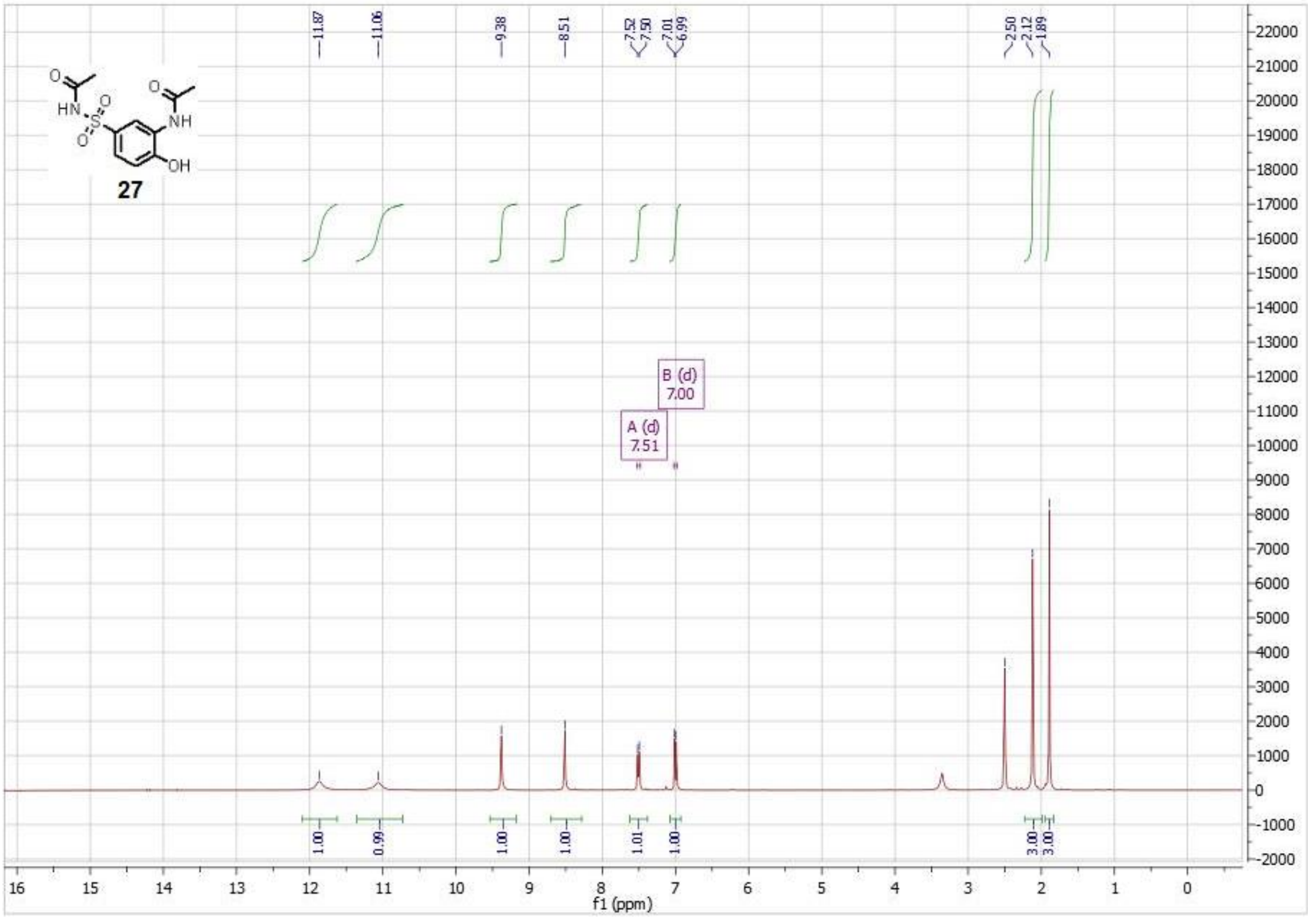

Figure S45. ^{1}H NMR of compound **27.**

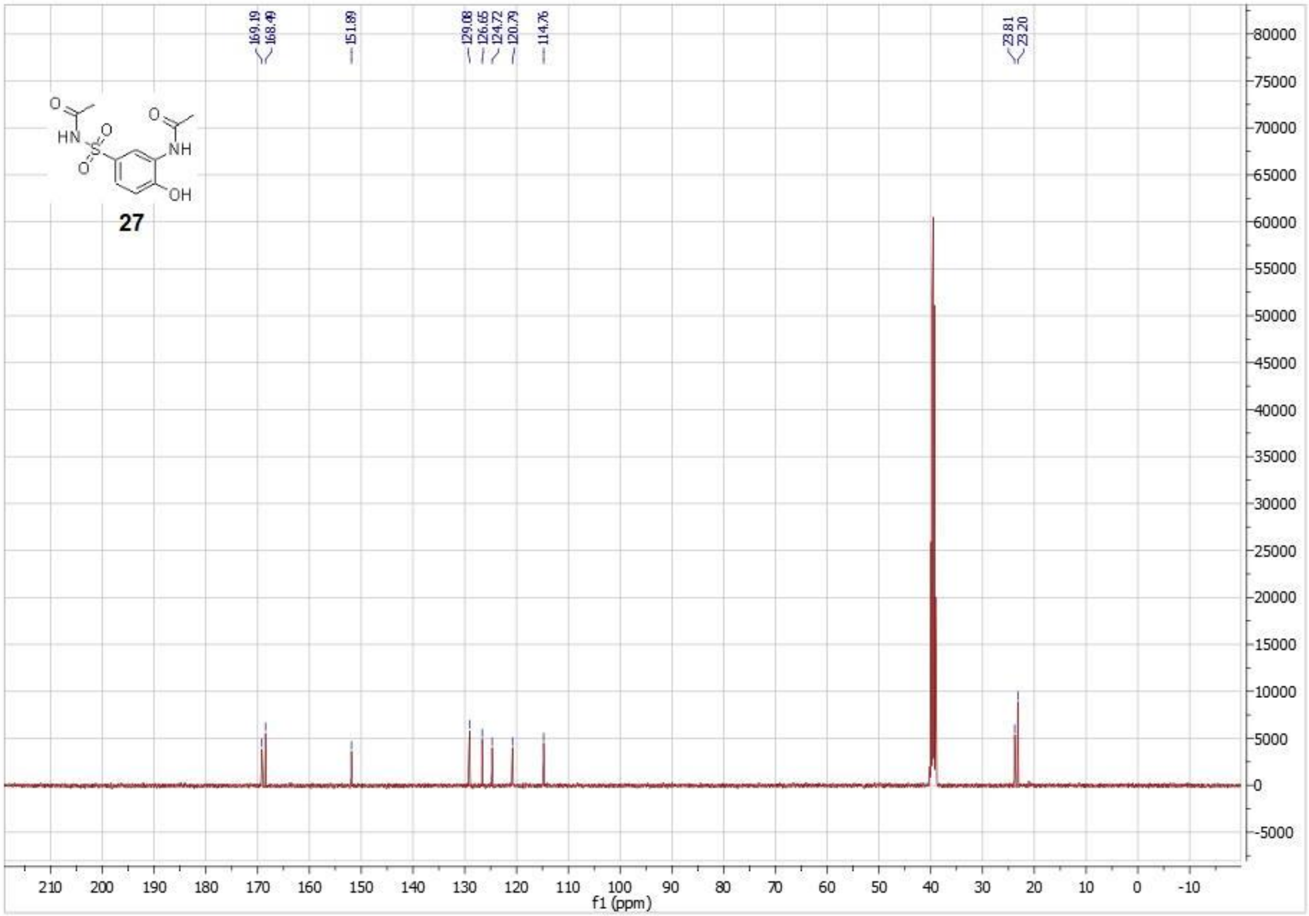

Figure S46. ^{13}C NMR of compound **27.**

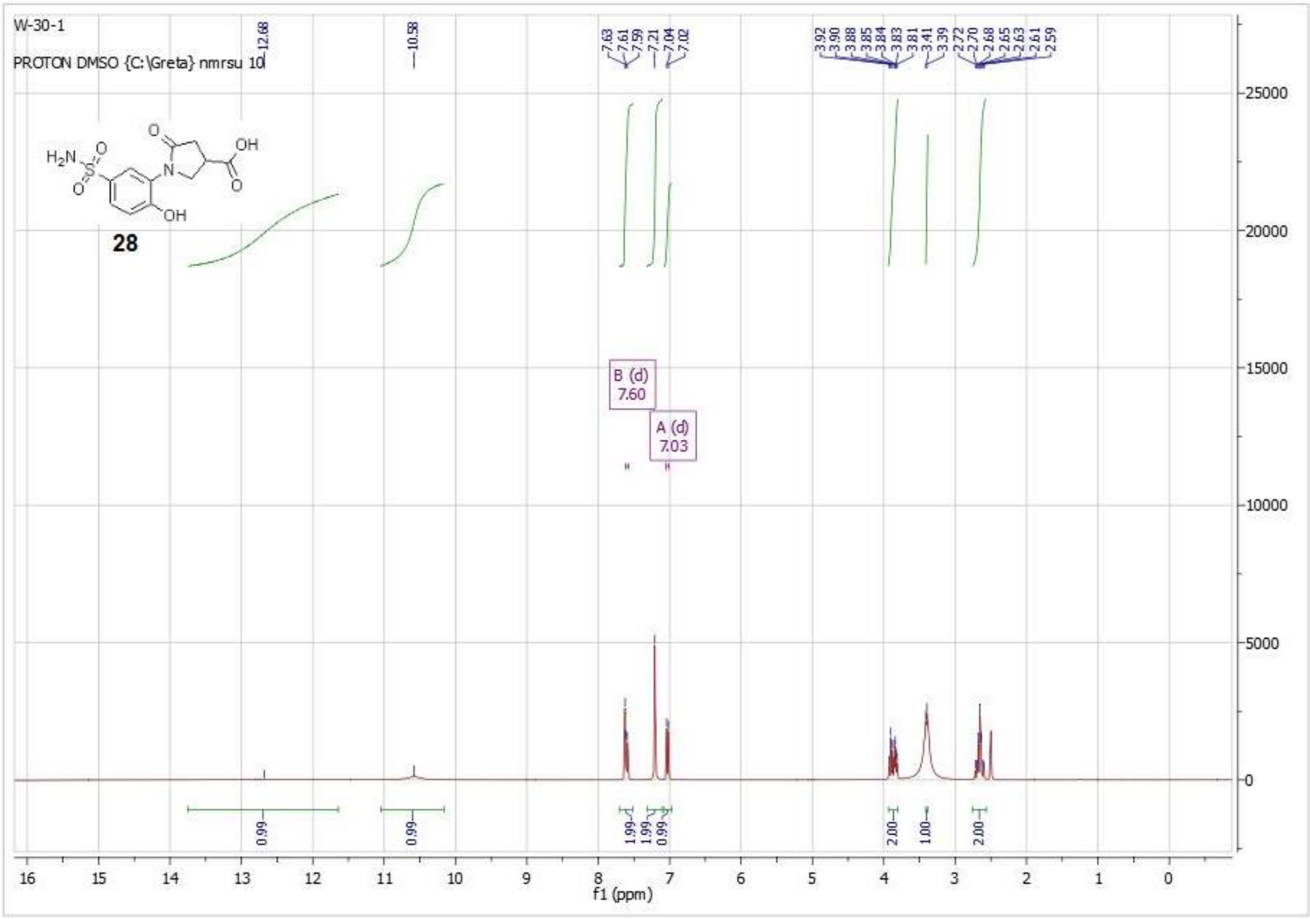

Figure S47. ^{1}H NMR of compound **28.**

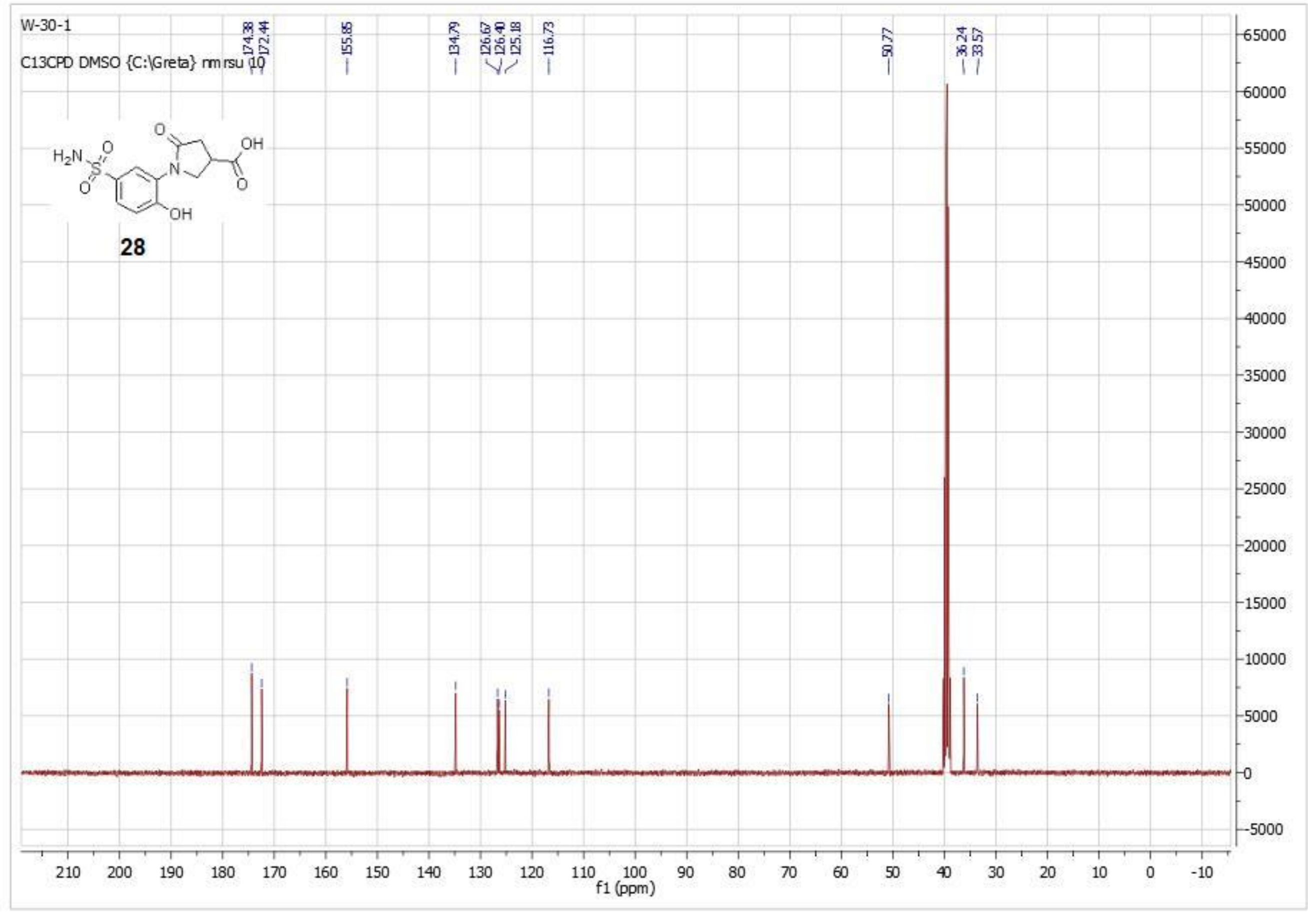

Figure S48. ^{13}C NMR of compound **28.**

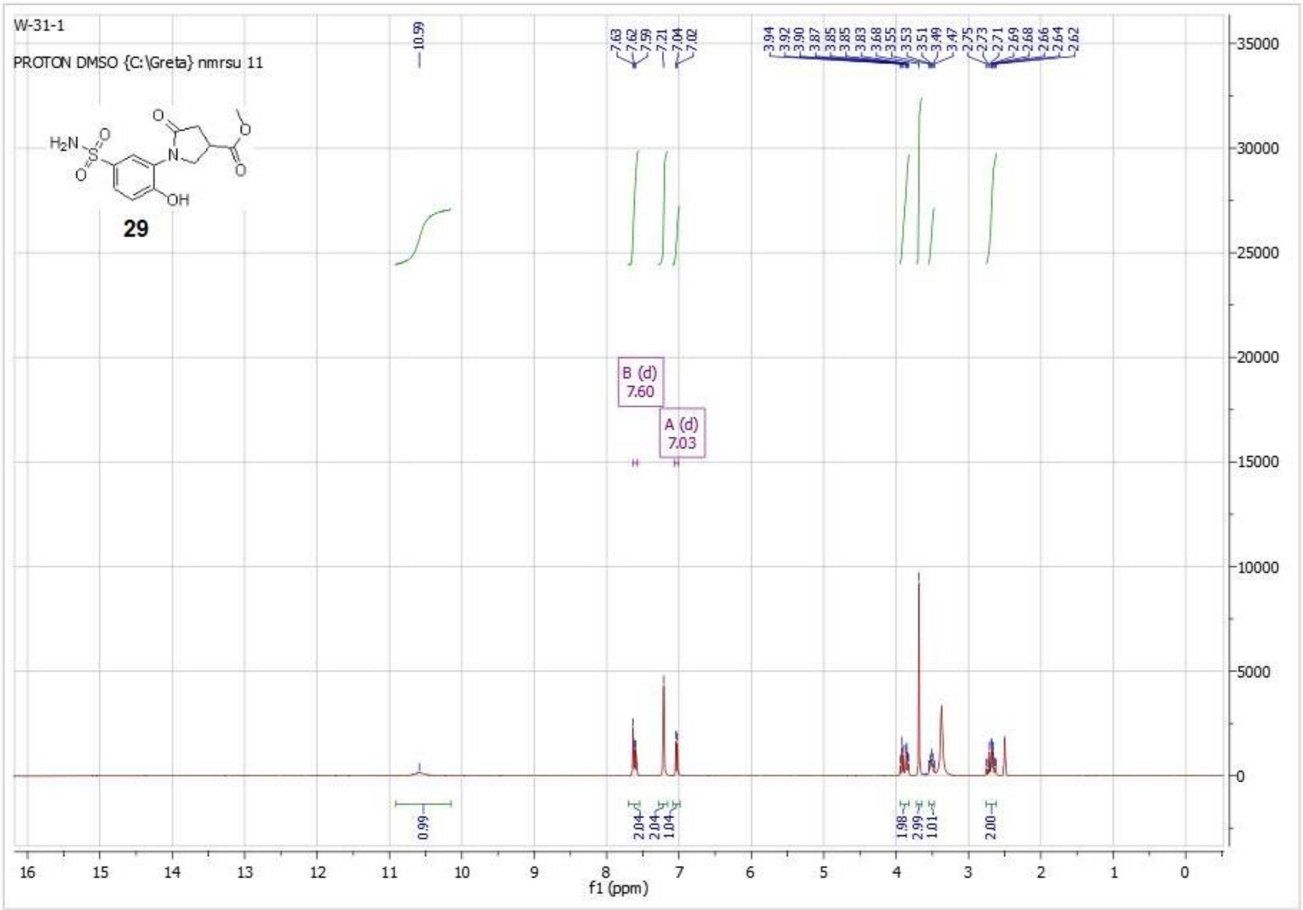

Figure S49. ^{1}H NMR of compound **29.**

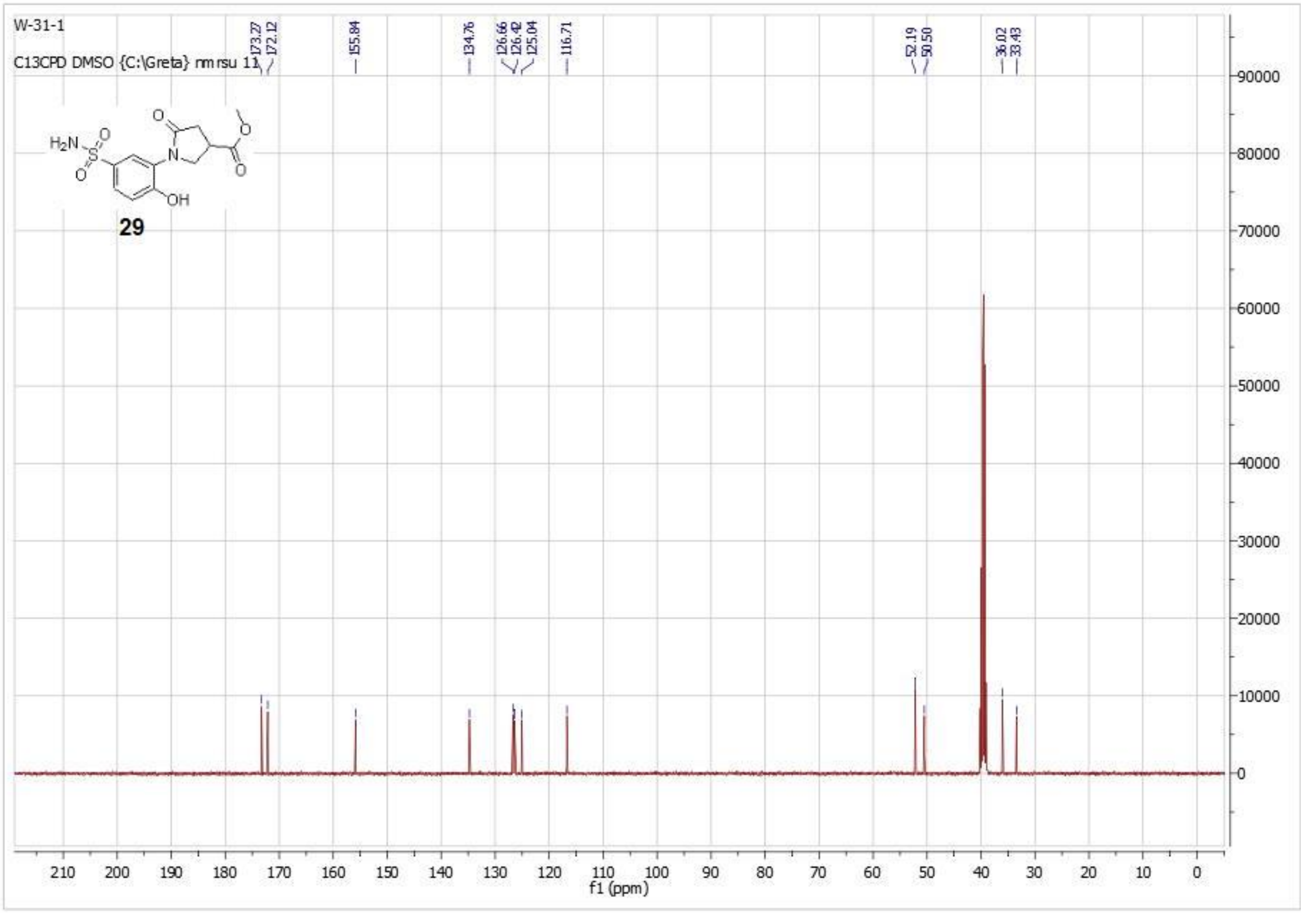

Figure S50. ^{13}C NMR of compound **29.**

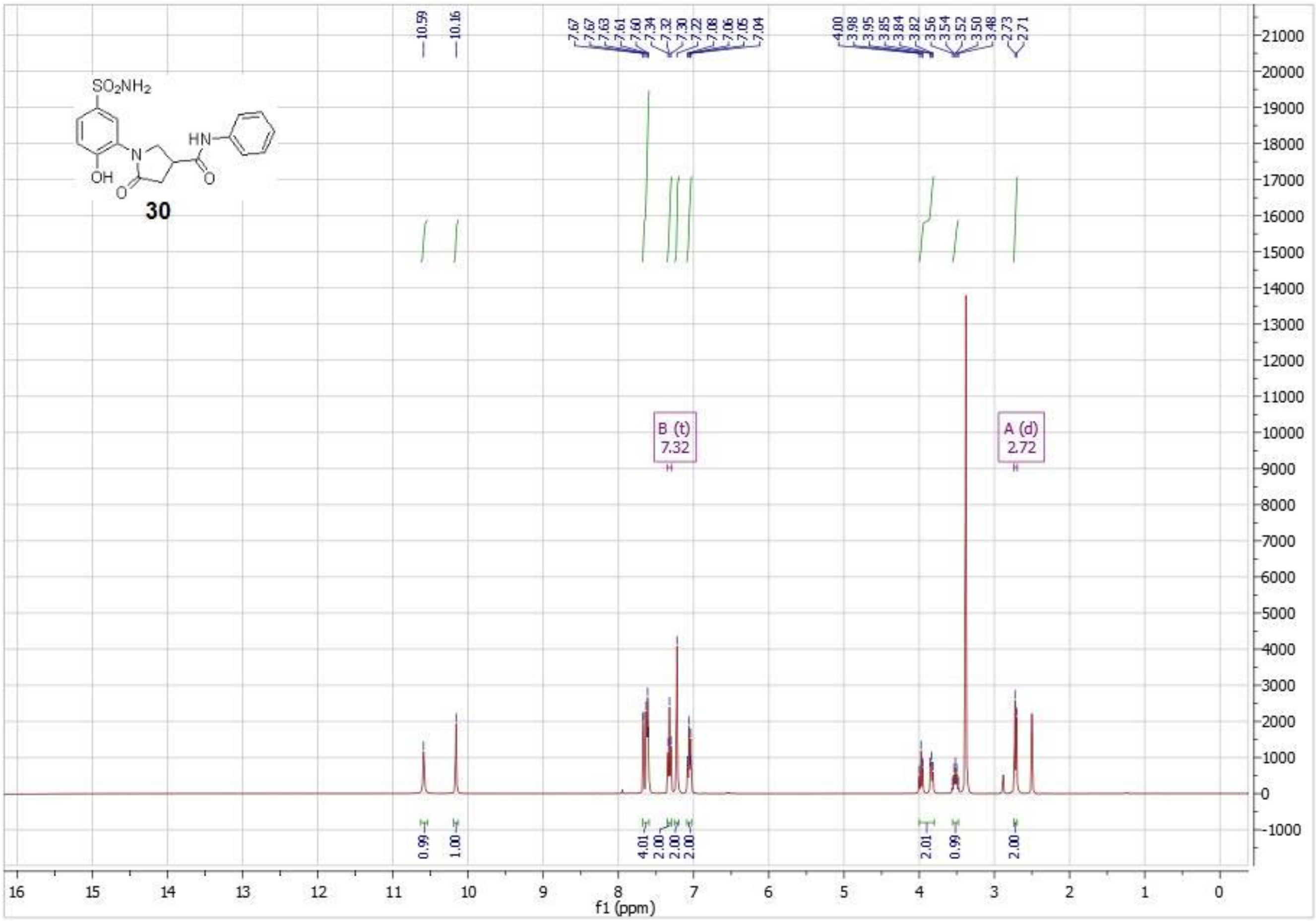

Figure S51. ^{1}H NMR of compound **30.**

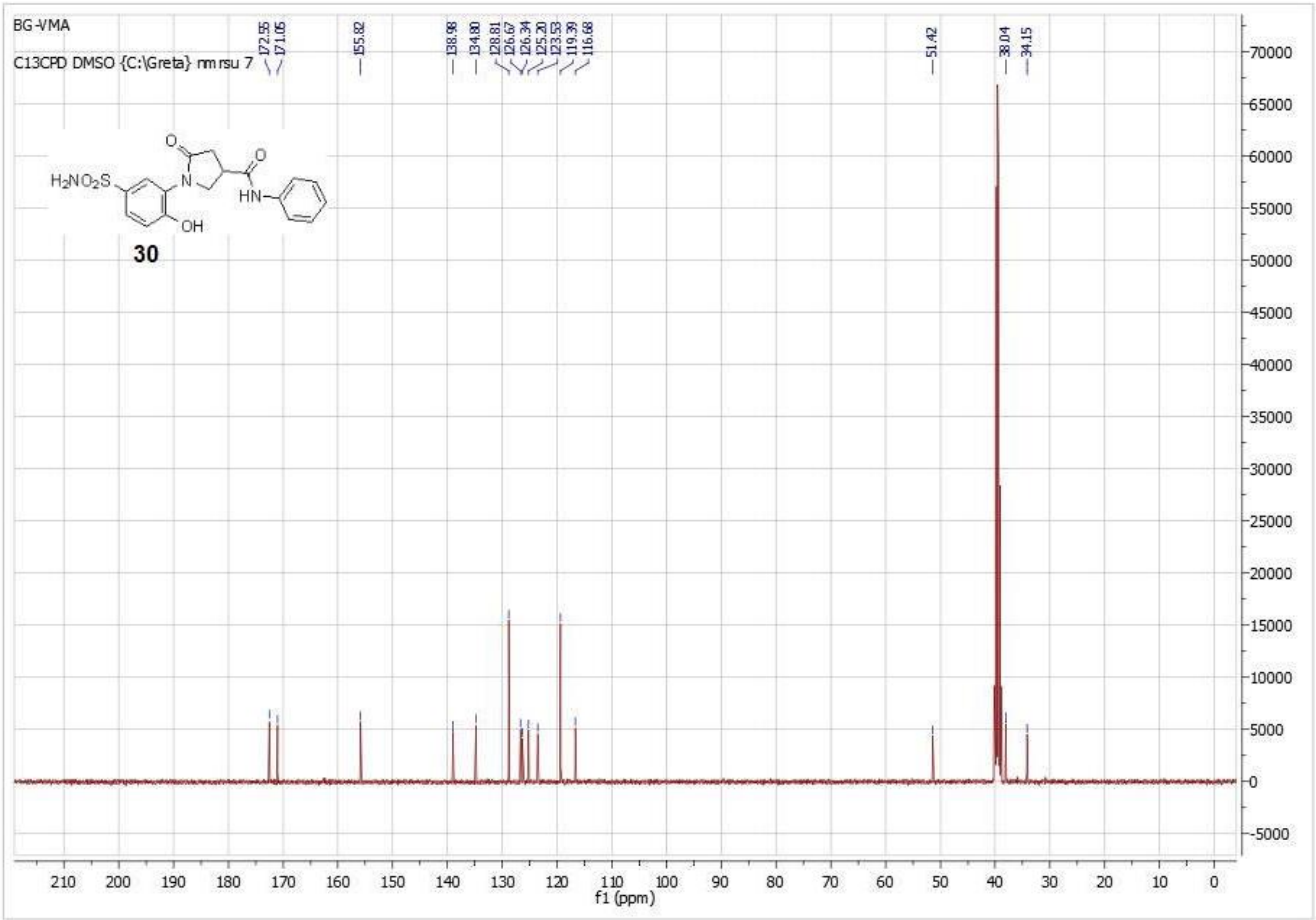

Figure S52. ^{13}C NMR of compound **30.**

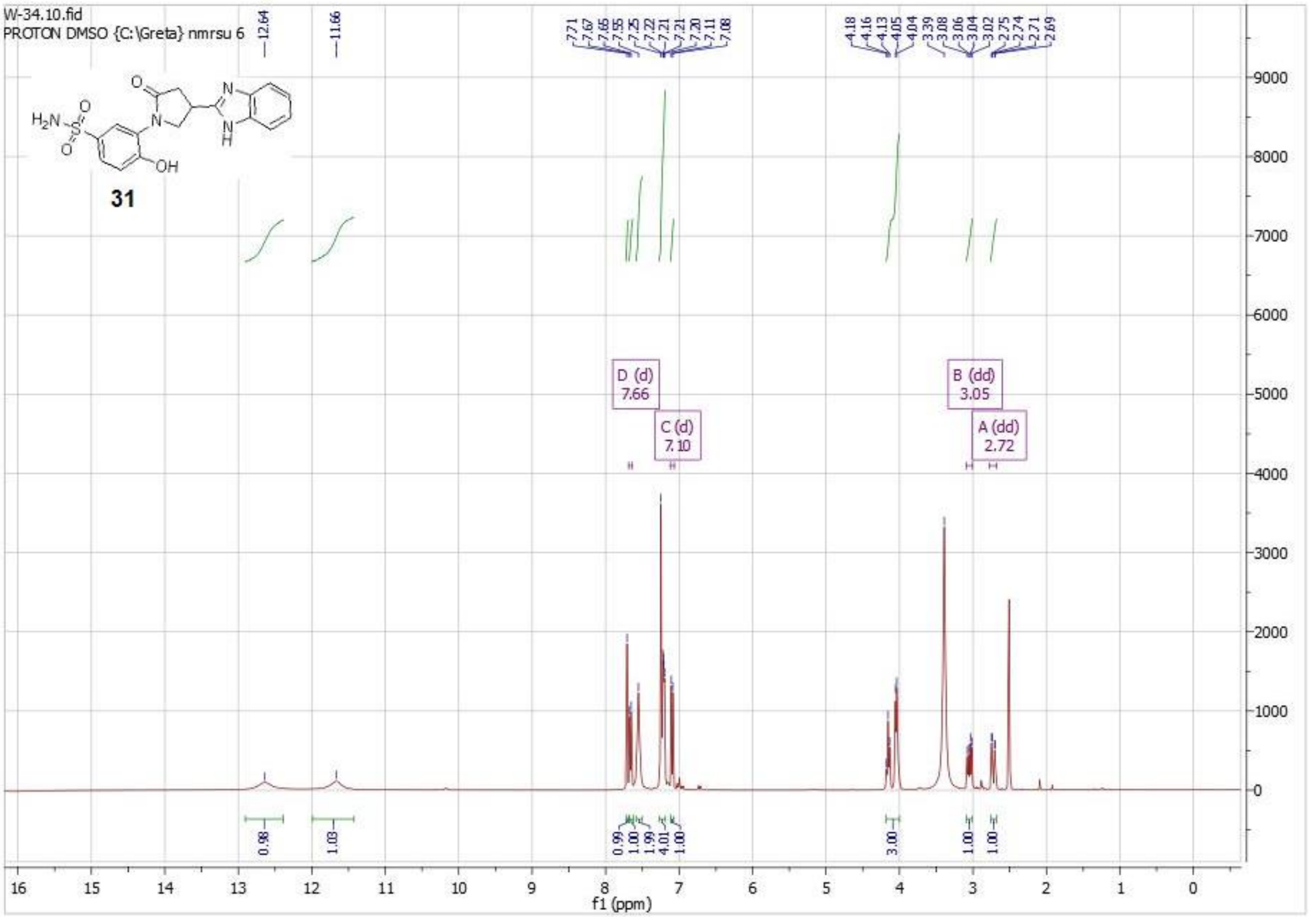

Figure S53. ^{1}H NMR of compound **31.**

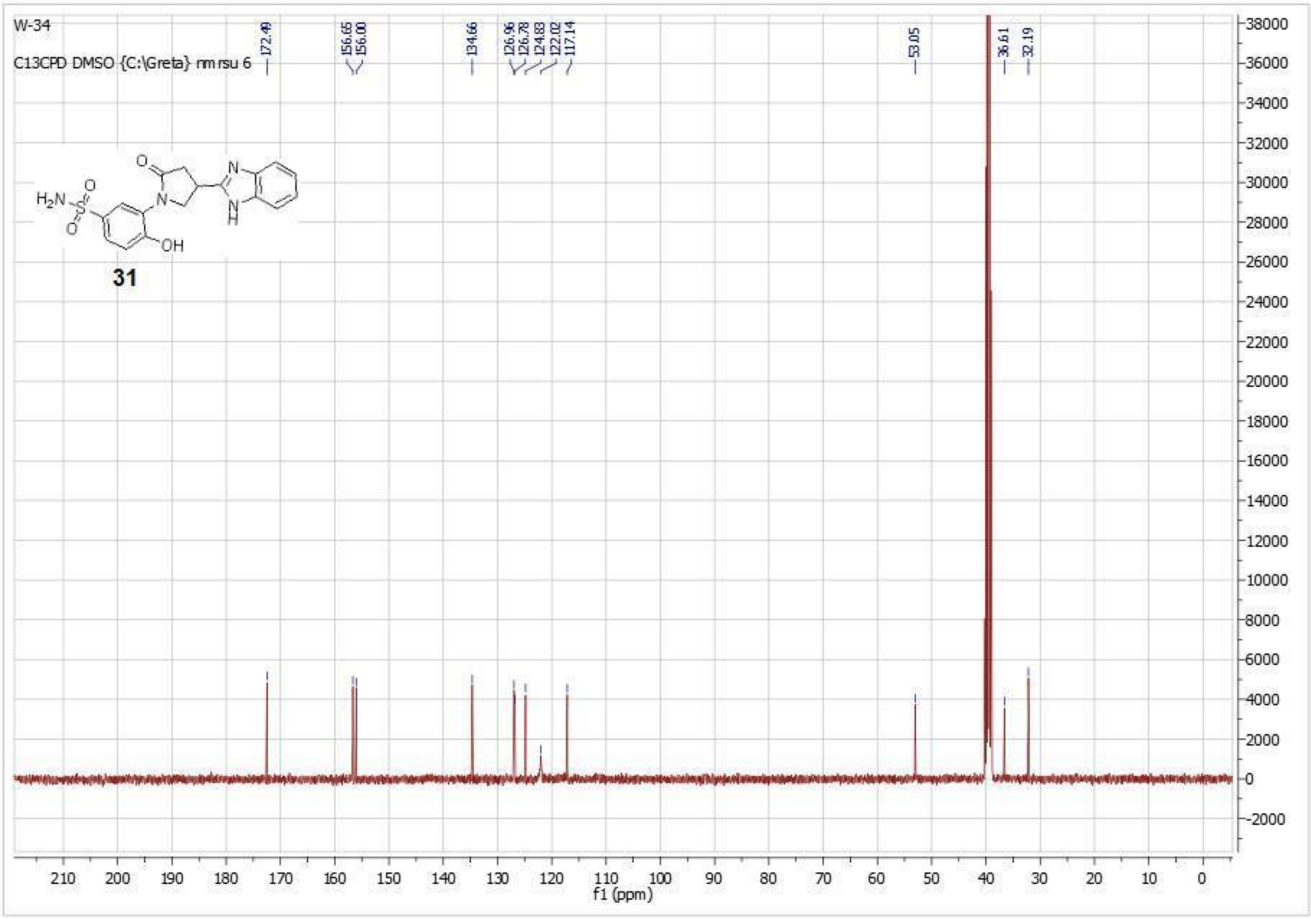

Figure S54. ^{13}C NMR of compound **31.**

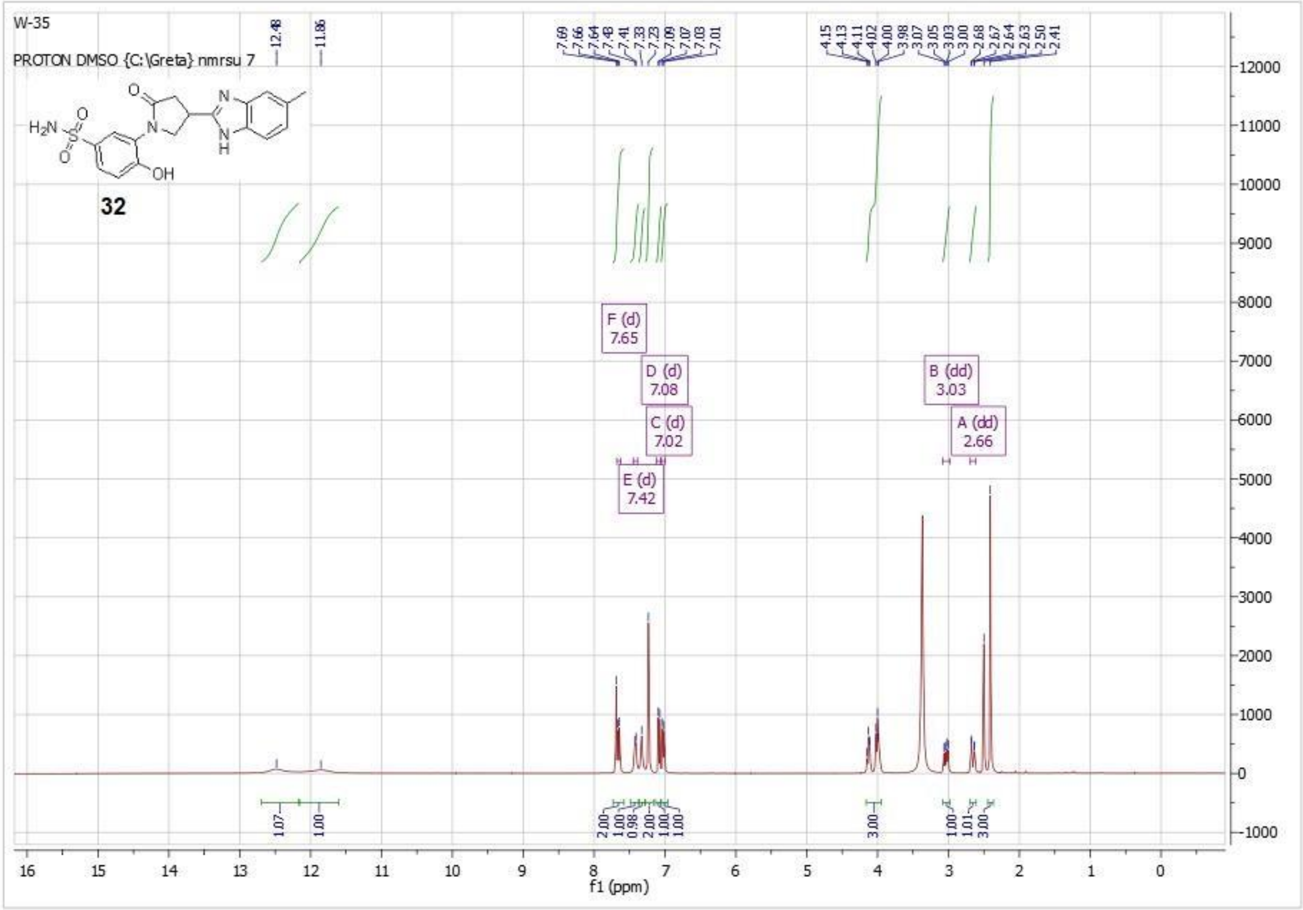

Figure S55. ^{1}H NMR of compound **32.**

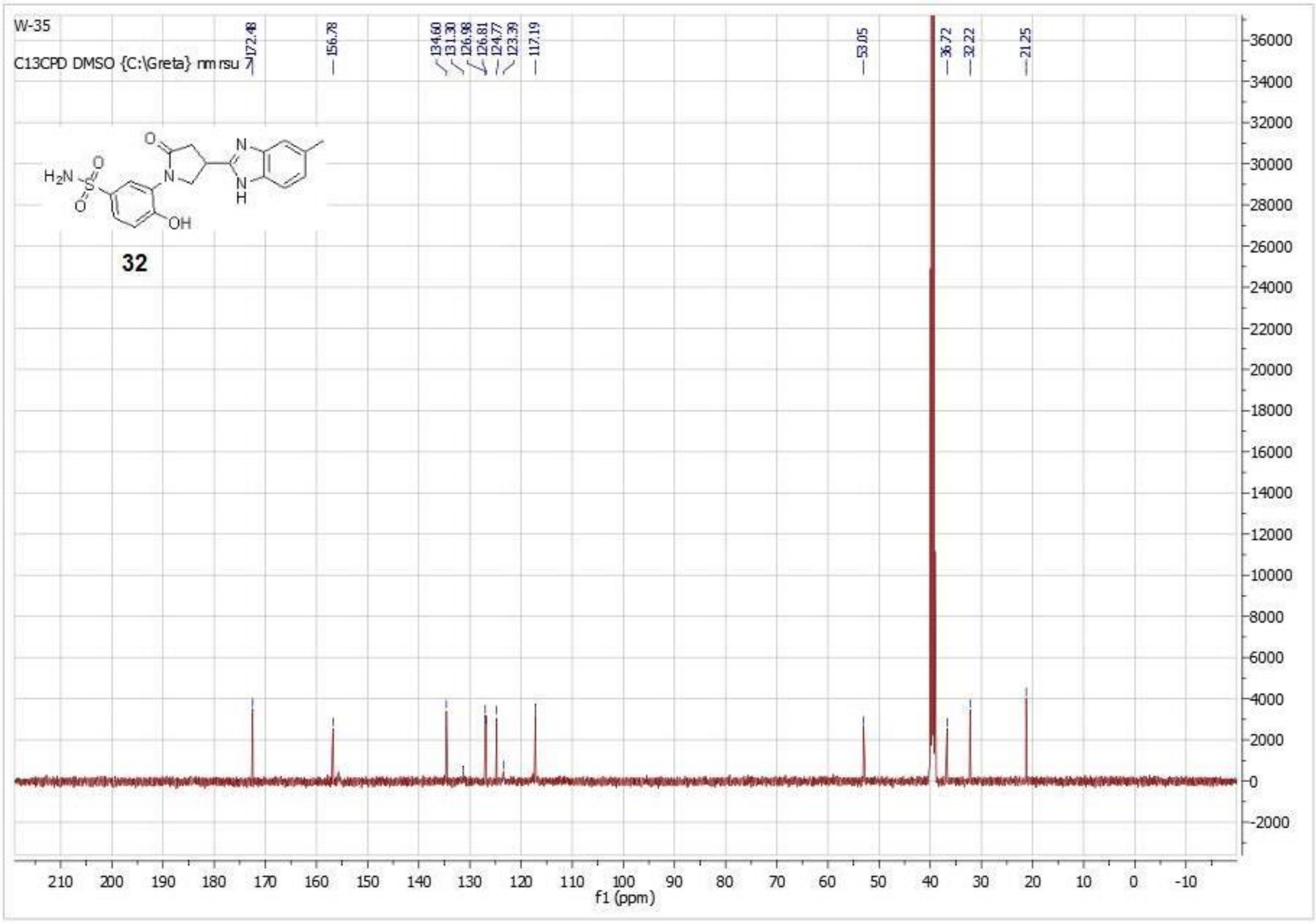

Figure S56. ^{13}C NMR of compound **32.**

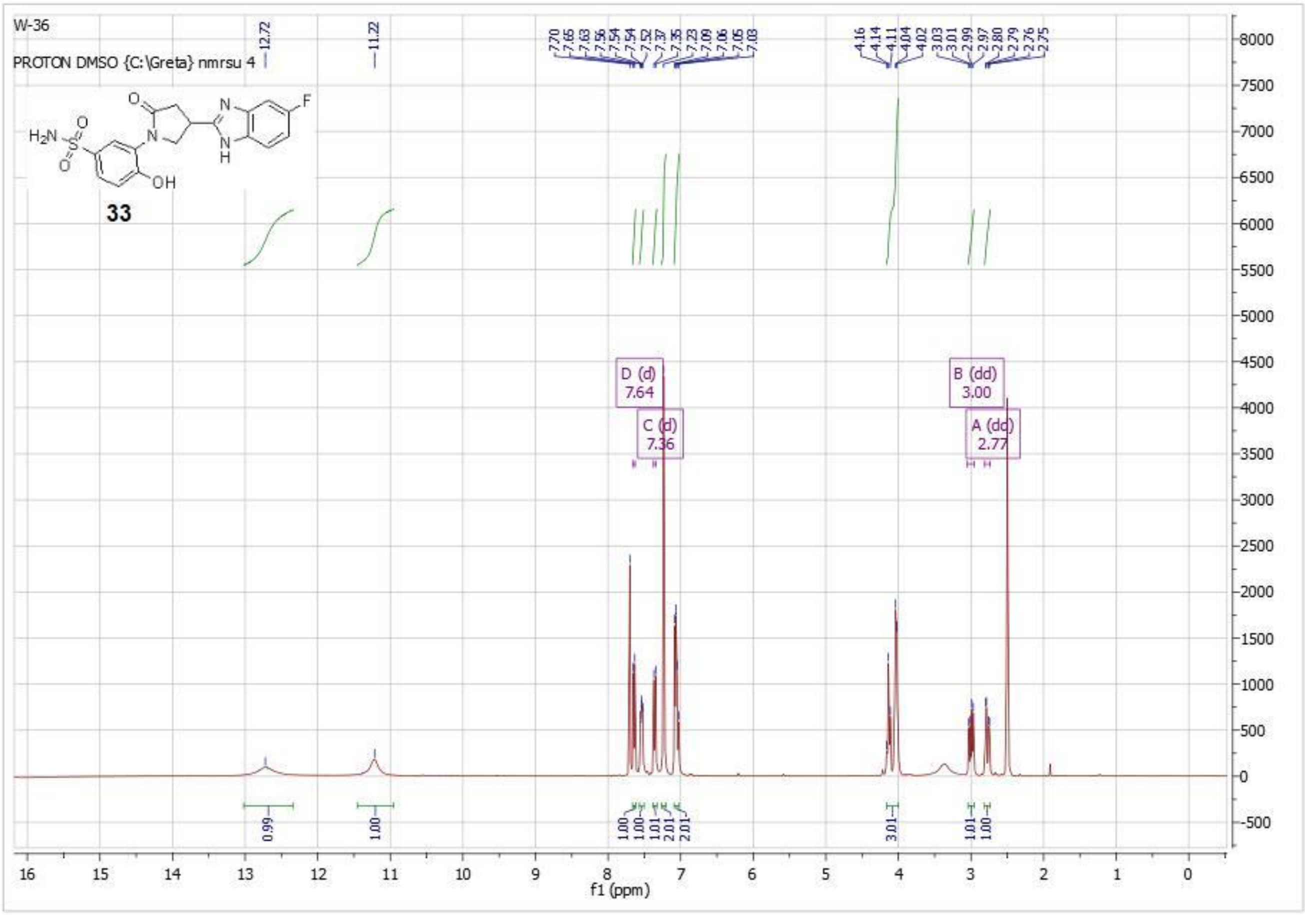

Figure S57. ^{1}H NMR of compound **33.**

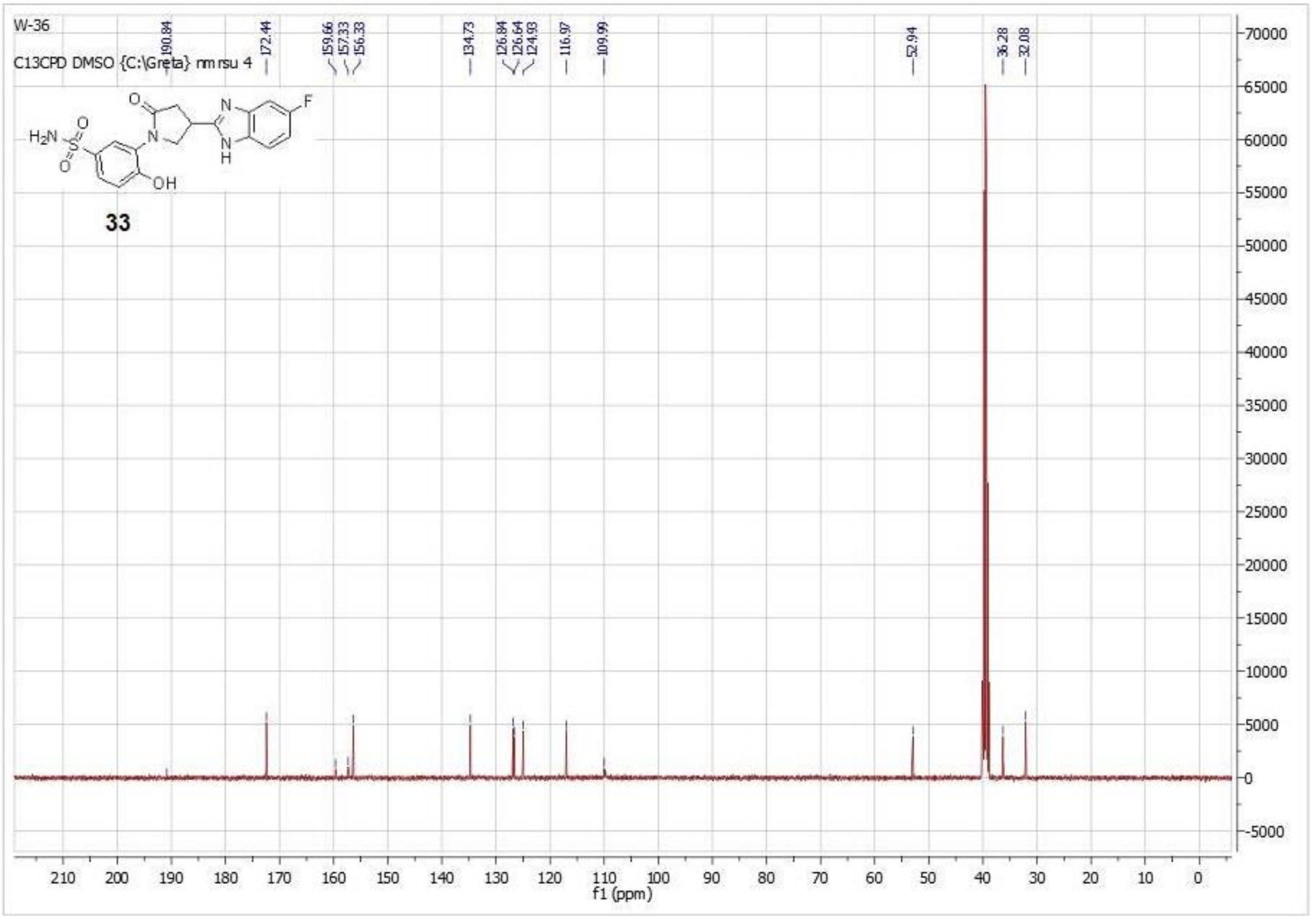

Figure S58. ^{13}C NMR of compound **33.**

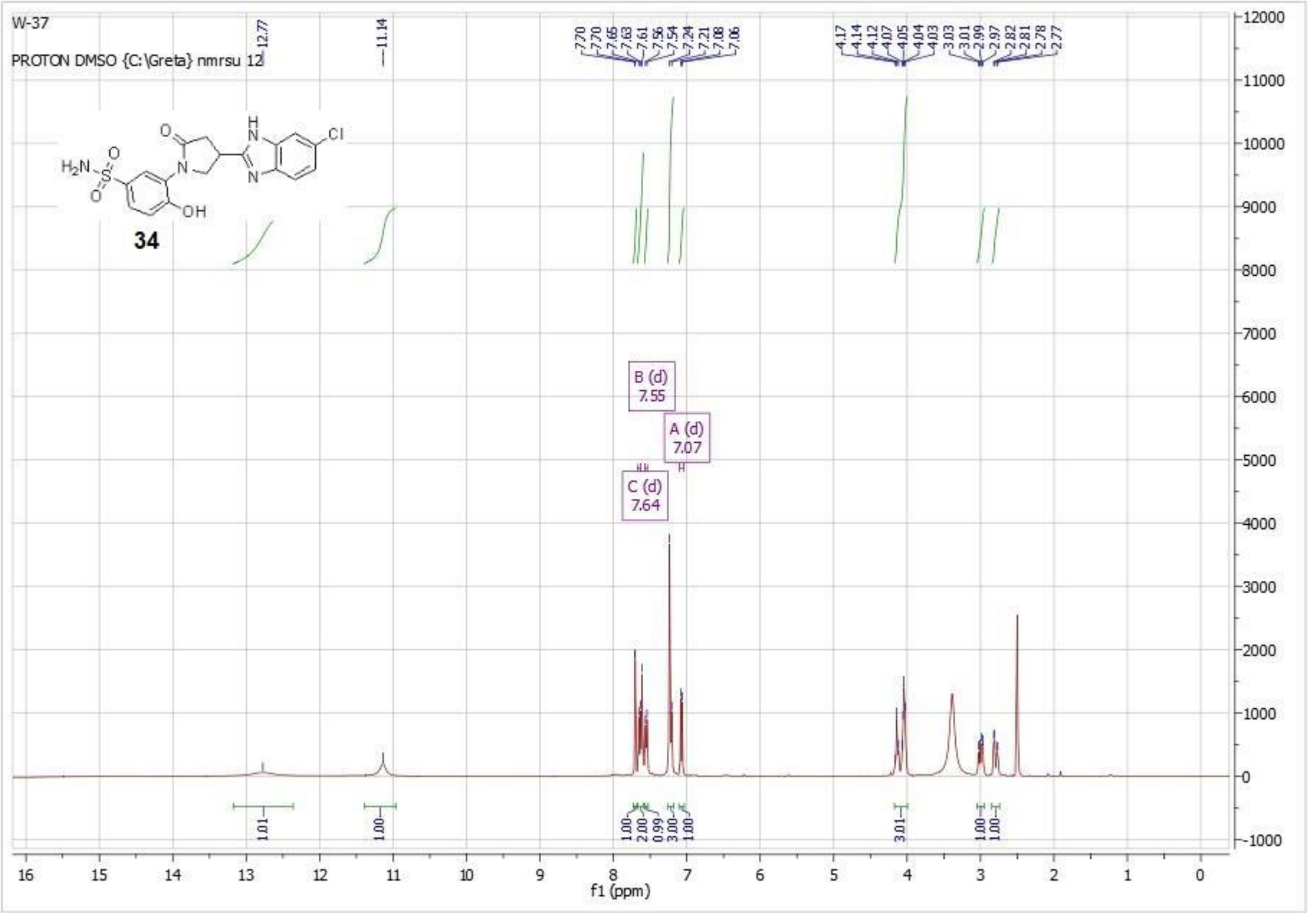

Figure S59. ^{1}H NMR of compound **34.**

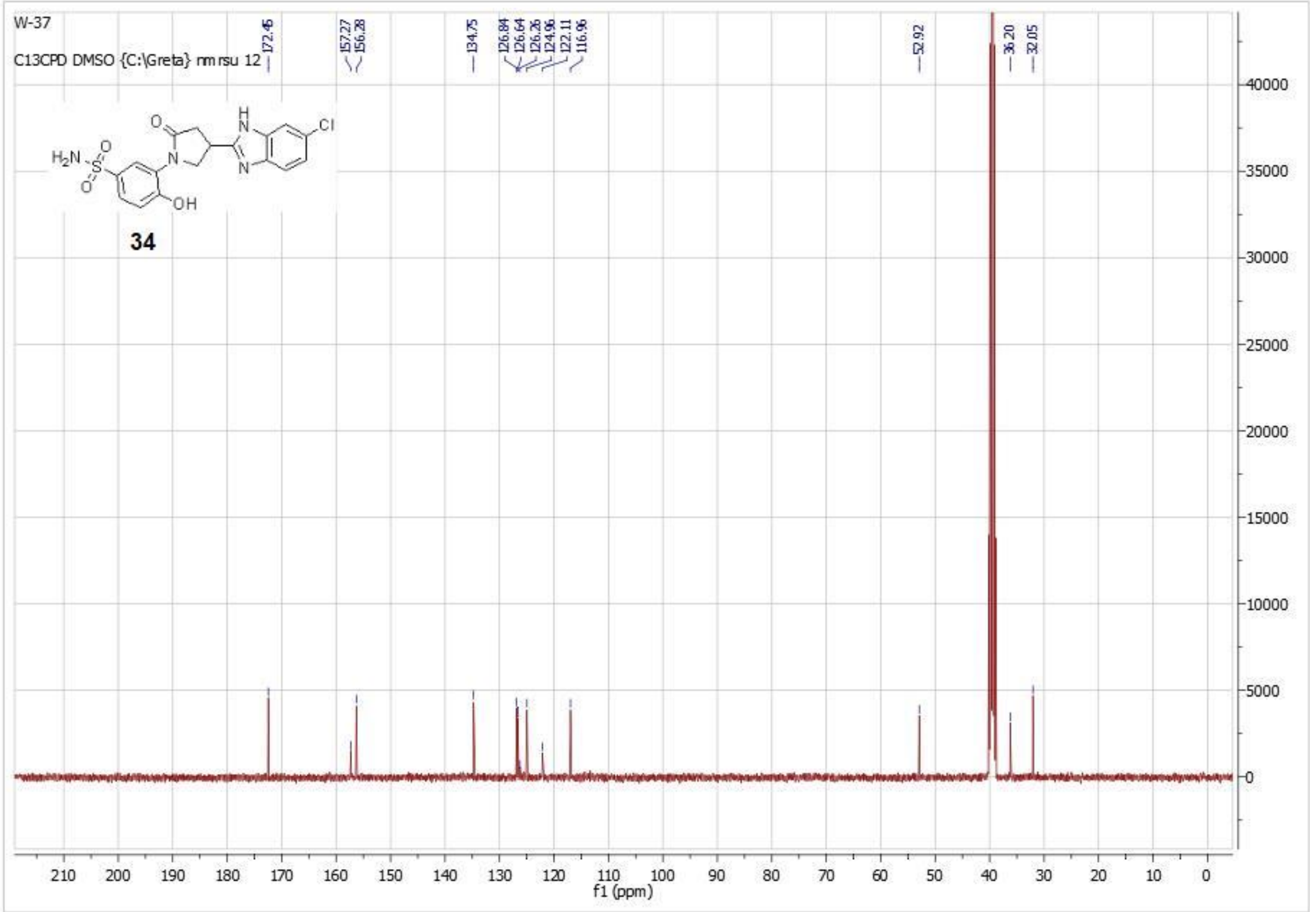

Figure S60. ^{13}C NMR of compound **34.**

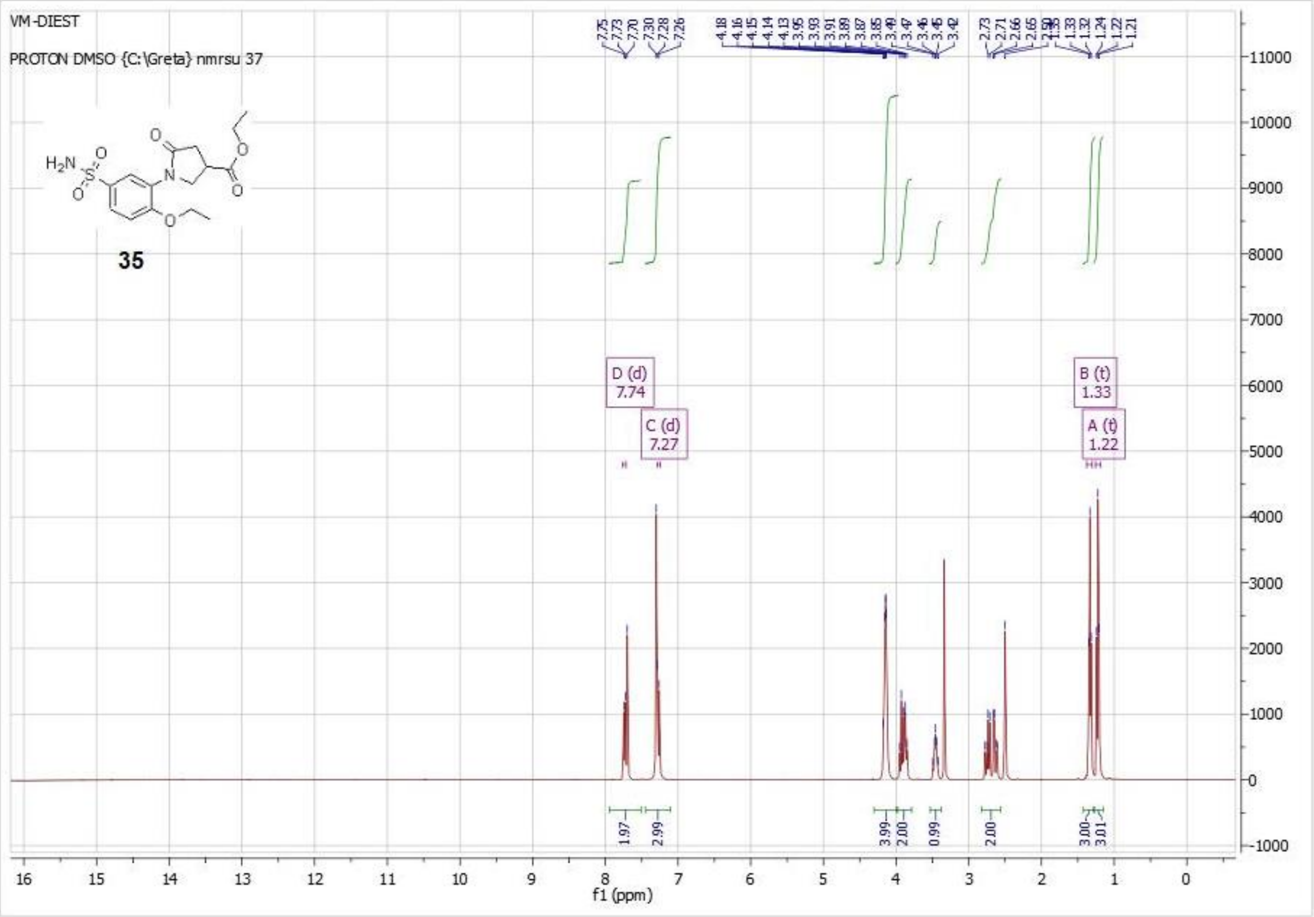

Figure S61. ^{1}H NMR of compound **35.**

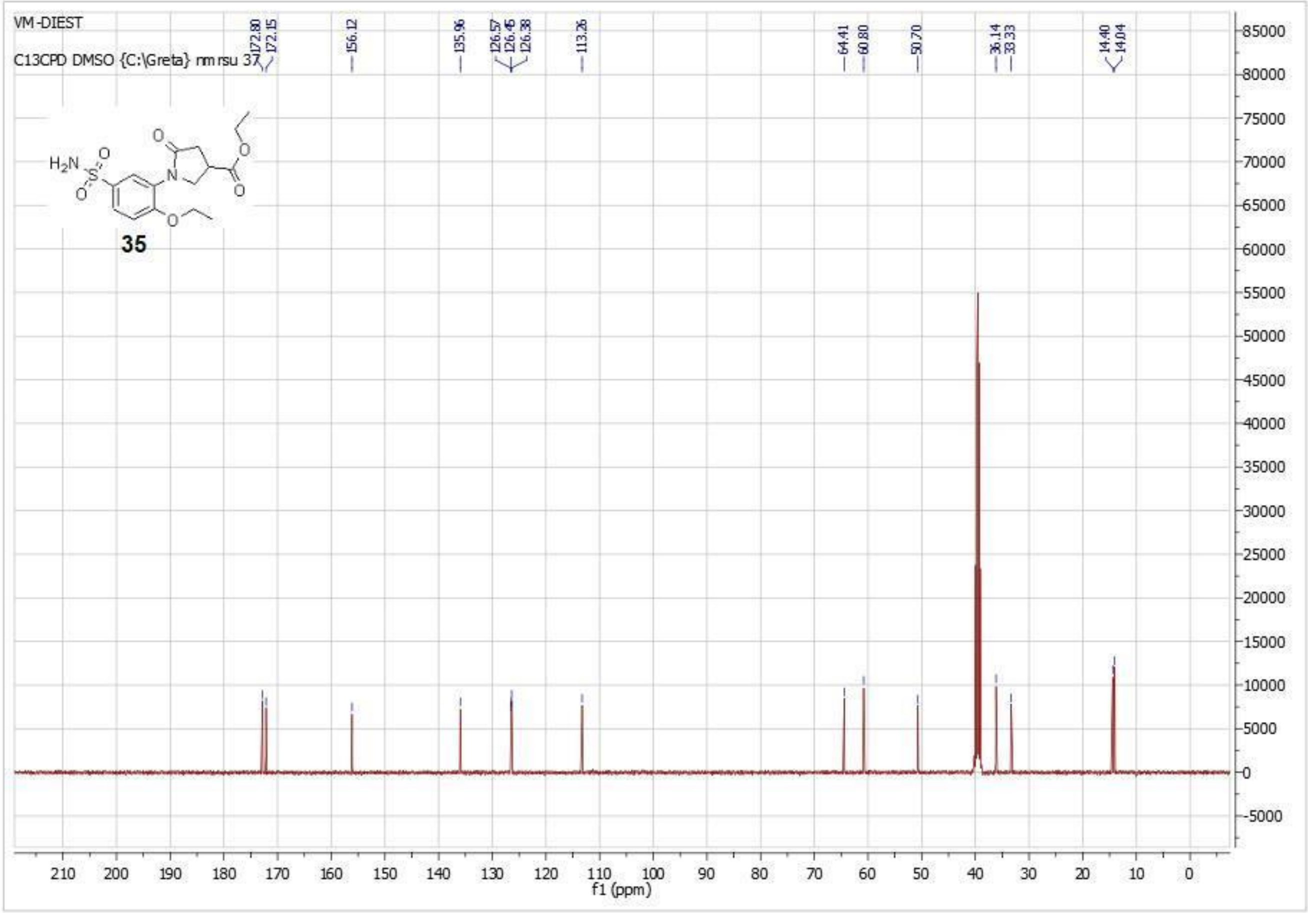

Figure S62. ^{13}C NMR of compound **35.**

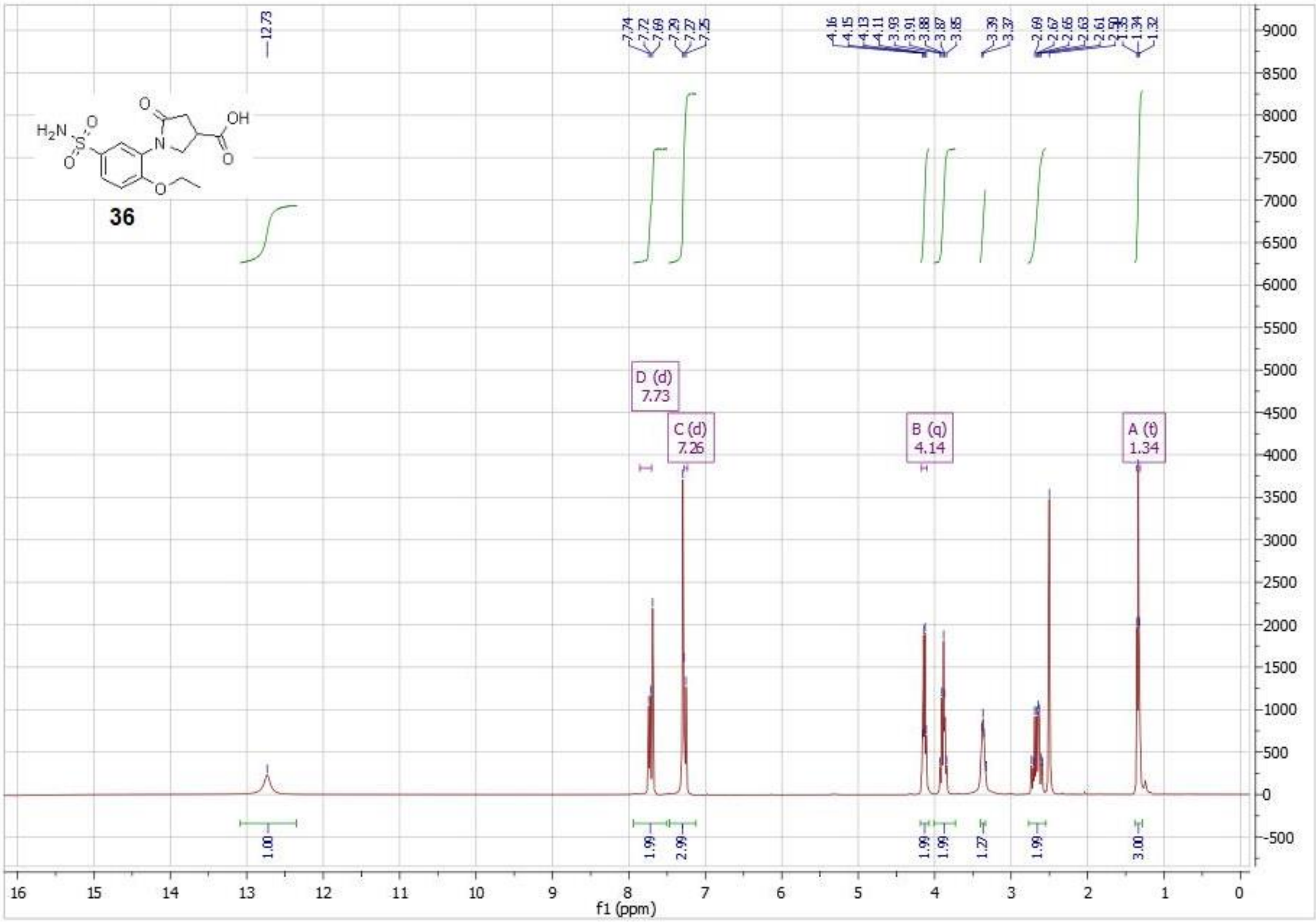

Figure S63. ^{1}H NMR of compound **36.**

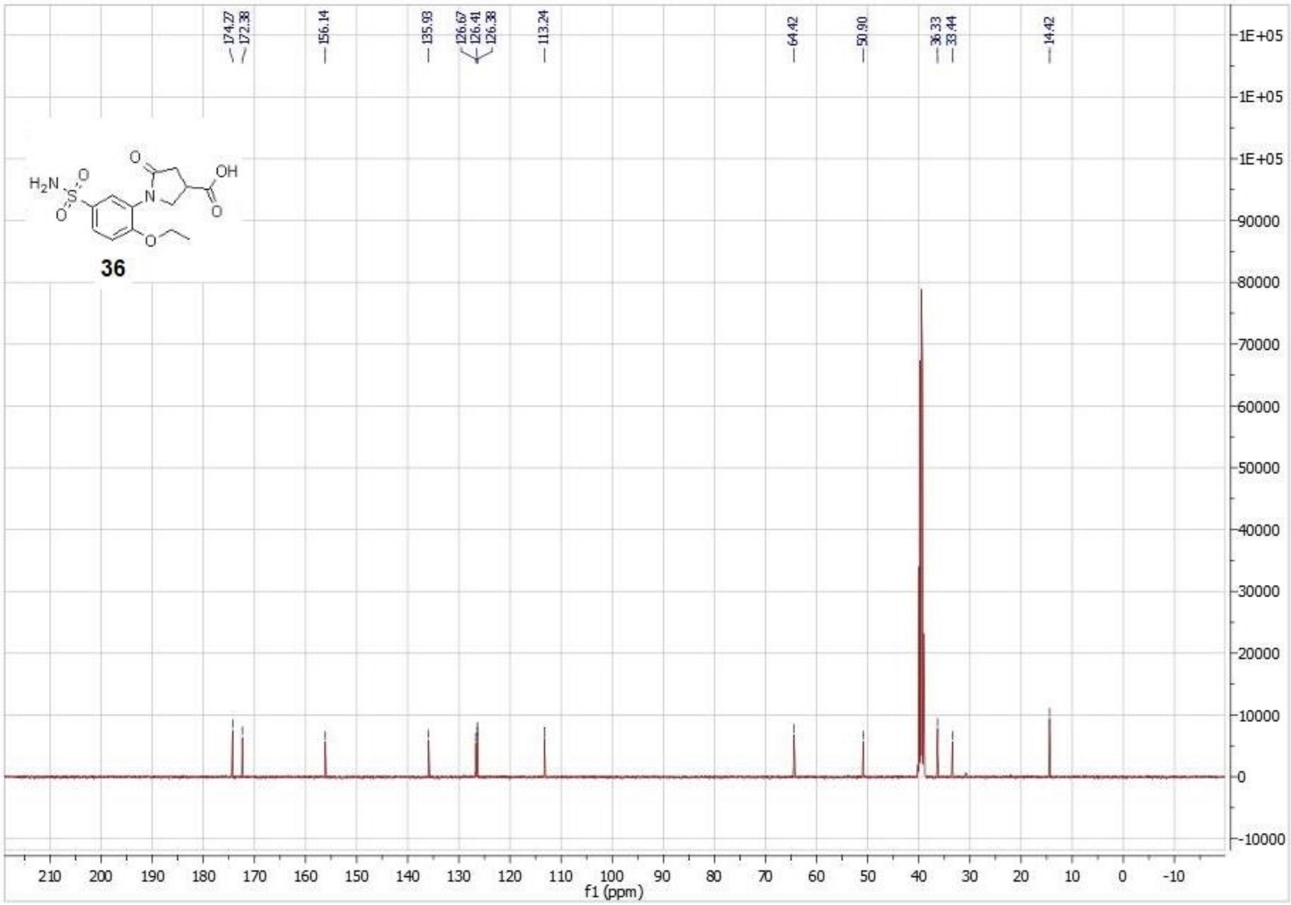

Figure S64. ^{13}C NMR of compound **36.**

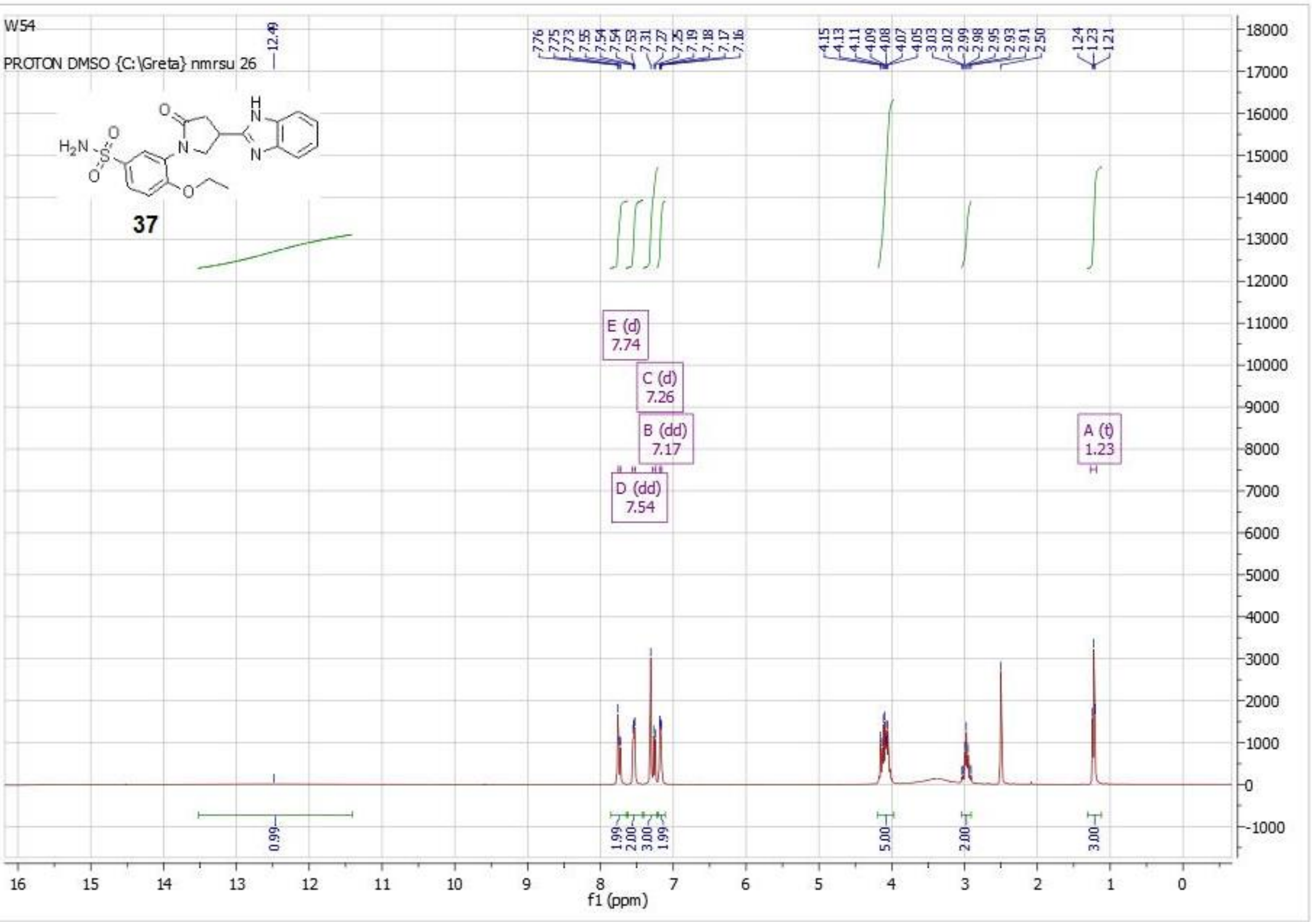

Figure S65. ^{1}H NMR of compound **37.**

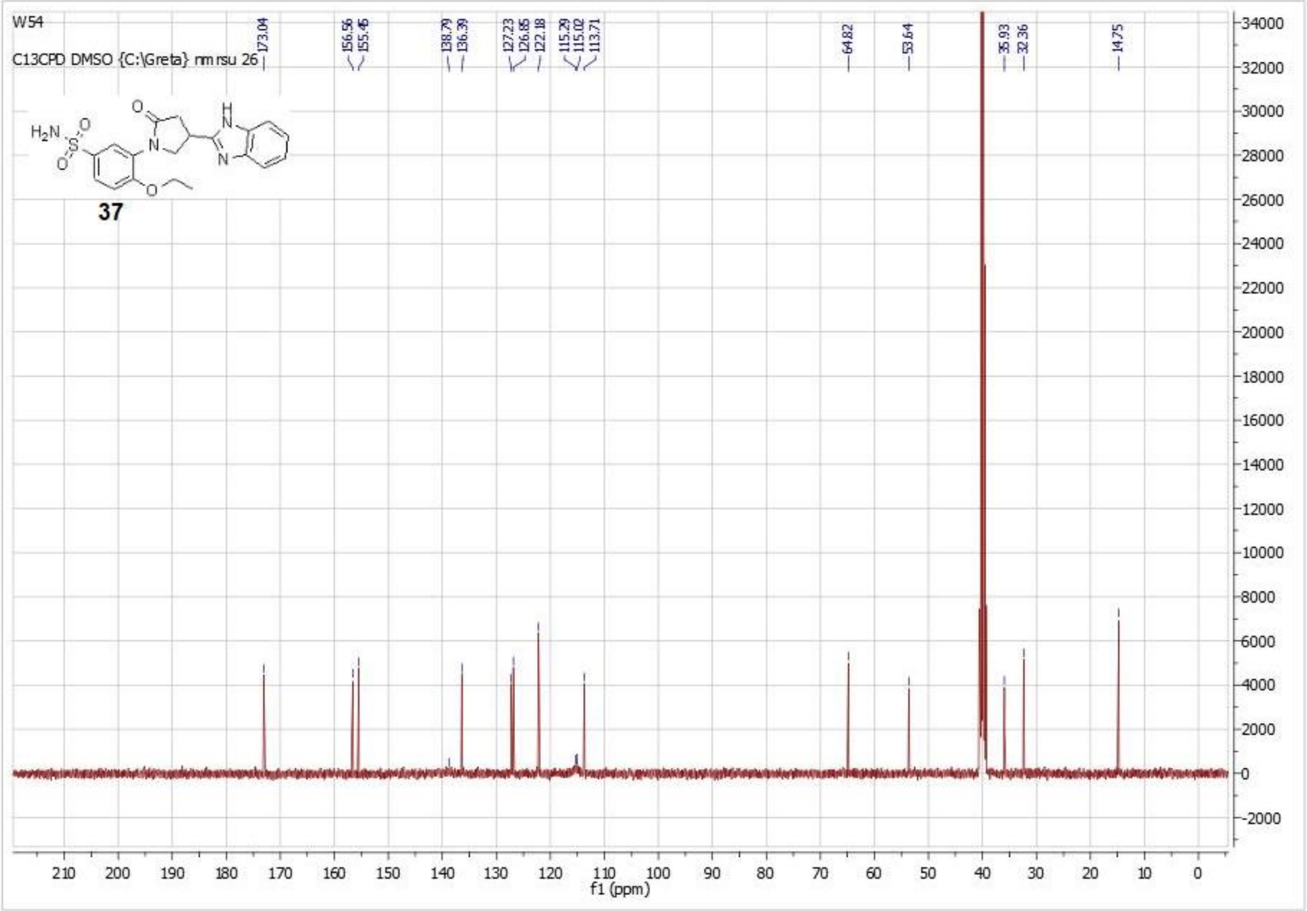

Figure S66. ^{13}C NMR of compound **37.**

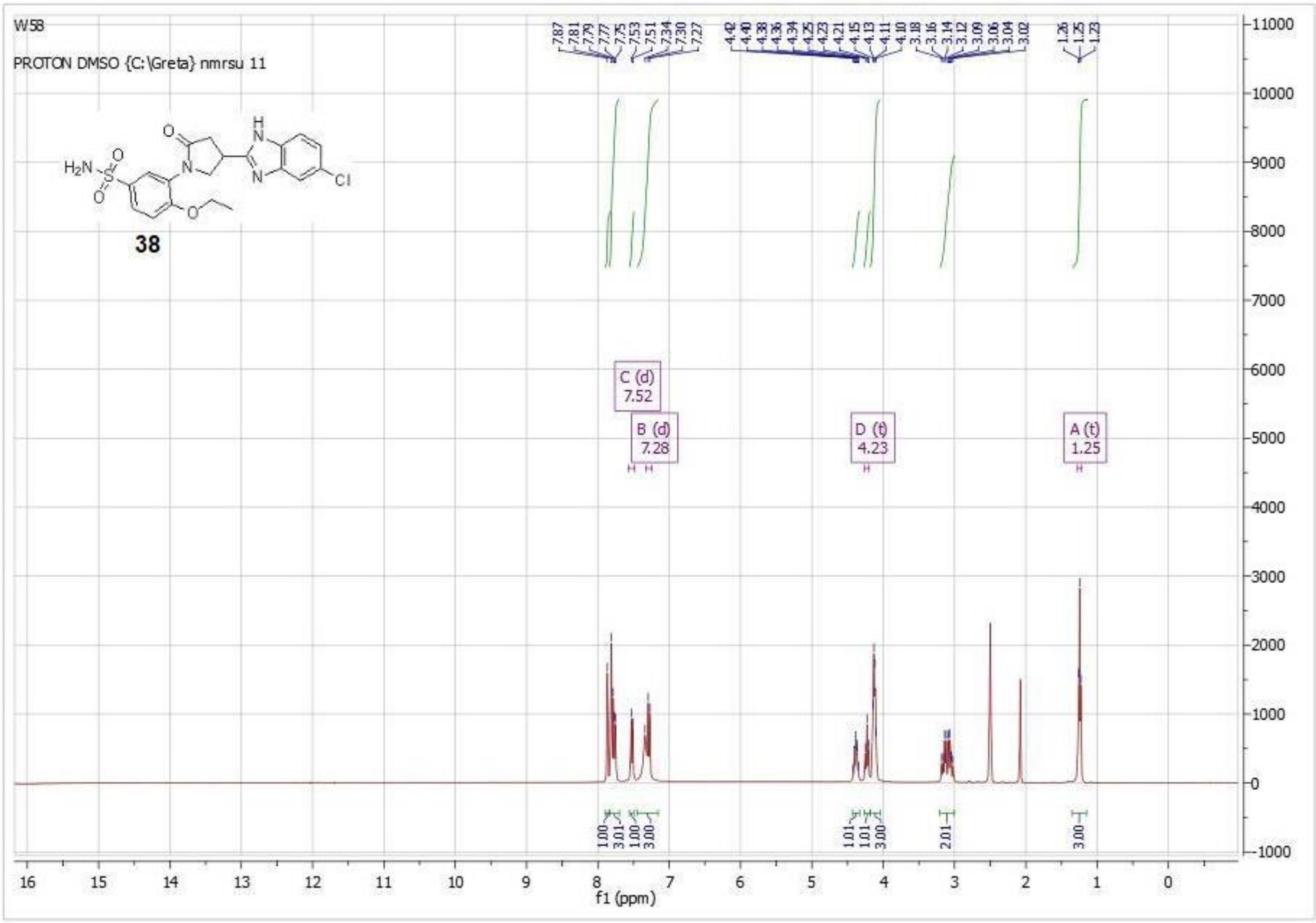

Figure S67. ^{1}H NMR of compound **38.**

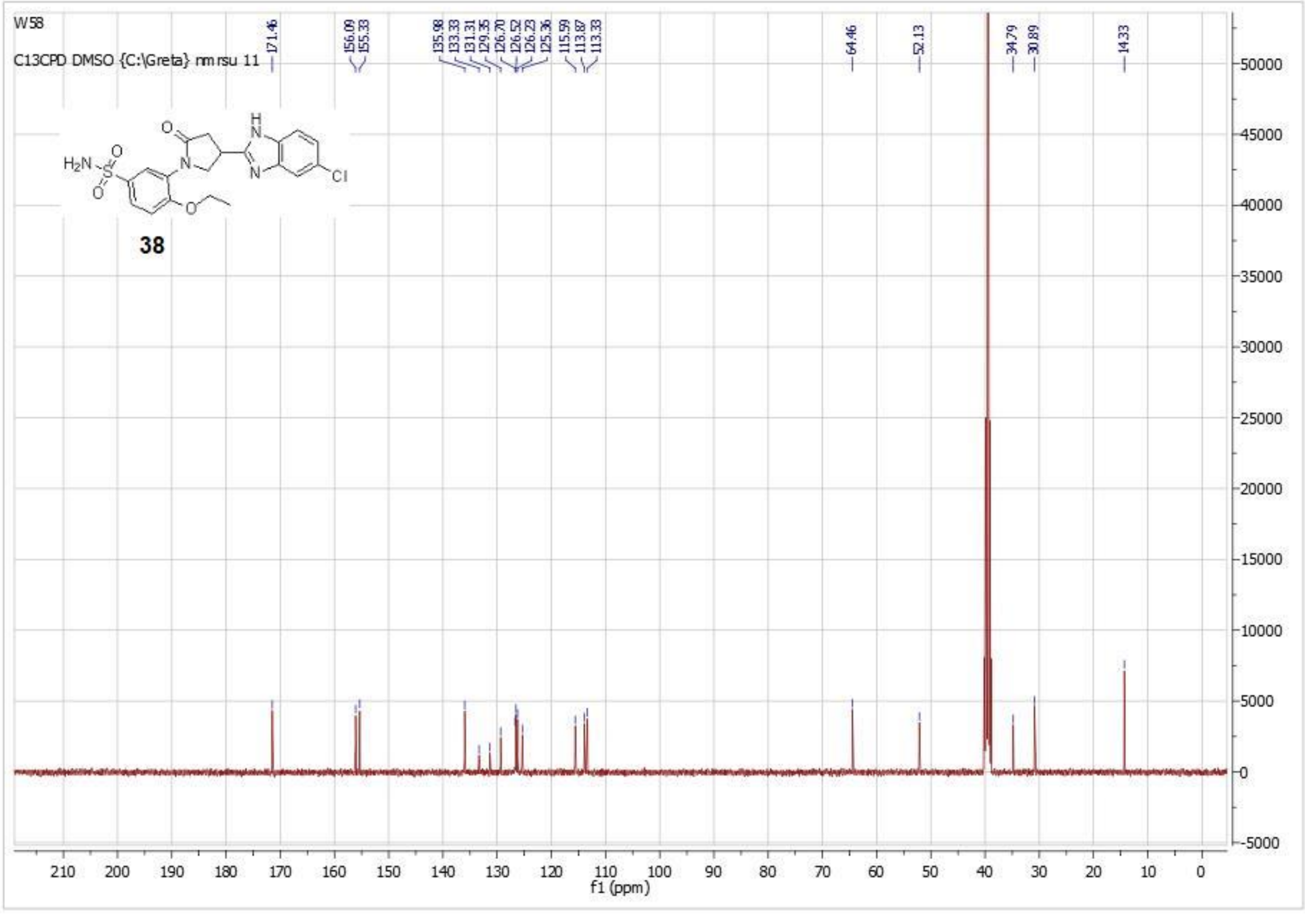

Figure S68. ^{13}C NMR of compound **38.**

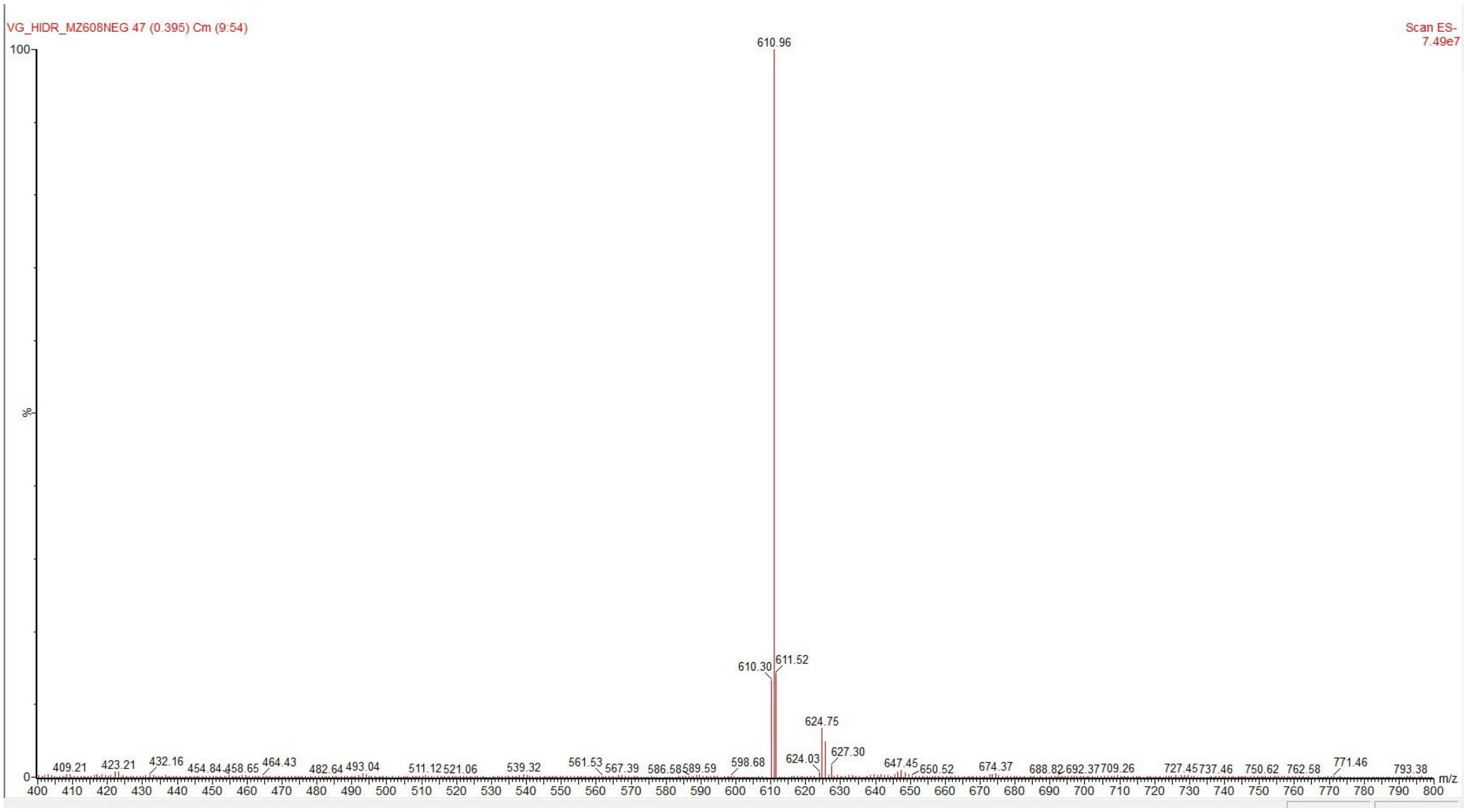

Figure S69. Mass spectrum of compound **18.**

Supplementary Table S1. Crystal structures of CAI and CAII complexes with compound **25** (W51).

Structure	CAI – W51	CAII – W51
Crystallization reservoir solution	Tris-HCl pH 8.5 0.1 M , sodium chloride 0.2 M , PEG4000 24%	Sodium Bicine pH 9.0 0.1M, Sodium Malonate pH 7.0 2M, Ammonium Sulfate 0.2M
Cryo protection solution	TrisHCl pH 8.5 0.1M, PEG4000 15%, ethylene glycol 20%	none
Data collection statistics		
Space group	P 21 21 21	P 1 21 1
Cell constants: a, b, c, Å; α, β, γ, (º)	a= 62.566Å, b= 71.122 Å, c=120.397 Å, α=β=γ=90º	a=42.19 Å, b=41.09 Å, c=71.93 Å, α=γ=90º, β=104.11º
Unique reflections: overall (outer shell)	71521 (3490)	82694 (2254)
Resolution range, Å	71.12-1.60	69.76-1.15
Completeness: overall (outer shell), %	99.9 (99.8)	96.9 (54.0)
Multiplicity: overall (outer shell)	13.2 (13.1)	6.6 (3.2)
I/σ: overall (outer shell)	33.6 (7.1)	19.1 (3.3)
Rmerge: overall (outer shell)	0.041 (0.380)	0.054 (0.291)
Wilson B-factor, Å2	22.7	10.8
Refinement statistics		
Reflections: work / test	64455 / 6979	74349 / 8329
Atom number:all / protein / Zn / ligand / solvent	4524 / 4086 / 2 / 72 / 356	2568 / 2208 / 1 / 36 / 323
Rcryst (Rfree)	0.212 / 0.240	0.140 / 0.167
RMSD: bond lengths, Å / bond angles, (°)	0.010 / 1.848	0.014 / 2.011
Ramachandran: favoured/ allowed/ outliers, %	97 / 3 / 0	91.8 / 8.2 / 0
average B-factors: all atoms/ main chain/ side chain/ Zn / ligand / solvent, $Å^2$	25.0 / 23.1 / 26.5 / 15.8 / 17.4 / 32.2	18.8 / 14.8 / 19.1 / 8.3 / 10.5 / 32.4
PDB ID	9HWN	9HWM
